# Synthesis and Anticancer Evaluation of Novel 7-Aza-Coumarine-3-Carboxamides

**DOI:** 10.3390/ijms24129927

**Published:** 2023-06-09

**Authors:** Alexey V. Trifonov, Almir S. Gazizov, Anipa S. Tapalova, Lyudmila K. Kibardina, Nurbol O. Appazov, Alexandra D. Voloshina, Anastasiia S. Sapunova, Anna P. Luybina, Gulmira M. Abyzbekova, Alexey B. Dobrynin, Igor A. Litvinov, Akerke K. Tauekel, Sholpan O. Yespenbetova, Alexander R. Burilov, Michail A. Pudovik

**Affiliations:** 1 Arbuzov Institute of Organic and Physical Chemistry, FRC Kazan Scientific Center, Russian Academy of Science, Arbuzova Str., 8, Kazan 420088, Russia; xahter91@gmail.com (A.V.T.); kibardina@iopc.ru (L.K.K.); micrbi@iopc.ru (A.D.V.); luybina@iopc.ru (A.P.L.); aldo@iopc.ru (A.B.D.); litvinov@iopc.ru (I.A.L.); burilov@iopc.ru (A.R.B.); pudovik@iopc.ru (M.A.P.); 2 Korkyt Ata Kyzylorda University, Aiteke Bi Street, 29A, Kyzylorda 120014, Kazakhstan; anipa52@mail.ru (A.S.T.); nurasar.82@mail.ru (N.O.A.); abizgul@mail.ru (G.M.A.); sholpan-sultan@mail.ru (S.O.Y.); 3 Department of Oil, Chemistry and Nanotechnology, Kazan National Research Technological University, Karl Marx Str., 68, Kazan 420015, Russia

**Keywords:** aza-coumarine, amides, cytotoxicity, selectivity, anti-tumor

## Abstract

Herein, we report the design and synthesis of novel 7-aza-coumarine-3-carboxamides via scaffold-hopping strategy and evaluation of their in vitro anticancer activity. Additionally, the improved non-catalytic synthesis of 7-azacoumarin-3-carboxylic acid is reported, which features water as the reaction medium and provides a convenient alternative to the known methods. The anticancer activity of the most potent 7-aza-coumarine-3-carboxamides against the HuTu 80 cell line is equal to that of reference Doxorubicin, while the selectivity towards the normal cell line is 9–14 fold higher.

## 1. Introduction

**Scheme 1:**
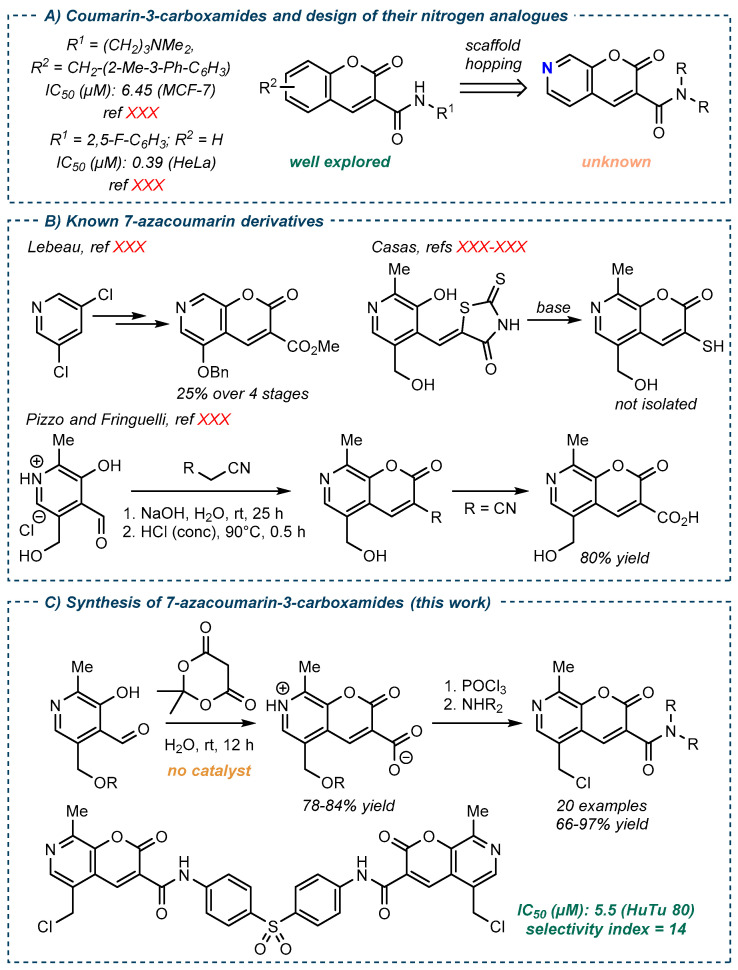
Examples of coumarin-3-carboxamides with antitumor activity and scaffold hopping to nitrogen analogs (**A**); the only known 7-aza-coumarin derivatives (**B**); synthesis of novel 7-aza-coumarin-3-carboxamides proposed in this work and the most potent compound (**C**).

Coumarins are widespread structural motifs in natural products and exhibit a broad range of biological activities [1,2,3]. Their anticancer properties [4,5,6] have attracted considerable interest and have been extensively studied by multiple research groups. One of the promising classes of coumarin derivatives is coumarin-3-carboxamides, which have been the object of numerous in-depth investigations [7,8,9,10,11]. A large series of coumarin-3-carboxamides have been obtained and tested against various types of cancer cell lines so far. Among them, promising candidates for further development have been reported (Scheme 1A) [11,12].

A powerful strategy for discovering novel biologically relevant compounds is scaffold hopping [13,14], a concept that was first introduced by Schneider and coauthors [15] in 1999. Scaffold-hopping methods imply the modification of the core structure of the molecule with known activity to give a novel chemotype while trying to conserve the biological properties of the parent compound. The simplest modification is replacing or swapping carbon and heteroatoms in a backbone ring (1° hop, according to Sun and coworkers [14]), which in the case of coumarins, would derive their heterocyclic analogs. The incorporation of a nitrogen atom into aromatic moiety is of special interest. It may decrease compounds’ susceptibility to oxidative metabolism [16], thus improving their bioactivity profile. Additionally, the ability of nitrogen atoms to form extra hydrogen bonds may also play a role in binding to the active site of a biological target. For example, the replacement of aromatic carbon atoms by nitrogen has been successfully employed for the development of anticancer agents based on azaindole [17], imidazo[1,2-a]pyridinone [18], and pyrido[1,2-a]pyrimidin-4-one [19] scaffolds.

Thus, we wanted to investigate if a similar replacement of aromatic carbon by nitrogen would allow us to enhance the anticancer properties of coumarin-3-carboxamides. Notably, azacoumarins represent an interesting yet underexplored family of compounds. Although multiple regioisomeric azacoumarins are possible, only a few of them have been obtained so far. The vast majority of available bioactivity data refers to 1-azacoumarins (2-quinolones) [20], which have been extensively studied due to their remarkable anticancer properties [21,22,23,24,25].

Taking all of the above into account, we first aimed at the synthesis of hitherto unknown 7-azacoumarin-3-carboxamides and the evaluation of their cytotoxicity towards cancer cell lines. The literature survey revealed that only a limited number of 7-azacoumarins are known until now (Scheme 1B). Lebeau and coworkers reported the 4-stage synthesis of methyl ester of 7-azacoumarin-3-carboxylic acid starting from 3,5-dichloropyridine in 25% overall yield [26]. Casas and coworkers described the thallium(III) [27] and gold(I) [28] complexes of the 3-mercapto-7-azacoumarin derivative. However, the ligand was not isolated in pure form. Finally, Pizzo and Fringuelli developed the method of synthesis of 3-substituted 7-azacoumarins, including 7-azacoumarin-3-carboxylic acid, via one-pot two-stage Knoevenagel condensation of pyridoxal with CH-acids [29].

Based on these data, we proposed the synthetic route to desired compounds as depicted in Scheme 1C. As a result of our studies, herein we report a two-stage synthesis of diverse 7-azacoumarin-3-carboxamides and evaluation of their cytotoxicity towards tumor and normal cell lines. Taking into account that cervical cancer is one of the most common gynecologic cancers worldwide [30,31], the M-HeLa cell line was chosen for testing. Additionally, the cytotoxicity towards HuTu 80 cells was evaluated. Although duodenal adenocarcinoma is a rare type of tumor, its aggressive progression makes surgical resection the most used therapy [32]. Certainly, a lack of chemotherapeutic treatment for this type of cancer is a serious problem. Additionally, both M-HeLa and HuTu 80 cell lines are widely used for cytotoxicity evaluation, which facilitates the comparison of the obtained data with literature values. Doxorubicin and 5-fluorouracil were chosen as reference compounds. This choice was guided by following considerations: (a) both Doxorubicin and 5-fluorouracil are widely used for the treatment of various types of cancer, so a comparison of novel compounds with well-known marketed drugs can be made; (b) there are many published studies in which these compounds were employed as reference, thus a comparison with literature data becomes possible.

The cytotoxicity of the most potent compounds was comparable to that of “golden standard” Doxorubicin (IC_50_ 2.9–5.5 µM vs. 3.0 ± 0.2 µM for Doxorubicin), while the toxicity towards normal cell line was much lower (selectivity index, SI = 10–14 vs. SI = 1 for Doxorubicin). Additionally, we report an improved synthesis of 7-azacoumarin-3-carboxylic acid, which features a simpler and “greener” procedure than known ones, and it does not require any catalysts and uses water as a reaction medium.

## 2. Results and Discussion

### 2.1. Chemistry

We initiated our studies by searching the optimal conditions for the synthesis of a key intermediate, 7-aza-coumarin-3-carboxylic acid **3a**, using easily available pyridoxal **1** or pyridoxal hydrochloride **1·HCl** as a starting compound. As was mentioned above, the synthesis of compound **3a** was achieved earlier via base-promoted Knoevenagel condensation of pyridoxal with malononitrile and the subsequent acidic hydrolysis of intermediate nitrile [29]. We speculated that the usage of malonic acid esters instead of malononitrile would allow us to obtain acid **3a** directly, thus avoiding a hydrolysis step. Pleasingly, simply keeping the water solution of pyridoxal or pyridoxal hydrochloride and Meldrum’s acid at room temperature furnished the desired 7-aza-coumarin-3-carboxylic acid **3a** in fairly high yield (Table 1, entry 1). The structure of acid **3a** was confirmed by X-ray analysis of its potassium salt (see Supplementary Information, Table S1, Figure S7 for additional details and X-ray data). Moreover, the phosphoryl derivative **3b** could be obtained from pyridoxal-5-phosphate using the same procedure (Table 1, entry 2). We tried to replace the Meldrum’s acid with more accessible malonic acid, however, without success. Although both non-catalytic and catalytic variants were tested, the yield of compound **3a** did not exceed 10% (Table 1, entry 3). We also tested dimedone in this reaction, which allowed us to obtain 7-azacoumarin **4** (Table 1, entry 4). Notably, the reaction of acetylacetone resulted in either 7-azacoumarin derivative **5** or fluoropyridine **6,** depending on the reaction conditions (Table 1, entries 5,6).

The structures of compounds **4** and **5** were additionally confirmed by X-ray analysis (see Supplementary Information, Table S1, Figures S1–S3 for detailed X-ray data). Interestingly, compound **4** exhibited axial chirality similar to previously described 3-azaxanthene derivatives [33] and was isolated as 1:1 mixtures of diastereomers.

Having optimized the synthesis of 7-azacoumarin-3-carboxylic acid **3a**, we screened the most optimal method of its conversion to the target amides. A number of amide bond-forming reactions and coupling reagents have been developed so far [34,35,36], including the electrosynthetic [37] and photocatalytic [38] methods. However, the most straightforward and mature approach via transient acyl halide formation was proven to be the most effective in terms of reagents availability, reaction scope, and product yield in our case. Preliminary studies with compound **3a** and aniline indicated that the phosphorus oxychloride was superior to other chlorinating reagents tested (thionyl halides and oxalylchloride). Importantly, this approach allowed the simultaneous replacement of the hydroxyl group by chlorine atom in compound **7a**, which is useful if further modification via nucleophilic substitution is considered. Interestingly, the phosphoryl-substituted acid **3b** could also be used instead of compound **3a**, leading to the same amide **7a**.

**Scheme 2:**
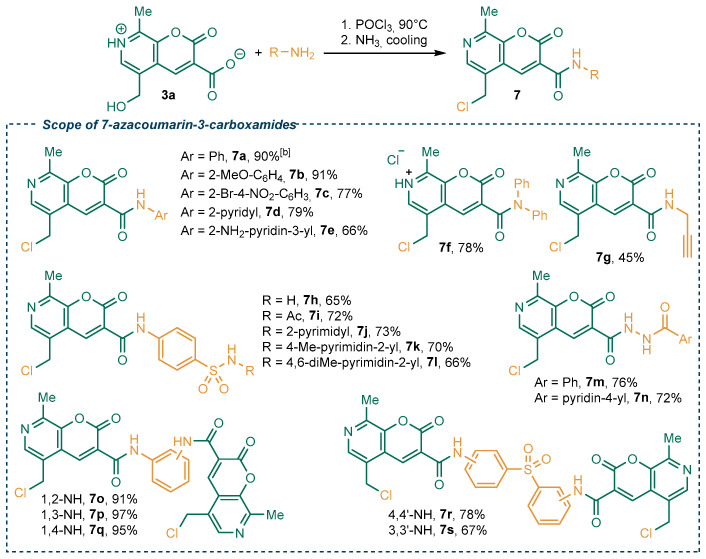
Synthesis of amides **7**^[a]^ [a] Isolated yield is given [b], and it was obtained from **3b** via the same procedure.

With these conditions in hand, we expanded the scope of the reaction using various aromatic and heteroaromatic amines (Scheme 2, see Supplementary Information, Figures S8–S55 for the NMR spectra of the obtained compounds). Both electron-donating and electron-withdrawing substituents in anilines were well tolerated, resulting in the compounds **7b,c** in a 77–91% yield. More sterically demanding diphenylamine also provided compound **7f** in high yield. Amino- and diaminopyridines furnished the desired amides **7d,e** in good yields (66–79%). Notably, only one amino group was substituted in the case of diaminopyridines. Given the importance and versatility of alkyne-azide cycloaddition in current medicinal chemistry and drug design, we additionally tested a propargylamine in these conditions. Pleasingly, the triple bond remained intact under reaction conditions, and the corresponding amide **7g** was isolated in a 45% yield. A series of amides **7h–l** possessing aryl- and heteroarylsulfonylamide moieties were successfully obtained using 4-aminobenzenesulfonamides as starting compounds. To further expand the scope of the reaction, acid hydrazides were employed instead of amines under the same conditions. The reaction proceeded smoothly to give corresponding bis(hydrazides) **7m,n** in 72–76% yield. Finally, phenylenediamines were used as reagents to give compounds **7o–q** with two 7-azacoumrin moieties in excellent yields (91–97%). Similarly, 3,3′- and 4,4′-sulfonyldianilines furnished bis(7-azacoumarins) **7r,s** in a 67–78% yield. The structures of compounds **7a,f** were additionally confirmed by X-ray analysis (see Supplementary Information, Table S1, Figures S4 and S5 for detailed X-ray data).

The evaluation of the cytotoxicity of acids **3a,b** was also of importance for comparison purposes. Additionally, we synthesized a series of ammonium salts **8a–f** (Scheme 3, see Supplementary Information, Figures S56–S69 for the NMR spectra of the obtained compounds). Not only would this allow us to evaluate the effect of counterion on the cytotoxicity of acids **3**, but also to estimate the importance of covalent amide bonds for anticancer activity. The structure of the salt **8b** was additionally proved by X-ray analysis, which confirmed its ionic character (see Supplementary Information, Table S1, Figures S6 and S7 for detailed X-ray data).

### 2.2. Biological Studies

With a series of 7-azacoumarin-3-carboxamides **7** in hand, we tested their cytotoxicity against normal (Chang liver) and tumor (M-HeLa, HuTu 80) cell lines at concentrations of 1–100 µM (Table 2). In general, the cytotoxicity of all the tested compounds was somewhat higher towards the HuTu 80 cell line than M-Hela cells. The cytotoxicity of carboxylic acids **3a,b** and their ammonium salts **8b,e** appeared to be close to each other and considerably lower than the cytotoxicity of reference compounds 5-fluorouracil and Doxorubicin. Carboxamides **7h–l** and **7r,s** possessing sulfonylamide moiety appeared to be more cytotoxic compared to others. The sulfonylamide **7h** and bis(7-azacoumarin-3-carboxamides) **7o,r,s** were the most cytotoxic towards the HuTu 80 cells. The cytotoxicity of these compounds was comparable to that of Doxorubicin (IC_50_ = 2.9–5.5 µM vs. 3.0 ± 0.2 µM), except compound **7s** (IC_50_ = 13.8 µM). However, in sharp contrast to Doxorubicin, their cytotoxicity towards the normal cell line was much lower (selectivity index, SI = IC_50_(Chang liver)/IC_50_(HuTu 80) = 3.8–14 vs. SI = 1 for Doxorubicin). Notably, bis(carboxamide) **7o** was the least selective (SI = 3.8), despite being one of the most active. The sulfonylamides **7h,r,s** exhibited much higher selectivity (SI = 9–14). Thus, one can speculate that the sulfonylamide moiety is crucial for both the activity and the selectivity of carboxamides **7h,r,s**. Taking into account both the selectivity and cytotoxicity towards HuTu 80 cells, the compounds **7h** and **7r** can be considered the most potent and promising.

**Figure 1:**
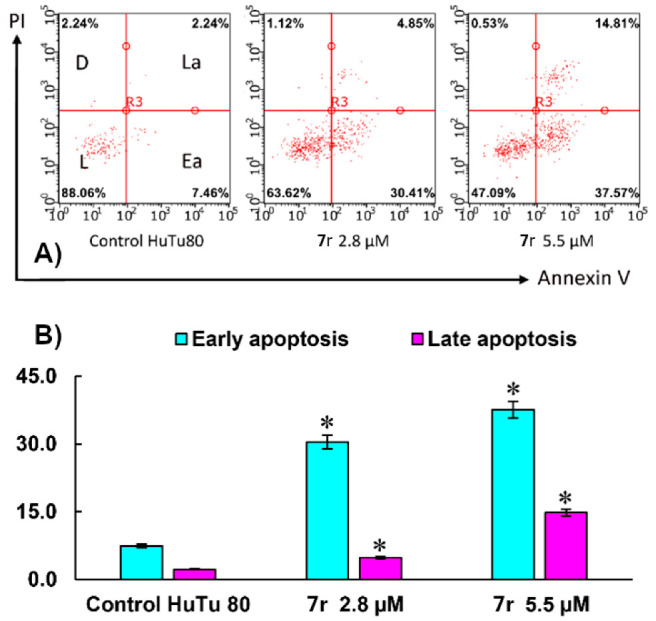
(**A**) Induction of apoptosis in HuTu 80 cells incubated with compound **7r** at concentration IC_50_/2 (2.8 µM) and at concentration IC_50_ (5.5 µM) in HuTu 80 cells; L—living cells; D—dead cells; Ea.—early apoptotic cells; La.—late apoptotic cells; (**B**) Percentage of early apoptotic cells (cyan) and late apoptotic cells (magenta) in control HuTu 80 cells and HuTu 80 cells treated with compound **7r**. Data are presented as mean ± SD of three independent experiments. * Values indicate *p* < 0.05 compared to control.

Induction of apoptosis is one of the most important mechanisms of anticancer activity. The apoptosis-inducing effect in HuTu 80 cells was studied by flow cytometry using sets of annexin V binding to the apoptosis marker phosphatidylserine. On the surface of the membranes of healthy cells, phosphatidylserine is contained in a minimal amount. Therefore, the interaction of annexin V with these cells is negligible. During apoptosis, phosphatidylserine molecules appear on the cell surface and can interact with the protein. This interaction leads to an increase in the fluorescence intensity of apoptotic cells, which is recorded by a flow cytometer. The apoptosis-inducing effect was evaluated using the example of the leader compound **7r** at IC50/2 and IC50 concentrations on the HuTu 80 cell line (Figure 1). After 24-h incubation in the presence of **7r**, apoptotic effects were registered in human duodenal adenocarcinoma cells, which were more pronounced at the early stage of apoptosis.

**Figure 2:**
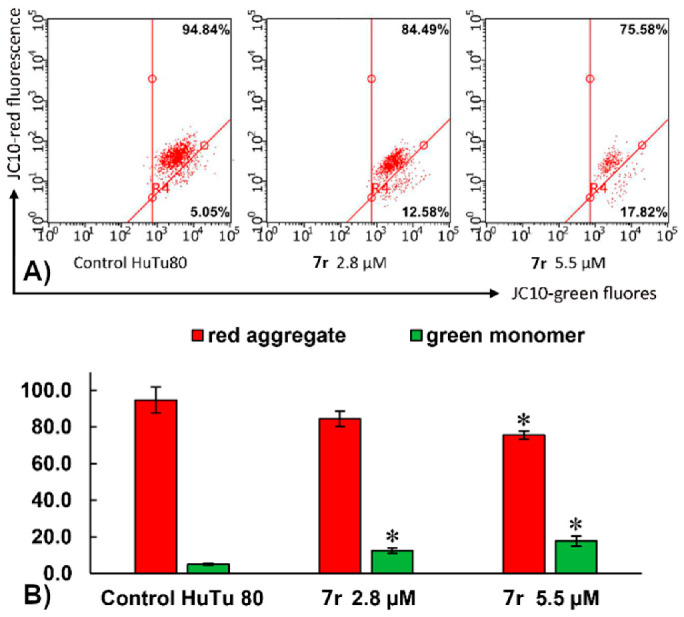
(**A**) Effects on the mitochondrial membrane potential by compound **7r** at concentration IC_50_/2 (2.8 µM) and at concentration IC_50_ (5.5 µM); (**B**) percentage of J-aggregates (red) and J-monomers (green) in control HuTu 80 cells and HuTu 80 cells treated with compound **7r**. Data are presented as mean ± SD of three independent experiments. * Values indicate *p* < 0.05 compared to control.

Violation of the functions of the mitochondria of the cell is one of the most common signs of apoptosis inherent in eukaryotic organisms [39]. With mitochondrial dysfunction, pro-apoptotic factors are released into the cytoplasm—cytochrome c, AIF, Smac/DIABLO, endonuclease G, as well as proforms of caspases 2, 3, and 9—inducing the cascade [40]. The release of these protein factors can be associated both with the rupture of mitochondrial membranes and with the activation of specific channels in the outer mitochondrial membrane. This usually leads to a change in the mitochondrial membrane potential (ΔΨm) due to a change in the permeability of the inner mitochondrial membrane for H+ protons. Methods for studying the membrane potential of mitochondria using flow cytometry are based on the use of cationic lipophilic fluorescent dyes. The principle of operation of these dyes is determined by their ability to spontaneously penetrate through the cytoplasmic membranes of cells, as well as the outer and inner membranes of mitochondria and accumulate in areas with a high concentration of protons, that is, under the inner mitochondrial membrane. This effect is accompanied by a change in the intensity of cell fluorescence, which is recorded by cytofluorimetry. In this study, the fluorescent dye JC-10 from the Mitochondria Membrane Potential Kit was used to evaluate the change in ΔΨm under the action of compound **7r** at IC_50_/2 and IC_50_ concentrations on the HuTu 80 cell line. JC-10 accumulates in the mitochondrial matrix and forms aggregates (J-aggregates) with red fluorescence in normal cells with a high mitochondrial membrane potential. Membrane mitochondrial potential is reduced in apoptotic cells. In this case, JC-10 begins to diffuse out of the mitochondria and turns into a monomeric form (J-monomer), and emits green fluorescence. A decrease in the mitochondrial membrane potential of HuTu 80 cells was observed after 24-h incubation with the leader compound **7r**. The process of depolarization of the mitochondrial membrane increased with increasing compound concentrations up to IC_50_ (Figure 2). The results obtained suggest that the mechanism of the cytotoxic action of **7r** may be associated with the induction of apoptosis via the intrinsic mitochondrial pathway.

**Figure 3:**
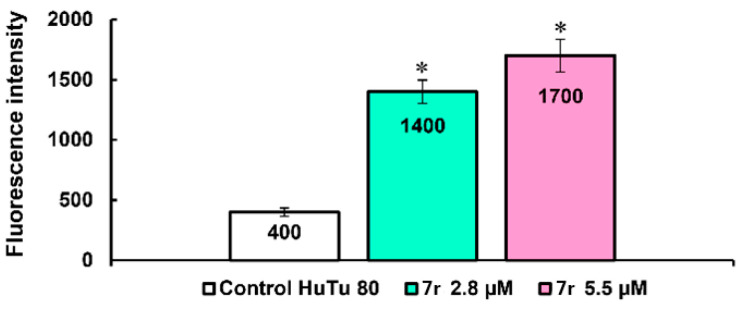
Induction of ROS production by compound **7r** at concentration IC_50_/2 (2.8 µM) and at concentration IC_50_ (5.5 µM). Data are presented as mean ± SD of three independent experiments. * Values indicate *p* < 0.05 compared to control.

Increased generation of reactive oxygen species (ROS) by the test compounds can also characterize the development of apoptosis along the mitochondrial pathway. Mitochondria are both a potential source and target of ROS. An increase in ROS production leads to the disruption of mitochondrial functions and, subsequently, to irreversible damage and cell death. In this regard, the effect of lead compound **7r** at IC50/2 and IC50 concentrations on ROS production in HuTu 80 cells was investigated using a flow cytometry assay and the CellROX^®^ Deep Red flow cytometry kit. The data presented in Figure 3 show a significant increase in CellROX^®^ Deep Red fluorescence intensity when **7r** is added at any concentration compared to the control (unstained cells). This indicates an increase in ROS production in the presence of the test compound.

## 3. Materials and Methods

### 3.1. Chemistry

#### 3.1.1. General Methods

^1^H, ^13^C, and ^31^P NMR spectra were recorded on a Bruker Avance 400 (Bruker, Billerica, MA, USA) spectrometer (at the frequencies of 400.05, 100.61, and 161.94 MHz, respectively) and on a Bruker Avance 600 spectrometer (at the frequencies of 600.1, 150.9, and 242.0 MHz, respectively). Values of the chemical shifts for the ^1^H and ^13^C nuclei are reported relative to the residual signals of the solvent (DMSO-d_6_), and those for the ^31^P nuclei are given relative to the used standard (H_3_PO_4_, dP = 0.00). IR spectra were recorded in KBr pellets on a Bruker 3/5 E2_76513 Tensor-27 spectrometer in the range of 400–3600 cm^−1^. Mass spectra were recorded on an Ultraflex III TOF/TOF Bruker instrument (*p*-nitroaniline as the matrix) and an AmaZon X Bruker instrument. Elemental analysis was performed using a Carlo Erba EA 1108 (Carlo Erba, Cornaredo, Italy) instrument. Commercially available pyridoxal hydrochloride (abcr Gute Chemie, Karlsruhe, Germany), pyridoxal 5′-phosphate monohydrate (Sigma-Aldrich, St. Louis, MO, USA), Meldrum’s acid (TCI, Tokyo, Japan), amines and diamines (Acros organics, Geel, Belgium) were used in the synthesis without additional purification.

#### 3.1.2. Procedures for the Synthesis of Compounds **3–6**

**5-(hydroxymethyl)-8-methyl-2-oxo-2*H*-pyrano[2,3-c]pyridin-7-ium-3-carboxylate 3ª.** To a solution of pyridoxal **1** (0.82 g, 4.91 mmol) or pyridoxal hydrochloride **1·HCl** (1 g, 4.91 mmol) in distilled water (10 mL), Meldrum’s acid (0.707 g, 4.91 mmol) was added. The reaction mixture was stirred at room temperature for 12 h. The precipitate was filtered off, washed consequently with dry ethanol (2 × 5 mL) and diethyl ether (10 mL), and dried in a vacuum to give compound **3a** as a white solid. Yield: 0.79 g (69%); mp: 249–250 °C. IR (ν cm^−1^): 646, 733, 810, 908, 975, 1006, 1038, 1088, 1153, 1253, 1293, 1360, 1409, 1772, 3450. ^1^H NMR (DMSO-d_6_) δ ppm: 2.57 (s, 3H, CH_3_); 4.77 (s, 2H, CH_2_OH), 8.36 (s, 1H, CH_arom_) 8.73 (s, 1H, CH). ^13^C NMR (DMSO-d_6_) δ ppm: 18.98, 58.54, 121.54, 123.77, 132.25, 143.10, 143.43, 146.94, 148.13, 155.80, 164.18. MALDI TOF—MS *m*/*z*: 235.1 [M]^+^. Calculated for C_11_H_9_NO_5_, %: C, 56.18; H, 3.86; N, 5.96. Found, %: C,56.27; H, 3.75; N, 5.83.

**8-methyl-2-oxo-5-((phosphonooxy)methyl)-2*H*-pyrano[2,3-c]pyridin-7-ium-3-carboxylate 3b.** A suspension of pyridoxal-5′-phosphate monohydrate (0.54 g, 2.04 mmol) and Meldrum’s acid (0.29 g, 2.04 mmol) in distilled water (5 mL) was stirred for 4 h at room temperature. The formed precipitate was filtered off, washed with absolute ethanol (2 × 5 mL), then with diethyl ether (10 mL), and dried in a vacuum to give the compound **3b** as a light yellow solid. Yield: 0.55 g (86%); mp: >300 °C. IR (ν cm^−1^): 726, 806, 823, 962, 1025, 1074, 1095, 1137, 1161, 1220, 1239, 1297, 1425, 1751, 1770, 2418, 3508, 3548. ^1^H NMR (DMSO-d_6_) δ ppm: 2.60 (s, 3H, CH_3_), 5.19 (d, 2H, *J* = 7.7 Hz, CH_2_O), 8.42 (s, 1H, CH_arom_), 8.65 (s, 1H, CH). ^13^C NMR (DMSO-d_6_) δ ppm: 19.06, 62.04, 121.67, 124.56, 127.56, 142.43, 144.12, 147.89, 148.27, 155.63, 164.07. ^31^P NMR (DMSO-d_6_) δ ppm: −1.88. MALDI TOF—MS *m*/*z*: 315.9 [M+H]^+^. Calculated for C_11_H_10_NO_8_P, %: C,41.92; H, 3.20; N, 4.44; P, 9.83. Found, %: C,41.57; H, 3.25; N, 4.53; P, 9.48.

**5-(2-hydroxy-4,4-dimethyl-6-oxocyclohex-1-en-1-yl)-4-(hydroxymethyl)-1,8,8-trimethyl-5,7,8,9-tetrahydro-6*H*-chromeno[2,3-c]pyridin-6-one 4.** A solution of pyridoxal (0.5 g, 2.99 mmol) and dimedone (0.84 g, 5.99 mol) was stirred at 60 °C for 4 h. The precipitate was filtered off. The filtrate was evaporated; the residue was refluxed in ethanol: diethyl ether mixture (1:4, 5 mL). The undissolved precipitate was filtered off. The precipitates were merged to give 1.1 g (89%) of target compound **4** as a light yellow solid; mp: 219–224 °C. IR (ν cm^−1^): 655, 918, 938, 974, 1005, 1043, 1064, 1130, 1150, 1171, 1199, 1220, 1234, 1267, 1368, 1407, 1604, 1643, 2956, 3100. ^1^H NMR (DMSO-d_6_) δ ppm: 0.87 (s, 6H, 2CH_3_), 0.93 (s, 3H, CH_3_), 1.06 (s, 3H, CH_3_), 2.03 (d, 1H, *J* = 16.0 Hz, CH_2_), 2.11 (br.s, 4H, 2CH_2_), 2.26 (d, 1H, *J* = 15.9 Hz, CH_2_), 2.38 (d, 1H, *J* = 15.2 Hz, CH_2_), 2.40 (s, 3H, CH_3_), 2.60 (d, 1H, *J* = 17.3 Hz, CH_2_), 4.23 (d, 1H, *J* = 14.5 Hz, CH_2_OH), 4.51 (d, 1H, *J* = 14.5 Hz, CH_2_OH), 5.00 (s, 1H, CH), 8.14 (s, 1H, CH_arom_). ^13^C NMR (DMSO-d_6_) δ ppm: 19.08, 23.45, 26.28, 28.11, 29.69, 31.84, 32.07, 41.06, 50.99, 58.23, 110.59, 129.58, 134.12, 142.40, 143.50, 145.63, 164.98, 196.01. MALDI TOF—MS *m*/*z*: 412.4 [M+H]^+^. Calculated for C_24_H_29_NO_5_, %: C, 70.05; H, 7.10; N, 3.40. Found, %: C, 70.17; H, 7.15; N, 3.43.

**1-(2-hydroxy-5-(hydroxymethyl)-2,8-dimethyl-2*H*-pyrano[2,3-c]pyridin-3-yl)ethan-1-one, compound 5.** To a suspension of pyridoxal (0.6 g, 3.59 mmol) in dry ethanol (4 mL), acetylacetone (0.5 g, 5 mmol) was added, followed by piperidine (0.006 g, 0.07 mmol) and acetic acid (0.004 g, 0.07 mmol). The reaction mixture was refluxed for 2 h, and then kept at room temperature for an additional 12 h. The precipitate of compound **5** was filtered off. The evaporation of the filtrate and recrystallization of the viscous residue from absolute ethanol (3 mL) provided an additional sample of compound **5**. Light yellow solid. Yield: 0.7 g (79%); mp: 174 °C. IR (ν cm^−1^): 563, 606, 664, 747, 857, 885, 901, 923, 949, 967, 983, 1034, 1066, 1088, 1099, 1150, 1185, 1211, 1242, 1293, 1338, 1370, 1386, 1410, 1458, 1484, 1630, 1678, 2749, 2922, 3030, 3268. ^1^H NMR (DMSO-d_6_) δ ppm: 1.86 (s, 3H, CH_3_), 2.39 (s, 3H, CH_3_), 2.46 (s, 3H, CH_3_), 4.67 (dd, 2H, *J* = 5.6, 2.3 Hz, CH_2_OH), 5.35 (t, 1H, *J* = 5.5 Hz, CH_2_OH), 7.17 (s, 1H, OH), 7.72 (s, 1H, CH_arom_), 8.05 (c, 1H, CH). ^13^C NMR (DMSO-d_6_) δ ppm: 19.40, 27.91, 28.72, 59.17, 98.33, 123.77, 129.30, 131.92, 138.66, 140.93, 146.74, 147.32, 197.85. MALDI TOF—MS *m*/*z*: 250.2 [M+H]^+^. Calculated for C_13_H_15_NO_4_, %: C, 62.64; H, 6.07; N, 5.62. Found, %: C, 62.57; H, 6.15; N, 5.53.

**1,3-bis(7-hydroxy-6-methyl-1,3-dihydrofuro[3,4-c]pyridin-1-yl)propan-2-one 6.** To a solution of acetylacetone (0.19 g, 1.9 mmol) in a mixture of ethanol (30 mL) and 40% aqueous solution of KOH (10 mL) pyridoxal (0.64 g, 3.8 mmol) was added. The reaction mixture was stirred at room temperature for 24 h. Then, concentrated hydrochloric acid was added to the resulting solution (pH = 7), and the precipitated potassium chloride was filtered off. The filtrate was evaporated, and pure compound **6** was isolated by recrystallization from anhydrous ethanol. Light orange solid. Yield: 0.56 g (82%); mp: 157–160 °C. IR (ν cm^−1^): 516, 570, 652, 778, 840, 985, 1048, 1199, 1237, 1312, 1386, 1423, 1533, 1617, 1712, 2861, 3100, 3255, 3392. ^1^H NMR (DMSO-d_6_) δ ppm: 2.37 (s, 6H; 2CH_3_), 2.77 (dd, 2H, *J* = 16.4, 9.3 Hz, CH_2_), 3.06 (dd, 2H, *J* = 16.5, 2.5 Hz, CH_2_), 4.92 (d, 2H, *J* = 12.4 Hz, CH_2_O), 5.01 (dd, 2H, *J* = 12.4, 2.5 Hz, CH_2_O,), 5.68 (d, 2H, *J =* 9.2 Hz, CH), 7.89 (s, 2H, 2CH_arom_). ^13^C NMR (DMSO-d_6_) δ ppm: 19.89, 47.63, 70.92, 78.71, 133.22, 135.62, 136.36, 146.25, 146.62, 206.32. MALDI TOF—MS *m*/*z*: 357.2 [M+H]^+^. Calculated for C_19_H_20_N_2_O_5_, %: C 64.04; H 5.66; N 7.86. Found, %: C 63.97; H 5.63; N 4.91.

#### 3.1.3. General Procedure for the Synthesis of Compounds **7**

The mixture of the acid **3a** (0.3 g, 1.28 mmol), the corresponding amine (2.56 mmol, 1.28 mmol in case of diamines), and 1.25 mL of phosphorus oxychloride was heated at 90 °C for 1 h. With heating, the precipitate gradually dissolved, and the reaction mixture acquired a dark purple color. Upon completion of the heating, the reaction mixture was cooled, and a small amount of ice was added. Then, with cooling and stirring, 10% aqueous ammonia solution was added dropwise until pH = 6–7. The formed precipitate was filtered off and washed consequently with hot water (2 × 5 mL), ethyl alcohol (2 × 5 mL), and diethyl ether (5 mL). If additional purification was required, the precipitate was refluxed in 10 mL of water and then in 10 mL of ethanol and filtered off. The resulting precipitate was dried in a vacuum to give title compounds **7**.

#### 3.1.4. Characterization Data for Compounds **7**

5-(Chloromethyl)-8-methyl-2-oxo-*N*-phenyl-2a*H*-pyrano[2,3-c]pyridine-3-carboxamide **7a**

Yellow solid. Yield: 90%; mp: 240 °C dec. IR (ν cm^−1^): 629, 703, 762, 791, 1054, 1214, 1255, 1285, 1412, 1444, 1491, 1550, 1595, 1616, 1666, 1723, 3242. ^1^H NMR (DMSO-d_6_) δ ppm: 2.66 (s, 3H, CH_3_), 5.24 (s, 2H CH_2_Cl), 7.17 (t, 1H, *J* = 7.4 Hz, Ph), 7.41 (t, 2H, *J* = 7.7 Hz, Ph), 7.73 (d, 2H, *J* = 8.0 Hz, Ph), 8.57 (s, 1H, CH_arom_), 8.87 (s, 1H, CH), 10.62 (s, 1H, NH). ^13^C NMR (DMSO-d_6_) δ ppm: 19.21, 40.19, 120.35, 122.08, 125.00, 126.77, 128.55, 129.53, 138.32, 140.87, 144.88, 147.69, 148.82, 158.76,160.01. MALDI TOF—MS *m*/*z*: 329.4 [M]^+^. Calculated for C_17_H_13_ClN_2_O_3_, %: C,62.11; H, 3.99; Cl, 10.78; N, 8.52. Found, %: C, 62.09; H, 4.01; Cl, 10.63; N, 8.87.

5-(Chloromethyl)-*N*-(2-methoxyphenyl)-8-methyl-2-oxo-2*H*-pyrano[2,3-c]pyridine-3-carboxamide **7b**

Orange solid. Yield: 91%; mp: 214–5 °C. IR (ν cm^−1^): 717, 751, 788, 885, 972, 1022, 1114, 1145, 1229, 1256, 1284, 1305, 1389, 1435, 1467, 1486, 1541, 1598, 1622, 1670, 1732, 3233. ^1^H NMR (DMSO-d_6_) δ ppm: 2.66 (s, 3H, CH_3_), 3.93 (s, 3H, CH_3_), 5.26 (s, 2H, CH_2_Cl), 7.00 (ddd, 1H, *J* = 8.5, 5.9, 3.0 Hz, Ph), 7.07–7.15 (m, 2H, Ph), 8.42–8.46 (m, 1H, Ph), 8.59 (s, 1H, CH_arom_), 9.05 (s, 1H, CH), 11.14 (s, 1H, NH). ^13^C NMR (DMSO-d_6_) δ ppm: 18.74, 39.65, 56.59, 111.55, 120.03, 121.15, 122.69, 124.57, 125.27, 127.47, 128.95, 142.33, 143.92, 147,94, 148.60, 149.00, 158.49, 159.84. MALDI TOF—MS *m*/*z*: 359.1 [M]^+^. Calculated for C_18_H_15_ClN_2_O_4_, %: C, 60.26; H, 4.21; Cl, 9.88; N, 7.81. Found, %: C, 60.22; H, 4.15; Cl, 9.53; N, 7.99.

*N*-(2-Bromo-4-nitrophenyl)-5-(chloromethyl)-8-methyl-2-oxo-2*H*-pyrano[2,3-c]pyridine-3-carboxamide **7c**

Light orange solid. Yield: 77%; mp: 240 °C dec. IR (ν cm^−1^): 690, 714, 742, 764, 793, 837, 891, 1057, 1118, 1159, 1215, 1268, 1313, 1341, 1395, 1413, 1514, 1547, 1584, 1613, 1677, 1727, 3167, 3391, 3486. ^1^H NMR (DMSO-d_6_) δ ppm: 2.66 (s, 3H, CH_3_), 5.29 (s, 2H, CH_2_Cl), 8.35 (dd, 1H, *J* = 9.2, 2.7 Hz, Ph), 8.57 (d, *J* = 2.6 Hz, Ph), 8.61 (s, 1H, CH_arom_), 8.76 (d, 1H, *J* = 9.2 Hz, Ph), 9.13 (s, 1H, CH), 11.47 (s, 1H, NH). ^13^C NMR (DMSO-d_6_) δ ppm:19.21, 40.19, 113.54, 113.94, 121.67, 122.02, 123.60, 124.83, 125.57, 128.62, 128.79, 129.34, 142.11, 143.88, 143.94, 145.01, 147.86, 149.14, 160.12, 160.15. Calculated for C_17_H_11_BrClN_3_O_5_, %: C, 45.11; H, 2.45; Br, 17.65; Cl, 7.83; N, 9.28. Found, %: C, 44.95; H, 2.63; Br, 17.38; Cl, 7.95; N, 9.13.

5-(Chloromethyl)-8-methyl-2-oxo-*N*-(pyridin-2-yl)-2*H*-pyrano[2,3-c]pyridine-3-carboxamide **7d**

Black solid.Yield: 79%; mp: >300 °C. IR (ν cm^−1^): 700, 764, 793, 972, 990, 1055, 1148, 1220, 1265, 1293, 1305, 1378, 1415, 1435, 1534, 1575, 1591, 1671, 1723, 3282, 3435. ^1^H NMR (DMSO-d_6_) δ ppm: 2.66 (s, 3H, CH_3_), 5.25 (s, 2H, CH_2_Cl), 7.22 (dd, 1H, *J* = 7.2, 5.1 Hz, Py), 7.91 (t, 1H, *J* = 7.9 Hz, Py), 8.25 (d, 1H, *J* = 8.1 Hz, Py), 8.40 (d, 1H, *J* = 4.0 Hz, Py), 8.58 (s, 1H, CH_arom_), 9.00 (s, 1H, CH), 11.07 (s, 1H, NH). ^13^C NMR (DMSO-d_6_) δ ppm: 19.23, 40.19, 114.24, 121.08, 122.02, 128.61, 129.76, 132.08, 139.21, 144.89, 148.95, 151.17, 159.83. Calculated for C_16_H_12_ClN_3_O_3_, %: C, 58.28; H, 3.67; Cl, 10.75; N, 12.74. Found, %: C, 58.18; H, 3.81; Cl, 10.65; N, 13.01. 

*N*-(2-Aminopyridin-3-yl)-5-(chloromethyl)-8-methyl-2-oxo-2*H*-pyrano[2,3-c]pyridine-3-carboxamide **7e**

Dark brown solid. Yield: 66%; mp: >300 °C. IR (ν cm^−1^): 701, 762, 789, 889, 946, 1055, 1149, 1228, 1294, 1376, 1414, 1450, 1549, 1592, 1626,1666, 1733, 3233, 3350, 3424. ^1^H NMR (DMSO-d_6_) δ ppm: 2.66 (s, 3H, CH_3_), 5.25 (s, 2H, CH_2_Cl), 5.98 (br.s, 2H, NH_2_), 6.68 (dd, 1H, *J* = 7.7, 5.0 Hz, Py), 7.77 (dd, 1H, *J* = 7.6, 1.7 Hz, Py), 7.89 (dd, 1H, *J* = 5.0, 1.7 Hz, Py), 8.58 (s, 1H, CH_arom_), 8.91 (s, 1H, CH), 9.95 (s, 1H, NH). ^13^C NMR (DMSO-d_6_) δ ppm: 19.75, 41.12, 113.36, 118.78, 122.53, 126.62, 129.13, 133.52, 141.62, 145.43, 145.60, 148.22, 149.40, 154.50, 159.44, 161.07. MALDI TOF—MS *m*/*z*: 345.1 [M]^+^. Calculated for C_16_H_13_ClN_4_O_3_, %: C, 55.74; H, 3.80; Cl, 10.28; N, 16.25. Found, %: C, 55.89; H, 3.97; Cl, 10.43; N, 16.25. 

5-(Chloromethyl)-3-(diphenylcarbamoyl)-8-methyl-2-oxo-2*H*-pyrano[2,3-c]pyridin-7-ium chloride **7f**

Yellow solid. Yield: 78%; mp: 201–5 °C. IR (ν cm^−1^): 695, 766, 799, 1055, 1079, 1150, 1202, 1263, 1280, 1309, 1352, 1407, 1453, 1491, 1550, 1592, 1651, 1723, 3429. ^1^H NMR (DMSO-d_6_) δ ppm: 2.52 (s, 3H, CH3), 5.08 (s, 2H, CH_2_Cl), 7.23–7.41 (m, 10H, 2Ph), 8.46 (s, 1H, CH_arom_), 8.61 (s, 1H, CH). ^13^C NMR (DMSO-d_6_) δ ppm: 19.03, 40.06, 121.54, 127.01, 127.46, 128.26, 128.56, 129.07, 129.73, 130.00, 130.34, 138.41, 142.09, 144.79, 147.16, 148.65, 156.16, 163.65. MALDI TOF—MS *m*/*z*: 427.1[M-Cl+Na]^+^. Calculated for C_23_H_18_Cl_2_N_2_O_3_, %: C, 62.60; H, 4.11; Cl, 16.07; N, 6.35. Found, %: C, 62.42; H, 4.01; Cl, 15.93; N, 6.14.

5-(Chloromethyl)-8-methyl-2-oxo-*N*-(prop-2-yn-1-yl)-2*H*-pyrano[2,3-c]pyridine-3-carboxamide **7g**

Brown solid. Yield: 45%; mp: 161 °C dec. IR (ν cm^−1^): 546, 562, 657, 699, 799, 957, 978, 1069, 1157, 1230, 1266, 1293, 1343, 1419, 1477, 1513, 1544, 1595, 1611, 1666, 1715, 2122, 2937, 3055, 3291, 3314. ^1^H NMR (DMSO-d_6_) δ ppm: 2.62 (s, 3H, CH_3_), 3.16 (t, 1H, *J* = 2.5 Hz, CH), 4.14 (dd, 2H, *J* = 5.7, 2.5 Hz, CH_2_), 5.21 (s, 2H, CH_2_Cl), 8.54 (s, 1H, CH_arom_), 8.86 (s, 1H, CH), 8.98 (t, 1H, *J* = 5.7 Hz, NH). ^13^C NMR (DMSO-d_6_) δ ppm: 19.76, 29.91, 41.13, 74.36, 81.50, 122.52, 125.23, 129.05, 142.46, 145.20, 148.37, 149.39, 159.35, 161.48. MALDI TOF—MS *m*/*z*: 291.1 [M]^+^. Calculated for C_14_H_11_ClN_2_O_3_, %: C, 57.84; H, 3.81; Cl, 12.19; N, 9.64. Found, %: C, 57.96; H, 4.10; Cl, 12.13; N, 9.58.

5-(Chloromethyl)-8-methyl-2-oxo-*N*-(4-sulfamoylphenyl)-2*H*-pyrano[2,3-c]pyridine-3-carboxamide **7h**

Peach solid. Yield: 65%; mp: >300 °C. IR (ν cm^−1^): 541, 552, 597, 682, 706, 763, 792, 837, 889, 1046, 1098, 1163, 1188, 1217, 1253, 1316, 1340, 1407, 1438, 1547, 1593, 1616, 1675, 1728, 3068, 3109, 3242, 3281, 3318. ^1^H NMR (DMSO-d_6_) δ ppm: 2.65 (s, 3H, CH_3_), 5.23 (s, 2H CH_2_Cl), 7.32 (s, 2H, NH), 7.87 (q, 4H, *J* = 8.8 Hz, Ph), 8.57 (s, 1H, CH_arom_), 8.87 (s, 1H, CH), 10.87 (s, 1H, NH). ^13^C NMR (DMSO-d_6_) δ ppm: 19.75, 41.07, 120.69, 122.52, 127.23, 127.92, 129.12, 140.63, 141.66, 145.46, 148.24, 149.40, 159.06, 161.16. ESI—MS *m*/*z*: 408.1 [M]^+^.Calculated for C_17_H_14_ClN_3_O_5_S, %: C, 50.07; H, 3.46; Cl, 8.69; N, 10.30; S, 7.86. Found, %: C, 50.09; H, 3.54; Cl, 8.63; N, 10.47; S, 7.75.

*N*-(4-(*N*-Acetylsulfamoyl)phenyl)-5-(chloromethyl)-8-methyl-2-oxo-2*H*-pyrano[2,3-c]pyridine-3-carboxamide **7i**

Coral solid. Yield: 72%; mp: >300 °C. IR (ν cm^−1^): 548, 576, 595, 623, 636, 685, 704, 762, 779, 791, 842, 862, 949, 996, 1055, 1091, 1159, 1187, 12,19, 1294, 1315, 1337, 1406, 1442, 1476, 1536, 1591, 1617, 1680, 1735, 3065, 3103, 3277. ^1^H NMR (DMSO-d_6_) δ ppm: 1.93 (s, 3H, CH_3_), 2.65 (s, 3H, CH_3_), 5.22 (s, 2H CH_2_Cl), 7.94 (s, 4H, Ph), 8.57 (s, 1H, CH_arom_), 8.86 (s, 1H, CH), 10.96 (s, 1H, NH), 12.03 (s, 1H, NH). ^13^C NMR (DMSO-d_6_) δ ppm: 19.73, 24.21, 41.05, 120.62, 122.49, 127.29, 129.14, 130.06, 135.32, 141.66, 143.28, 145.45, 148.24, 149.40, 158.91, 161.41, 169.73. ESI—MS *m*/*z*: 450.2 [M]^+^. Calculated for C_19_H_16_ClN_3_O_6_S, %: C, 50.73; H, 3.59; Cl, 7.88; N, 9.34; S, 7.13. Found, %: C, 50.69; H, 3.54; Cl, 7.73; N, 9.47; S, 7.25.

5-(Chloromethyl)-8-methyl-2-oxo-*N-*(4-(*N*-(pyrimidin-2-yl)sulfamoyl)phenyl)-2*H*-pyrano[2,3-c]pyridine-3-carboxamide **7j**

Rosy brown solid. Yield: 73%; mp: >300 °C. IR (ν cm^−1^): 569, 581, 623, 633, 682, 705, 763, 794, 840, 939, 1014, 1055, 1090, 1158, 1186, 1217, 1256, 1293, 1317, 1344, 1407, 1435, 1461, 1540, 15910, 1620, 1678, 1725, 3042, 3116, 3292, 3437. ^1^H NMR (DMSO-d_6_) δ ppm: 2.64 (s, 3H, CH_3_), 5.21 (s, 2H CH_2_Cl), 7.05 (t, 1H, *J* = 4.9 Hz, CH_Pyr_), 7.90 (d, 2H, *J* = 8.6 Hz, Ph), 8.02 (d, 2H, *J* = 8.6 Hz, Ph), 8.51 (d, 2H, *J* = 4.9 Hz, CH_Pyr_), 8.56 (s, 1H, CH_arom_), 8.84 (s, 1H, CH), 10.91 (s, 1H, NH). ^13^C NMR (DMSO-d_6_) δ ppm: 19.74, 41.05, 116.79, 120.42, 122.47, 127.38, 129.10, 130.04, 136.48, 141.51, 142.67, 145.47, 148.22, 149.39, 157.90, 158.89, 159.34, 161.34. ESI—MS *m*/*z*: 486.2 [M]^+^. Calculated for C_21_H_16_ClN_5_O_5_S, %: C, 51.91; H, 3.32; Cl, 7.30; N, 14.41; S, 6.60. Found, %: C, 51.99; H, 3.44; Cl, 7.27; N, 14.50; S, 6.47.

5-(Chloromethyl)-8-methyl-*N*-(4-(*N*-(4-methylpyrimidin-2-yl)sulfamoyl)phenyl)-2-oxo-2*H*-pyrano[2,3-c]pyridine-3-carboxamide **7k**

Rosy brown solid. Yield: 70%; mp: >300 °C. IR (ν cm^−1^): 546, 571, 582, 624, 681, 704, 762, 794, 841, 889, 964, 1054, 1092, 1158, 1186, 1217, 1257, 1318, 1345, 1405, 1439, 1498, 1543, 1591, 1679, 1727, 3064, 3287, 3393. ^1^H NMR (DMSO-d_6_) δ ppm: 2.33 (s, 3H, CH_3_), 2.64 (s, 3H, CH_3_), 5.21 (s, 2H CH_2_Cl), 6.90 (d, 1H, *J* = 5.2 Hz, CH_Pyr_), 7.89 (d, 2H, *J* = 8.9 Hz, Ph), 8.02 (d, 2H, *J* = 8.8 Hz, Ph), 8.32 (d, 1H, *J* = 5.1 Hz, CH_Pyr_), 8.56 (s, 1H, CH_arom_), 8.85 (s, 1H, CH), 10.90 (s, 1H, NH). ^13^C NMR (DMSO-d_6_) δ ppm: 19.73, 24.23, 41.06, 115.79, 120.48, 122.48, 127.33, 129.10, 130.24, 136.69, 141.54, 142.52, 145.46, 148.22, 149.39, 157.51, 158.93, 161.29. ESI—MS *m*/*z*: 500.2 [M]^+^. Calculated for C_22_H_18_ClN_5_O_5_S, %: C, 52.86; H, 3.62; Cl, 7.09; N, 14.01; S, 6.41. Found, %: C, 52.94; H, 3.51; Cl, 7.13; N, 14.11; S, 6.37.

5-(Chloromethyl)-*N*-(4-(*N*-(4,6-dimethylpyrimidin-2-yl)sulfamoyl)phenyl)-8-methyl-2-oxo-2*H*-pyrano[2,3-c]pyridine-3-carboxamide **7l**

Rosy brown solid. Yield: 66%; mp: >300 °C. IR (ν cm^−1^): 546, 562, 586, 624, 633, 680, 707, 763, 793, 840, 861, 974, 1015, 1057, 1083, 1159, 1185, 1217, 1256, 1315, 1350, 1384, 1404, 1416, 1498, 1543, 1594, 1678, 1729, 3066, 3110, 3247, 3286. ^1^H NMR (DMSO-d_6_) δ ppm: 2.26 (s, 6H, 2CH_3_), 2.64 (s, 3H, CH_3_), 5.21 (s, 2H CH_2_Cl), 6.75 (s, 1H, CH_Pyr_), 7.88 (d, 2H, *J* = 8.7 Hz, Ph), 8.02 (d, 2H, *J* = 8.5 Hz, Ph), 8.56 (s, 1H, CH_arom_), 8.85 (s, 1H, CH), 10.89 (s, 1H, NH). ^13^C NMR (DMSO-d_6_) δ ppm: 19.73, 23.82, 41.06, 120.06, 122.49, 127.31, 129.10, 130.43, 141.53, 142.32, 145.45, 148.22, 149.39, 157.15, 158.95, 161.24, 167.53. ESI—MS *m*/*z*: 514.2 [M]^+^. Calculated for C_23_H_20_ClN_5_O_5_S, %: C, 53.75; H, 3.92; Cl, 6.90; N, 13.63; S, 6.24. Found, %: C, 53.84; H, 3.98; Cl, 7.01; N, 13.74; S, 6.30.

*N*′-Benzoyl-5-(chloromethyl)-8-methyl-2-oxo-2*H*-pyrano[2,3-c]pyridine-3-carbohydrazide **7m**

Indianred solid. Yield: 75%; mp: 240 °C dec. IR (ν cm^−1^): 693, 715, 765, 929, 995, 1022, 1050, 1144, 1215, 1241, 1288, 1410, 1446, 1476, 1526, 1546, 1605, 1621, 1755, 3491. ^1^H NMR (DMSO-d_6_) δ ppm: 2.66 (s, 3H, CH_3_), 5.29 (s, 2H, CH_2_Cl), 7.68–7.71 (m, 3H, Ph), 8.14–8.17 (m, 2H, Ph), 8.58 (s, 1H, CH_arom_), 9.03 (s, 1H, CH). ^13^C NMR (DMSO-d_6_) δ ppm: 19.20, 40.22, 117.50, 121.68, 123.32, 127.41, 128.40, 130.06, 133.03, 140.12, 144.87, 147.81, 148.84, 154.83, 160.47, 165.25. MALDI TOF—MS *m*/*z*: 372.1[M]^+^. Calculated for C_18_H_14_ClN_3_O_4_, %: C, 58.15; H, 3.80; Cl, 9.54; N, 11.30. Found, %: C, 58.39; H, 3.71; Cl, 9.81; N, 10.99.

5-(Chloromethyl)-*N*′-isonicotinoyl-8-methyl-2-oxo-2*H*-pyrano[2,3-c]pyridine-3-carbohydrazide **7n**

Peach solid. Yield: 72%; mp: >300 °C. IR (ν cm^−1^): 701, 754, 790, 846, 1061, 1192, 1220, 1252, 1296, 1315, 1414, 1480, 1507, 1552, 1617, 1638, 1653, 1722, 3233. ^1^H NMR (DMSO-d_6_) δ ppm: 2.65 (s, 3H, CH_3_), 5.25 (s, 2H, CH_2_Cl), 7.84 (dd, 2H, *J* = 4.4, 1.7 Hz, Ph), 8.57 (s, 1H, CH_arom_), 8.80 (dd, 2H, *J* = 4.4, 1.7 Hz, Ph), 8.86 (s, 1H, CH), 10.67 (s, 1H, NH), 11.27 (s, 1H, NH). ^13^C NMR (DMSO-d_6_) δ ppm: 19.25, 39.73, 121.83, 121.91, 124.59, 128.52, 139.71, 142.18, 144.76, 147.91, 149.00, 150.90, 158,24, 160.21, 163.96. MALDI TOF—MS *m*/*z*: 373.1[M]^+^. Calculated for C_17_H_13_ClN_4_O_4_, %: C, 54.78; H, 3.52; Cl, 9.51; N, 15.03. Found, %: C, 54.65; H, 3.64; Cl, 9.33; N, 14.79.

*N*,*N*′-(1,2-Phenylene)bis(5-(chloromethyl)-8-methyl-2-oxo-2*H*-pyrano[2,3-c]pyridine-3-carboxamide) **7o**

Brown solid. Yield: 91%; mp: 239–241 °C. IR (ν cm^−1^): 704, 760, 792, 886, 957, 1015, 1054, 1083, 1146, 1220, 1266, 1292, 1412, 1456, 1479, 1518, 1547, 1600, 1676, 1722, 3233. ^1^H NMR (DMSO-d_6_) δ ppm: 2.64 (s, 6H, 2CH_3_), 5.25 (s, 4H, 2CH_2_Cl), 7.36 (dd, 2H, *J* = 6.1, 3.5 Hz, Ph), 7.89 (dd, 2H, *J* = 6.0, 3.5 Hz, Ph), 8.59 (s, 2H, 2CH_arom_), 9.02 (s, 2H, 2CH), 10.63 (s, 2H, 2NH). ^13^C NMR (DMSO-d_6_) δ ppm: 19.22, 40.13, 121.97, 125.03, 125.23, 126.84, 128.60, 130.98, 142.44, 144.88, 147.81, 148.99, 159.10, 160.23. MALDI TOF—MS *m*/*z*: 601.6 [M+Na-H]^+^. Calculated for C_28_H_20_Cl_2_N_4_O_6_, %: C, 58.05; H, 3.48; Cl, 12.24; N, 9.67. Found, %: C, 57.89; H, 3.59; Cl, 12.33; N, 9.82.

*N*,*N*′-(1,3-Phenylene)bis(5-(chloromethyl)-8-methyl-2-oxo-2*H*-pyrano[2,3-c]pyridine-3-carboxamide) **7p**

Brown solid. Yield: 97%; mp: 274 °C dec. IR (ν cm^−1^): 689, 793, 879, 952, 1055, 1082,1144, 1184, 1229, 1293, 1413, 1491, 1548, 1608, 1671, 1722, 3307, 3504. ^1^H NMR (DMSO-d_6_) δ ppm: 2.66 (s, 6H, 2CH_3_), 5.23 (s, 4H, 2CH_2_Cl), 7.40–7.44 (m, 1H, Ph), 7.52 (d, 2H, *J* = 8.0 Hz, Ph), 8.22 (s, 1H, Ph), 8.57 (s, 2H, 2CH_arom_), 8.87 (s, 2H, 2CH), 10.70 (s, 2H, 2NH). ^13^C NMR (DMSO-d_6_) δ ppm: 19.24, 40.20, 111.77, 116.44, 122.10, 126.97, 128.58, 130.18, 138.93, 140.82, 144.93, 147.72, 148.86, 158.70, 160.25. MALDI TOF—MS *m*/*z*: 602.0[M+Na]^+^. Calculated for C_28_H_22_Cl_2_N_4_O_6_, %: C, 57.84; H, 3.81; Cl, 12.19; N, 9.64. Found, %: C, 58.09; H, 3.61; Cl, 12.04; N, 9.79.

*N*,*N*′-(1,4-Phenylene)bis(5-(chloromethyl)-8-methyl-2-oxo-2*H*-pyrano[2,3-c]pyridine-3-carboxamide) **7q**

Brown solid. Yield: 95%; mp: >300 °C. IR (ν cm^−1^): 743, 792, 837, 951, 1055, 1083, 1150, 1217, 1259, 1292, 1314, 1411, 1516, 1561, 1616, 1721, 3294. ^1^H NMR (DMSO-d_6_) δ ppm: 2.66 (s, 6H, 2CH_3_), 5.24 (s, 4H, 2CH_2_Cl), 7.77 (s, 4H, Ph), 8.57 (s, 2H, 2CH_arom_), 8.87 (s, 2H, 2CH), 10.65 (s, 2H, 2NH). ^13^C NMR (DMSO-d_6_) δ ppm: 19.25, 40.21, 121.02, 122.12, 126.76, 128.58, 134.84, 140.88, 144.93. 147.72, 148.86, 158.79, 159.89. MALDI TOF—MS *m*/*z*: 602.0 [M+Na]^+^. Calculated for C_28_H_20_Cl_2_N_4_O_6_, %: C, 58.05; H, 3.48; Cl, 12.24; N, 9.67. Found, %: C, 58.12; H, 3.31; Cl, 12.13; N, 9.81.

*N*,*N*′-(Sulfonylbis(4,1-phenylene))bis(5-(chloromethyl)-8-methyl-2-oxo-2*H*-pyrano[2,3-c]pyridine-3-carboxamide) **7r**

Apricot solid. Yield: 78%; mp: >300 °C. IR (ν cm^−1^): 548, 570, 595, 623, 668, 690, 718, 762, 794, 839, 887, 953, 1013, 1055, 1084, 1106, 1150, 1184, 1217, 1256, 130,5 1352, 1404, 1438, 1537, 1590, 1618, 1678, 1728, 3061, 3115, 3286. ^1^H NMR (DMSO-d_6_) δ ppm: 2.63 (s, 6H, 2CH_3_), 5.21 (s, 4H, 2CH_2_Cl), 7.97 (q, 8H, *J* = 8.7 Hz, 2Ph), 8.55 (s, 2H, 2CH_arom_), 8.85 (s, 2H, 2CH), 10.97 (s, 2H, 2NH). ^13^C NMR (DMSO-d_6_) δ ppm: 19.72, 41.04, 121.21, 122.44, 127.17, 129.11, 129.71, 137.36, 141.73, 143.27, 145.46, 148.22, 149.39, 158.89, 161.40. ESI—MS *m*/*z*: 718.2 [M-H]^+^. Calculated for C_34_H_24_ClN_4_O_8_S, %: C, 56.75; H, 3.36; Cl, 9.85; N, 7.79; S, 4.46. Found, %: C, 56.91; H, 3.34; Cl, 9.90; N, 7.81; S, 4.52.

*N*,*N*′-(Sulfonylbis(3,1-phenylene))bis(5-(chloromethyl)-8-methyl-2-oxo-2*H*-pyrano[2,3-c]pyridine-3-carboxamide) **7s**

Rosewood solid. Yield: 67%; mp: >300 °C. IR (ν cm^−1^): 523, 563, 583, 613, 687, 716, 761, 794, 875, 894, 951, 970, 1015, 1054, 1084, 1101, 1152, 1217, 1304, 1413, 1479, 1546, 1592, 1616, 1673, 1723, 3063, 3240, 3285. ^1^H NMR (DMSO-d_6_) δ ppm: 2.64 (s, 6H, 2CH_3_), 5.21 (s, 4H, 2CH_2_Cl), 7.68 (t, 2H, *J* = 7.9 Hz, Ph), 7.75–7.77 (m, 2H, Ph), 7.90–7.93 (m, 2H, Ph), 8.49 (t, 2H, *J* = 2.0 Hz, Ph), 8.56 (s, 2H, 2CH_arom_), 8.87 (s, 2H, 2CH), 10.93 (s, 2H, 2NH). ^13^C NMR (DMSO-d_6_) δ ppm: 19.74, 41.03, 119.12, 122.46, 124.11, 125.84, 127.27, 129.12, 131.75, 139.98, 141.66, 142.59, 145.47, 148.22, 149.39, 158.75, 161.39. ESI—MS *m*/*z*: 741.2 [M-H+Na]^+^. Calculated for C_34_H_24_ClN_4_O_8_S, %: C, 56.75; H, 3.36; Cl, 9.85; N, 7.79; S, 4.46. Found, %: C, 56.87; H, 3.42; Cl, 9.98; N, 7.71; S, 4.32.

#### 3.1.5. General Procedure for the Synthesis of Compounds **8**

Method A. An aqueous solution of the acid 3a (0.3 g, 1.28 mmol) and the corresponding amine (1.28 mmol, 0.64 mmol in case of diamines) was stirred at room temperature for 2 h. The precipitate was filtered off and dried in a vacuum to give a title compound 8.

Method B. An aqueous solution of the acid 3a (0.3 g, 1.28 mmol) and the corresponding amine (1.28 mmol, 0.64 mmol in case of diamines) was stirred at room temperature for 2 h. The solvent was evaporated, and the residue was taken up in a ethanol:diethyl ether mixture (1:1, 4 mL). The precipitate was filtered off and dried in a vacuum to give a title compound **8**.

#### 3.1.6. Characterization Data for Compounds **8**

Pyridin-2-aminium 5-(hydroxymethyl)-8-methyl-2-oxo-2*H*-pyrano[2,3-c]pyridine-3-carboxylate **8a**

Method A. White solid. Yield: 96%; mp: 245 °C. IR (ν cm^−1^): 606, 623, 750, 773, 813, 843, 951, 984, 1017, 1072, 1148, 1208, 1256, 1370, 1413, 1491, 1603, 1637, 1666, 1746, 3089, 3325. ^1^H NMR (DMSO-d_6_) δ ppm: 2.56 (s, 3H, CH_3_), 4.76 (s, 2H, CH_2_OH), 6.53—6.61 (m, 2H, Py), 7.50 (t, 1H, *J* = 7.0 Hz, Py), 7.90 (d, 1H, *J* = 4.5 Hz, Py), 8.33 (s, 1H, CH_arom_), 8.58 (s, 1H, CH). ^13^C NMR (DMSO-d_6_) δ ppm: 18.98, 58.56, 109.94, 112.28, 121.87, 126.30, 132.04, 139.24, 141.23, 143.02, 144.93146.76, 147.94, 156.24, 158.94, 165.42. MALDI TOF—MS *m*/*z*: 235.4 [M-94 (C_5_H_6_N_2_—2-aminopyridine)]^+^. Calculated for C_16_H_15_N_3_O_5_, %: C, 58.36; H, 4.59; N, 12.76. Found, %: C, 58.15; H, 4.41; N, 12.69.

Cyclohexanaminium 5-(hydroxymethyl)-8-methyl-2-oxo-2*H*-pyrano[2,3-c]pyridine-3-carboxylate **8b**

Method A. White solid. Yield: 95%; mp: 213 °C. IR (ν cm^−1^): 569, 677, 746, 929, 1037, 1066, 1087, 1173, 1264, 1387, 1413, 1562, 1628, 1708, 2931, 3410. ^1^H NMR (DMSO-d_6_) δ ppm: 1.08–1.13 (m, 1H, C_6_H_11_), 1.21–1.32 (m, 4H, C_6_H_11_), 1.57 (d, 1H, *J* = 13.0 Hz, C_6_H_11_), 1.71 (d, 2H, *J* = 12.2 Hz, C_6_H_11_), 1.92 (d, 2H, *J* = 11.2 Hz, C_6_H_11_), 2.54 (s, 3H, CH3), 2.94 (td, 1H, *J* = 10.5, 10.0, 5.4 Hz, C_6_H_11_), 4.70 (s, 2H, CH_2_OH), 8.07 (s, 1H, CH_arom_), 8.25 (s, 1H, CH). ^13^C NMR (DMSO-d_6_) δ ppm: 18.96, 24.31, 25.11, 30.90, 49.67, 58.60, 123.00, 131.43, 142.70, 146.14, 147.35, 157.57, 165.87. MALDI TOF—MS *m*/*z*: 235.1 [M-99 (C_6_H_13_N—cyclohexylamine)]^+^. Calculated for C_17_H_22_N_2_O_5_, %: C, 61.07; H, 6.63; N, 8.38. Found, %: C, 60.76; H, 6.75; N, 8.13.

Pyridine-2,3-diaminium 5-(hydroxymethyl)-8-methyl-2-oxo-2*H*-pyrano[2,3-c]pyridine-3-carboxylate **8c**

Method A. Brown solid. Yield: 80%; mp: 184–5 °C. IR (ν cm^−1^): 645, 733, 772, 811, 844, 1014, 1038, 1086, 1151, 1254, 1290, 1375, 1410, 1577, 1681, 1750, 3191, 3321, 3449. ^1^H NMR (DMSO-d_6_) δ ppm: 2.56 (s, 6H, 2CH_3_), 4.76 (s, 4H, 2CH_2_OH), 6.65 (dd, 1H, *J* = 7.6, 5.9 Hz, Py), 6.99 (dd, 1H, *J* = 7.7, 1.4 Hz, Py), 7.26 (dd, 1H, *J* = 6.0, 1.4 Hz, Py), 8.32 (s, 2H, 2CH_arom_), 8.53 (s, 2H, 2CH). ^13^C NMR (DMSO-d_6_) δ ppm: 18.95, 58.53, 113.63, 119.63, 121.97, 124.39, 127.28, 131.92, 133.36, 140.31, 142.94, 146.65, 146.67, 147.82, 156.40, 165.92. MALDI TOF—MS *m*/*z*: 235.2 [M-109 (C_5_H_7_N_3_—2,3-diaminopyridine)]^+^. Calculated for C_27_H_25_N_5_O_10_, %: C, 55.96; H, 4.35; N, 12.08. Found, %: C, 55.31; H, 3.89; N, 12.63. 

Pyridine-2,6-diaminium 5-(hydroxymethyl)-8-methyl-2-oxo-2*H*-pyrano[2,3-c]pyridine-3-carboxylate **8d**

Method A. Palegreen solid. Yield: 95%; mp: 198 °C. IR (ν cm^−1^): 643, 733, 749, 777, 812, 908, 982, 1037, 1072, 1152, 1263, 1291, 1366, 1415, 1570, 1638, 1741, 3217, 3377, 3447. ^1^H NMR (DMSO-d_6_) δ ppm: 2.56 (s, 6H, 2CH_3_), 4.75 (s, 4H, 2CH_2_OH), 5.79 (d, 2H, *J* = 8.0 Hz, Py), 7.32 (t, 1H, *J* = 8.0 Hz, Py), 8.32 (s, 2H, 2CH_arom_), 8.52 (s, 2H, 2CH). ^13^C NMR (DMSO-d_6_) δ ppm: 18.99, 58.55, 95.47, 122.01, 127.34, 131.96, 140.25, 142.99, 143.08, 146.68, 147.85, 155.14, 156.45, 165.85. MALDI TOF—MS *m*/*z*: 236.0 [M-109 (C_5_H_7_N_3_—2,6-diaminopyridine)]^+^. Calculated for C_27_H_25_N_5_O_10_, %: C, 55.96; H, 4.35; N, 12.08. Found, %: C, 56.02; H, 3.94; N, 11.98. 

Pyridin-2-aminium 8-methyl-2-oxo-5-((phosphonooxy)methyl)-2*H*-pyrano[2,3-c]pyridine-3-carboxylate **8e**

Method B. White solid. Yield: 90%; mp: 203 °C. IR (ν cm^−1^): 495, 515, 562, 627, 732, 771, 804, 849, 963, 1052, 1161, 1243, 1299, 1442, 1488, 1636, 1668, 1751, 2745, 3089. ^1^H NMR (DMSO-d_6_) δ ppm: 2.56 (s, 3H, CH_3_), 5.09 (d, 2H, *J* = 7.5 Hz, CH_2_O), 6.67 (t, 1H, *J* = 6.5 Hz, Py), 6.76 (d, 1H, *J* = 8.7 Hz, Py), 7.69 (t, 1H, *J* = 7.8 Hz, Py), 7.82 (d, 1H, *J* = 6.0 Hz, Py), 8.36 (s, 1H, CH_arom_), 8.63 (s, 1H, CH). ^13^C NMR (DMSO-d_6_) δ ppm: 19.04, 61.50, 61.55, 112.15, 112.19, 121.93, 125.51, 128.35, 128.43, 139.8, 139.95, 141.68, 141.92, 141.96, 143.93, 147.70, 147.85, 155.87, 156.46, 156.49, 164.69. ^31^P NMR (DMSO-d_6_) δ ppm: -0.13. MALDI TOF—MS *m*/*z*: 315.3[M-94 (C_5_H_6_N_2_—2-aminopyridine)]^+^. Calculated for C_16_H_16_N_3_O_8_P, %: C, 46.95; H, 3.94; N, 10.27; P, 7.57. Found, %: C, 46.81; H, 4.16; N, 10.01; P, 7.51. 

Pyridine-2,6-diaminium 8-methyl-2-oxo-5-((phosphonooxy)methyl)-2*H*-pyrano [2,3-c]pyridine-3-carboxylate **8f**

Method B. White solid. Yield: 89%; mp: 215 °C. IR (ν cm^−1^): 477, 500, 565, 623, 726, 806, 953, 1029, 1073, 1096, 1162, 1219, 1297, 1415, 1549, 1652, 1771, 3089, 3348. ^1^H NMR (DMSO-d_6_) δ ppm: 2.57 (s, 6H, 2CH_3_), 5.11 (d, 4H, *J* = 7.7 Hz, 2CH_2_O), 5.82 (d, 2H, *J* = 8.2 Hz, Py), 7.42, (t, 1H, *J* = 8.2 Hz, Py), 8.38 (s, 2H, 2CH_arom_), 8.65 (s, 2H, 2CH). ^13^C NMR (DMSO-d_6_) δ ppm: 19.54, 62.14, 95.66, 122.36, 125.60, 128.76, 128.84, 142.55, 144.55, 145.41, 148.31, 148.40, 153.31, 156.33, 164.97. ^31^P NMR (DMSO-d_6_) δ ppm: 0.95. MALDI TOF—MS *m*/*z*: 236.0 [M-109 (C_5_H_7_N_3_—2,6-diaminopyridine)]^+^. Calculated for C_27_H_27_N_5_O_16_P_2_, %: C, 43.85; H, 3.86; N, 9.47; P, 8.38. Found, %: C, 43.30; H, 4.01; N, 10.20; P, 8.51. 

### 3.2. Biology

Cells and Materials. For the experiments, we used cancer cell cultures M-HeLa clone 11 (epithelioid carcinoma of the cervix, subline HeLa., clone M-HeLa), HuTu 80, human duodenal adenocarcinoma from the collection of the Institute of Cytology, Russian Academy of Sciences (St. Petersburg, Russia); human liver cells (Chang liver) from the collection and the Research Institute of Virology of the Russian Academy of Medical Sciences (Moscow, Russia).

Cytotoxic Assay. The cytotoxic effect on cells was determined using the colorimetric method of cell proliferation—the MTT test [34]. The cells were cultured in a standard Eagle’s nutrient medium manufactured at the Chumakov Institute of Poliomyelitis and Virus Encephalitis (PanEco company, Moscow, Russian Federation) and supplemented with 10% fetal calf serum and 1% nonessential amino acids. Cells were seeded on a 96-well Nunc plate at a concentration of 5 × 10^3^ cells per well in a volume of 100 μL of medium and cultured in a CO_2_ incubator at 37 °C until a monolayer was formed. Then the nutrient medium was removed, and 100 µL of solutions of the test drug in the given dilutions were added to the wells, which were prepared directly in the nutrient medium with the addition of 5% DMSO to improve solubility. After 24 h of incubation of the cells with the tested compounds, the nutrient medium was removed from the plates, and 100 µL of the nutrient medium without serum with MTT at a concentration of 0.5 mg/mL was added and incubated for 4 h at 37 °C. Formazan crystals were added at 100 µL of DMSO to each well. Optical density was recorded at 540 nm on an Invitrologic microplate reader (Russia). The experiments for all compounds were repeated three times.

The selectivity index was determined as a ratio of the half-maximal inhibitory concentration towards the normal cell line to the half-maximal inhibitory concentration towards the tumor cell line. E.g., for HuTu 80 cell line SI = IC_50_(Chang liver)/IC_50_(HuTu 80).

Flow Cytometry Assay

Cell Culture. HuTu 80 cells at 1 × 10^6^ cells/well in a final volume of 2 mL were seeded into six-well plates. After 24 h of incubation, solutions of compound 7r at concentrations IC_50_/2 (2.8 µM) and IC_50_ (5.5 µM) were added to wells. 

Cell Apoptosis Analysis. HuTu 80 cells were incubated with compound 7r at concentrations IC_50_/2 (2.8 µM) and IC_50_ (5.5 µM) for 24 h. The cells were harvested at 2000 rpm for 5 min and then washed twice with ice-cold PBS, followed by resuspension in binding buffer. Next, the samples were incubated with 5 μL of annexin V- Alexa Fluor 647 (Sigma-Aldrich, St. Louis, MO, USA) and 5 μL of propidium iodide (Sigma-Aldrich, St. Louis, MO, USA) for 15 min at room temperature in the dark. Finally, the cells were analyzed using flow cytometry (Guava easy Cyte, MERCK, Darmstadt, Germany) within 1 h. The experiments were repeated three times.

Mitochondrial Membrane Potential. HuTu 80 cells were incubated with compound 7r at concentrations IC_50_/2 (2.8 µM), and IC_50_ (5.5 µM) for 24 h Cells were harvested at 2000 rpm for 5 min and then washed twice with ice-cold PBS, followed by resuspension in JC-10 (10 µg/mL) and incubation at 37 °C for 10 min. After the cells were rinsed three times and suspended in PBS, the JC-10 (Sigma-Aldrich, St. Louis, MO, USA) fluorescence was observed using flow cytometry (Guava easy Cyte, MERCK).

Detection of Intracellular ROS

HuTu 80 cells were incubated with compound 7r at concentrations IC_50_/2 (2.8 µM) and IC_50_ (5.5 µM) for 24 h. ROS generation was investigated using a flow cytometry assay and CellROX^®^ Deep Red flow cytometry kit. For this, M-HeLa cells were harvested at 2000 rpm for 5 min and then washed twice with ice-cold PBS, followed by resuspension in 0.1 mL of medium without FBS, to which we added 0.2 μL of CellROX^®^ Deep Red (Thermo Fisher Scientific, Waltham, MA, USA) and incubated at 37 °C for 30 min After three times washing the cells and suspending them in PBS, the production of ROS in the cells was immediately monitored using flow cytometer Guava easy Cyte, MERCK).

Statistical Analysis

The IC_50_ values were calculated using the online calculator MLA—Quest Graph™ IC50 Calculator (AAT Bioquest, Inc., Pleasanton, CA, USA). Statistical analysis was performed using the Mann–Whitney test (*p* < 0.05). Tabular and graphical data are presented as mean ± SD of three independent experiments.

## 4. Conclusions

In conclusion, a series of novel, hitherto unknown 7-azacoumarin-3-carboxamides were obtained via a two-stage, two-step procedure. Additionally, the improved synthesis of 7-azacoumarin-3-carboxylic acid has been developed based on the reaction of pyridoxal with Meldrum’s acid. The proposed method does not require any catalysts, proceeds in a water medium at room temperature, and provides a target compound in high yield. The applicability of this method to the synthesis of other 7-azacoumarin derivatives has also been demonstrated. The cytotoxicity of 7-azacoumarin-3-carboxamides toward cancer and normal cell lines has been evaluated. The most potent compounds exhibited activity towards the HuTu 80 cell line equal to that of Doxorubicin. In contrast to Doxorubicin, they are much more selective and less toxic to normal cell lines (selectivity index, SI = 9–14), thus representing a novel promising scaffold for the design of the anticancer drug. The cytotoxic effect of the **7r** leader compound may be due to the induction of apoptosis through an intrinsic pathway associated with impaired mitochondrial function.

## Figures and Tables

**Scheme 3:**
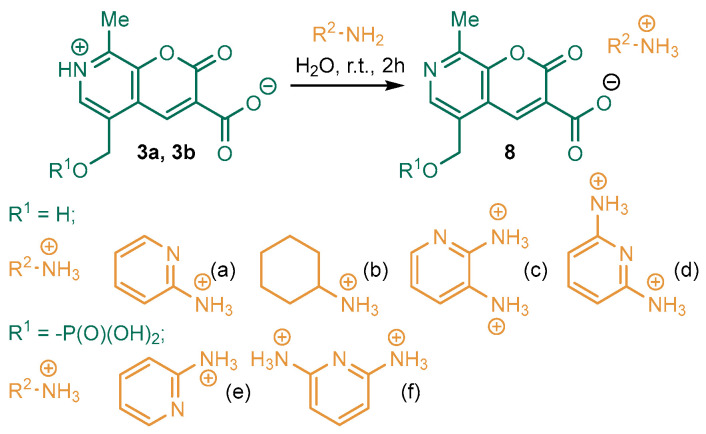
Synthesis of ammonium salts **8**.

**Table 1:** Development of the synthesis of key 7-aza-coumarin-3-carboxylic acid **3a**.

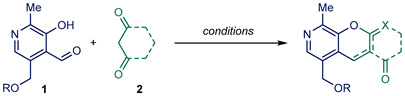				
Entry	2	Conditions	Product	Yield, % ^a^
1	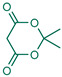	H_2_O, rt, 12 h	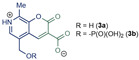	69 (**3a**)
2				86 (**3b**)
3	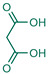	H_2_O, *catalyst*, rt, 12 h *catalyst*: citric acid, piperidine	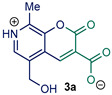	<10
4	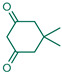	EtOH or H_2_O, 60 °C, 4 h	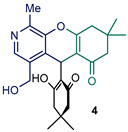	89
5	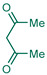	EtOH, piperidine (2% mol), AcOH (2% mol), reflux, 2 h	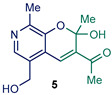	79
6		EtOH/H_2_O (3/1), KOH (40% mol), rt, 24 h	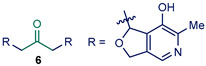	82

^a^ Isolated yield.

**Table 2:** Cytotoxic effects of 7-azacoumarins **7** on cancer and normal human cell lines ^a^.

Compounds	IC_50_ (µM)		
	Cancer Cell Lines		Normal Cell Lines
	M-HeLa	HuTu 80	Chang Liver
**3a**	75.5 ± 6.0	57.6 ± 4.6	58.0 ± 4.7
**3b**	70.6 ± 5.7	60.5 ± 5.3	70.0 ± 5.6
**7a**	>100	59.4 ± 4.8	56.6 ± 4.5
**7b**	84.4 ± 5.5	55.3 ± 3.9	46.0 ± 3.2
**7c**	67.4 ± 4.6	43.0 ± 3.5	89.4 ± 6.3
**7d**	84.0 ± 5.9	62.0 ± 4.3	88.0 ± 6.2
**7e**	54.3 ± 3.6	72.0 ± 5.2	55.3 ± 3.7
**7f**	32.0 ± 2.2	17.4 ± 1.3	48.4 ± 3.3
**7g**	14.1 ± 1.2	11.2 ± 0.9	13.9 ± 0.3
**7h**	14.8 ± 1.3	2.9 ± 0.2, SI = 10 ^b^	29.8 ± 2.0
**7i**	77.8 ± 5.4	31.0 ± 1.9	116.0 ± 8.1
**7j**	73.4 ± 5.2	32.0 ± 2.1	73.0 ± 5.5
**7k**	47.0 ± 3.1	18.1 ± 1.4	61.3 ± 4.2
**7l**	62.4 ± 4.4	52.1 ± 3.4	126.0 ± 8.8
**7m**	61.1 ± 4.2	49.7 ± 3.5	62.4 ± 4.9
**7n**	93.1 ± 6.6	61.2 ± 4.2	64.1 ± 4.8
**7o**	22.5 ± 1.8	3.1 ± 0.2, SI = 3.8 ^b^	11.8 ± 0.9
**7p**	63.0 ± 4.4	81.5 ± 5.7	37.0 ± 2.6
**7q**	84.3 ± 5.9	95.4 ± 6.5	>100
**7r**	64.5 ± 4.5	5.5 ± 0.4, SI = 14 ^b^	78.0 ± 5.9
**7s**	80.0 ± 5.7	13.8 ± 1.1, SI = 9 ^b^	125.0 ± 11
**8b**	70.4 ± 5.7	78.2 ± 6.3	81.0 ± 6.5
**8d**	>100	88.4 ± 7.0	64.1 ± 5.1
**8e**	99.5 ± 7.1	59.0 ± 4.1	78.4 ± 6.3
5-fluorouracil	62.0 ± 4.7	65.2 ± 5.4	86.3 ± 6.5
Doxorubicin	3.0 ± 0.2	3.0 ± 0.2	3.0 ± 0.1

^a^ Three independent experiments were carried out; ^b^ selectivity index, SI = IC_50_(Chang liver)/IC_50_(HuTu 80).

## Data Availability

The data presented in this study are contained within the article or in Supplementary Materials or are available upon request from the corresponding author Almir Gazizov.

## Supplementary Materials

The following supporting information can be downloaded at: https://www.mdpi.com/article/10.3390/ijms24129927/s1.
