# Supplementary material for: Synthesis and Anticancer Evaluation of Novel 7-Aza-Coumarine-3-Carboxamides
Source: Int J Mol Sci. 2023 Jun 9;24(12):9927. doi: 10.3390/ijms24129927 (PMC10298512; doi:10.3390/ijms24129927)
Supplement: Supplementary file 1 [file ijms-24-09927-s001.zip › ijms-2437746-supplementary.pdf]

Supplementary Materials

Synthesis and Anticancer Evaluation of novel 7-aza-coumarine-3-carboxamides

Alexey V. Trifonov[1], Almir S. Gazizov[1*], Anipa S. Tapalova[2], Lyudmila K. Kibardina[1], Nurbol O. Appazov[2], Alexandra D. Voloshina[1], Anastasiia S. Sapunova[1], Anna P. Luybina[1], Gulmira M. Abyzbekova[2], Alexey B. Dobrynin[1], Igor A. Litvinov[1], Akerke K. Tauekel[3], Sholpan O. Yespenbetova[2], Alexander R. Burilov[1], Michail A. Pudovik[1]

1 Arbuzov Institute of Organic and Physical Chemistry, FRC Kazan Scientific Center, Russian Academy of

Science, Arbuzova Str., 8, Kazan 420088, Russia; xahter91@gmail.com (A.V.T.); kibardina@iopc.ru (L.K.K.);

micrbi@iopc.ru (A.D.V.); luybina@iopc.ru (A.P.L.); aldo@iopc.ru (A.B.D.); litvinov@iopc.ru (I.A.L.);

burilov@iopc.ru (A.R.B.); pudovik@iopc.ru (M.A.P.)

2 Korkyt Ata Kyzylorda University, Aiteke Bi Street, 29A, Kyzylorda 120014, Kazakhstan; anipa52@mail.ru (A.S.T.);

nurasar.82@mail.ru (N.O.A.); abizgul@mail.ru (G.M.A.); sholpan-sultan@mail.ru (S.O.Y.)

3 Department of Oil, Chemistry and Nanotechnology, Kazan National Research Technological University,

Karl Marx Str., 68, Kazan 420015, Russia

* Correspondence: agazizov@iopc.ru

Table of contents

S2 §1. Synthesis of potassium 5-(hydroxymethyl)-8-methyl-2-oxo-2H-pyrano[2,3-*c*]pyridine-3-carboxylate S3–8 §2. Single crystal X-ray crystallography

S8-70 §3. ^{1}H and ^{13}C NMR spectra of the compounds

§2. Synthesis of potassium 5-(hydroxymethyl)-8-methyl-2-oxo-2H-pyrano[2,3-*c*]pyridine-3-carboxylate

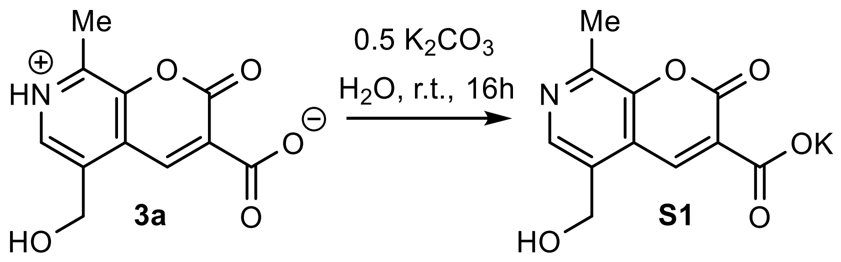

An aqueous solution of the acid **3a** (0.24 g, 1.02 mmol) and a potassium carbonate (0.07 g, 0.51 mmol) was stirred at room temperature for 16 hours. The solvent was removed from the reaction mixture to give title compound as a white solid, that did not require additional purification. Yield: 96% (0.27g); mp: > 300 ºC. IR (ν cm^{-1}): 569,597, 652, 962, 746, 772, 815, 839, 945, 980, 1012, 1072, 1098, 1139, 1207, 1268, 1286, 1331, 1352, 1415, 1608, 1627, 1662, 1756, 2863, 3252, 3362, 3535. ^{1}H NMR (DMSO-d_6) δ ppm: 2.53 (s, 3H, CH_3), 4.69 (s, 2H, $\underline{CH_2}OH$), 5.66 (br.s, 1H, $CH_2\underline{OH}$), 7.96 (s, 1H, CH_{arom}), 8.22 (s, 1H, CH). ^{13}C NMR (DMSO-d_6) δ ppm: 18.94, 58.56, 123.40, 131.28, 132.51, 136.00, 142.56, 145.86, 147.11, 158.13, 165.35. MALDI TOF - MS *m/z*: 273.9 $[M]^+$. Calculated for $C_{11}H_8KNO_5$, %: C, 48.35; H, 2.95; K, 14.31, N, 5.13. Found, %: C, 48.55; H, 3.05; K, 14.03, N, 5.21.

The structure of the salt **S1** was additionally proved by x-ray analysis.

§2. Single crystal X-ray crystallography

Table S1. Data collection and selected refinement parameters for the obtained complexes.

	**4**	**5**	**7a**
CCDC number	2266952	2266953	2266954
Chemical formula	$C_{24}H_{29}NO_5$, C_2H_6OS	$C_{13}H_{15}NO_4$	$C_{17}H_{13}ClN_2O_3$
M_r	489.61	249.26	328.74
Crystal system, space group	Triclinic, *P*-1	Orthorhombic, *P*bca	Triclinic, *P*-1
Temperature (K)	110	296	100
a, b, c (Å)	9.4356(7), 10.3116(6), 14.6168(10)	12.1992(9), 14.0296(10), 14.0897(12)	7.3406(4), 12.9361(7), 15.9503(8)
α, β, γ (°)	78.578(3), 72.115(2), 72.115(2)	90, 90, 90	91.274(2), 99.282(2), 104.960(2),
V ($Å^3$)	1246.75(15)	2411.5(3)	1440.88(13)
Z	2	8	4
ρ_{calc}(mg/mm^3)	1.304	1.373	1.515
μ (mm^{-1})	0.171	0.102	0.283
Crystal size (mm)	0.10 x 0.30 x 0.34	0.30 x 0.30 x 0.35	0.10 x 0.17 x 0.23
T_{min}, T_{max}	0.6774, 0.7461	0.6774, 0.7461	0.5232, 0.7457
No. of measured, independent and observed [$I > 2\sigma(I)$] reflections	66018, 7268, 6113	61026, 3681, 2920	71292, 7125, 5321
R_{int}	0.054	0.057	0.128
θ range for data collection (°)	2.1 < θ < 30.0	2.6 < θ < 30.6	2.0 < θ < 28.3
$R[F^2>2\sigma(F^2)]$, $wR(F^2)$, S	0.0359, 0.1015, 1.04	0.0510, 0.1585, 1.11	0.0624, 0.1633, 1.04
No. of reflections	7268	3681	7125
No. of parameters	322	168	417
Largest diff. peak and hole (e $Å^{-3}$)	0.48, -0.27	0.51, -0.25	0.72, -0.44

	7f	8b	S1
CCDCnumber	2266955	2266956	2266957
Chemical formula	$C_{23}H_{18}ClN_2O_3$, Cl	$C_{11}H_8NO_5$, $C_6H_{14}N$	$C_{11}H_{10}KNO_6$
M_r	441.29	334.37	291.30
Crystal system, space group	Monoclinic, $P2_1/c$	Monoclinic, $P2_1/c$	Triclinic, *P*-1
Temperature (K)	110	296	300
a, b, c (Å)	14.0682(11),<br>8.0618(6),<br>18.6999(15)	13.7630(9),<br>6.7600(4),<br>17.1279(11)	6.5416(4),<br>8.6276(5),<br>10.6404(6)
α, β, γ (°)	90,<br>107.846(3),<br>90	90,<br>96.677(2),<br>90	87.983(2),<br>89.806(2),<br>69.503(2),
V (Å^3)	2018.8(3)	1582.74(17)	562.13(6)
Z	4	4	2
ρ_{calc}(mg/mm^3)	1.452	1.403	1.721
μ (mm^{-1})	0.350	0.104	0.497
Crystal size (mm)	0.03 x 0.11 x 0.13	0.16 x 0.22 x 0.23	0.12 x 0.17 x 0.39
T_{min}, T_{max}	0.6855, 0.7128	0.3157, 0.7474	0.6831, 0.7417
No. of measured, independent and observed [$I > 2\sigma(I)$] reflections	88540, 5013, 3662	21831, 3358, 3008	24728, 2174, 2099
R_{int}	0.128	0.064	0.042
θ range for data collection (°)	2.3 < θ < 28.3	2.7 < θ < 27.0	1.9 < θ < 26.0
$R[F^2>2\sigma(F^2)]$, $wR(F^2)$, S	0.0358, 0.1041, 0.93	0.0439, 0.1247, 1.09	0.0249, 0.0671, 1.08
No. of reflections	5013	3358	2174
No. of parameters	276	220	185
Largest diff. peak and hole (e Å^{-3})	0.33, -0.39	0.30, -0.26	0.42, -0.44

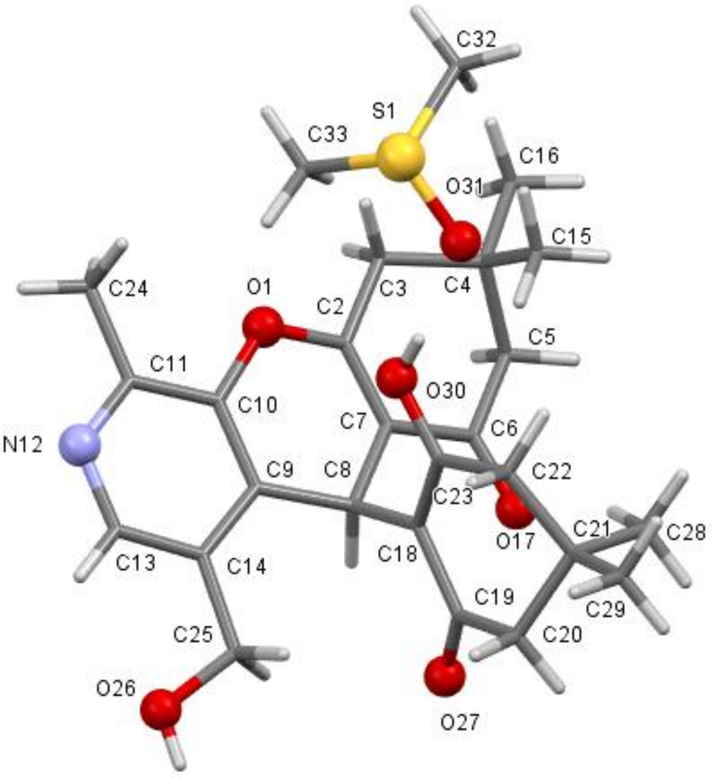

Figure S1. Structure of **4**·according to single crystal XRD analysis.

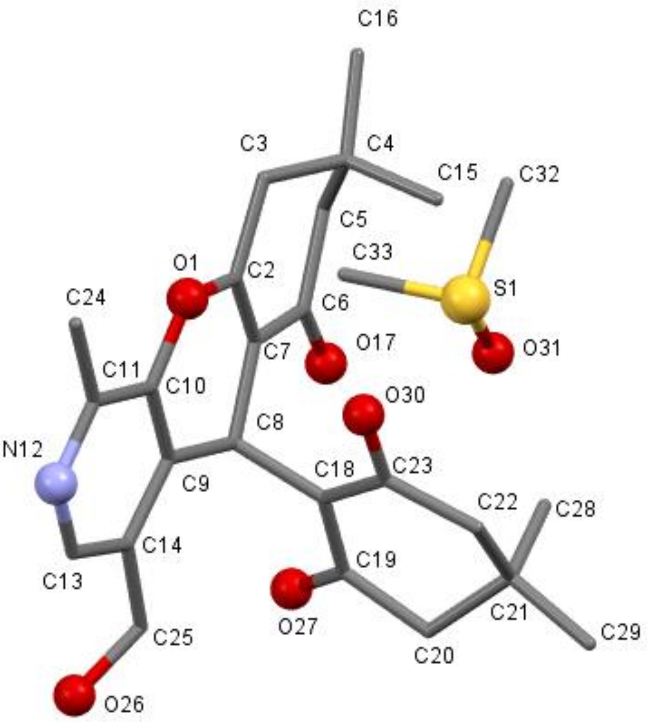

Figure S2. Structure of **4**·according to single crystal XRD analysis (hydrogen atoms are omitted for clarity).

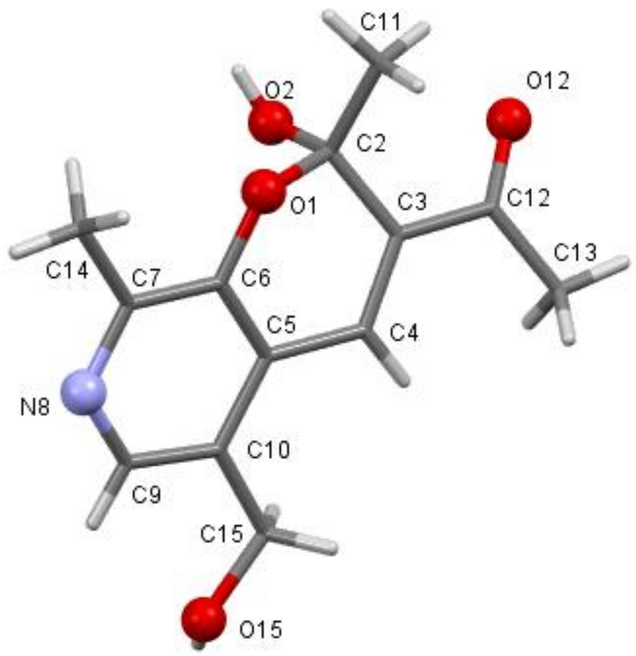

Figure S3. Structure of **5**·according to single crystal XRD analysis.

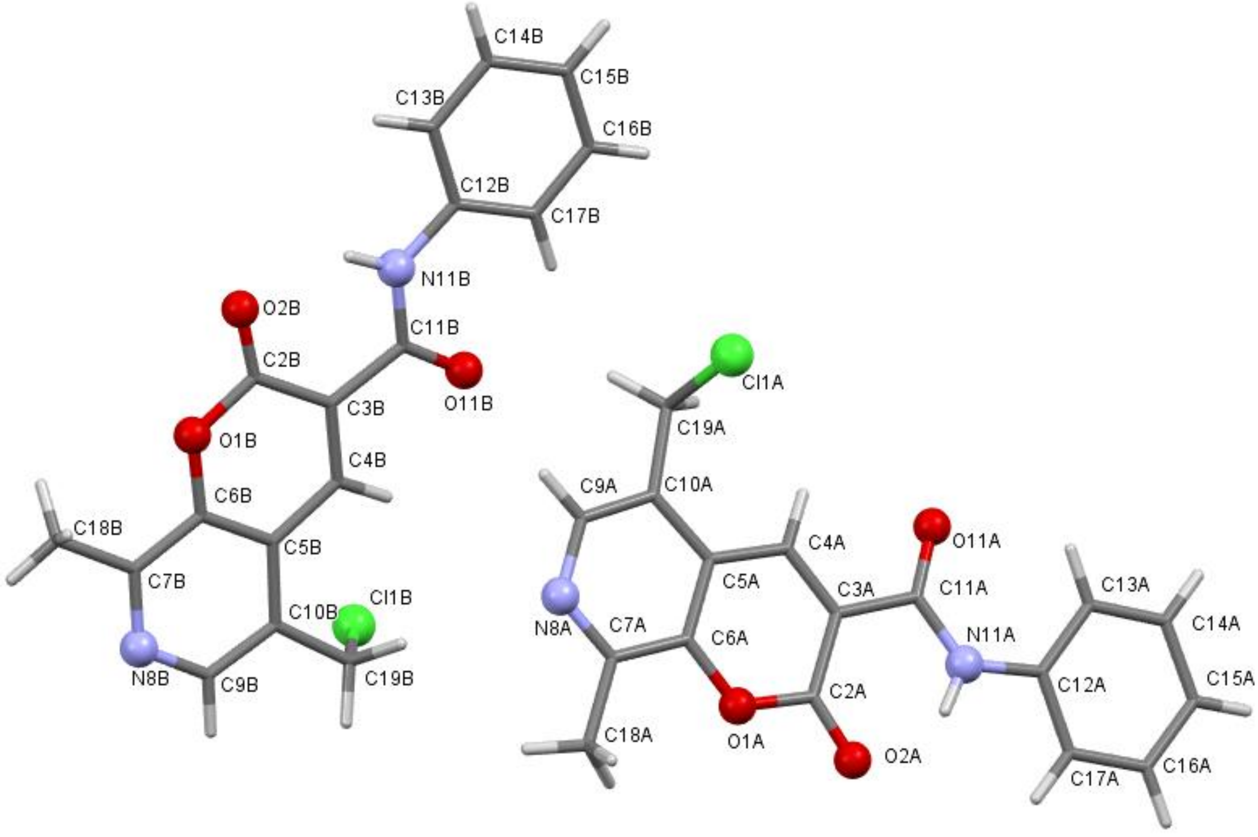

Figure S4. Structure of **7a**·according to single crystal XRD analysis.

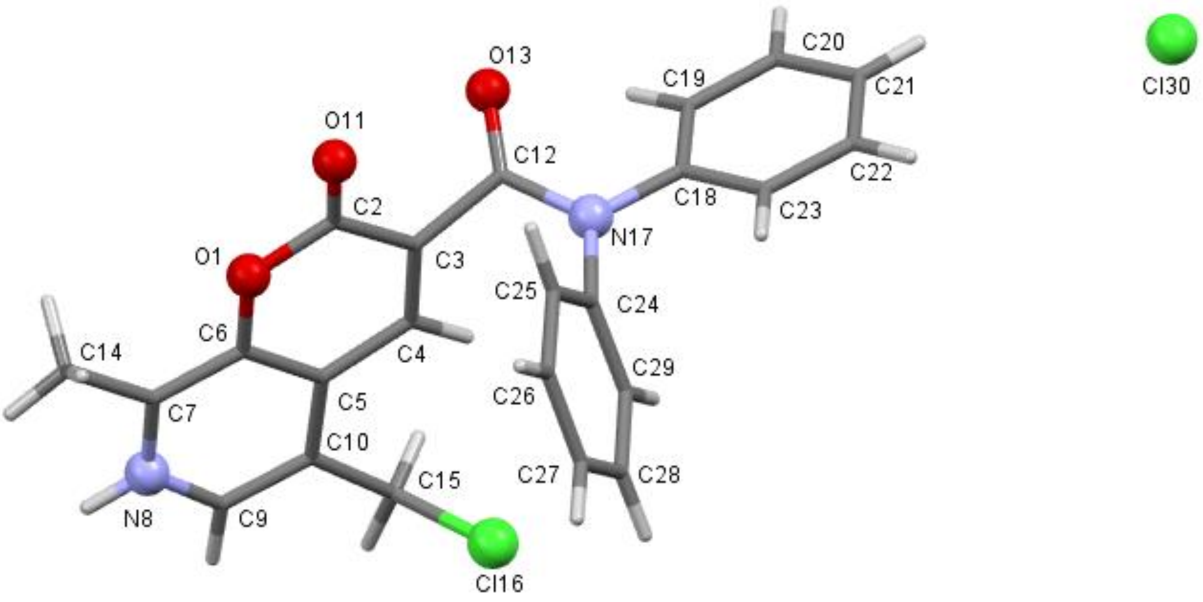

Figure S5. Structure of **7f**·according to single crystal XRD analysis.

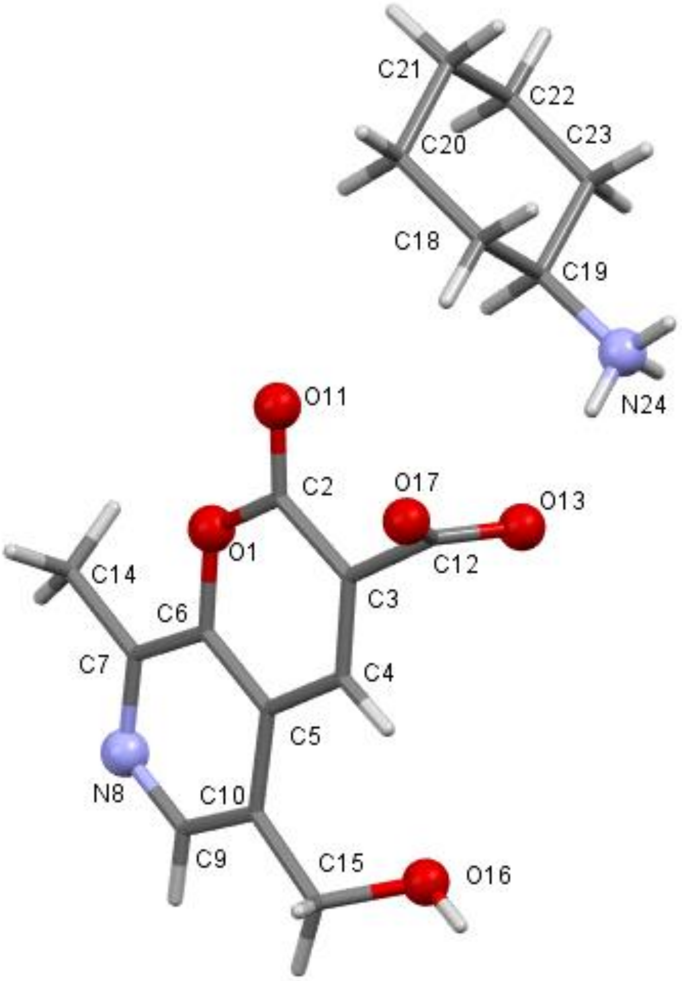

Figure S6. Structure of **8b**·according to single crystal XRD analysis.

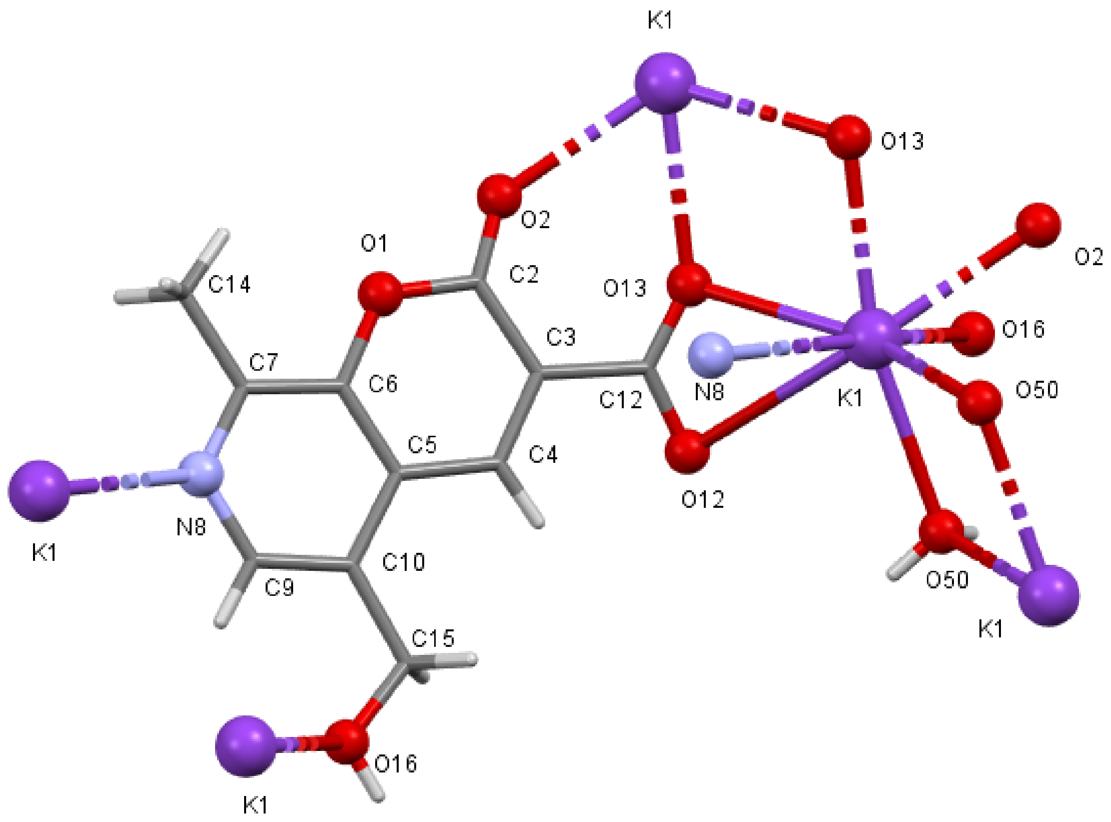

Figure S7. Structure of **A**·according to single crystal XRD analysis.

§3. ^{1}H and ^{13}C NMR spectra of the compounds

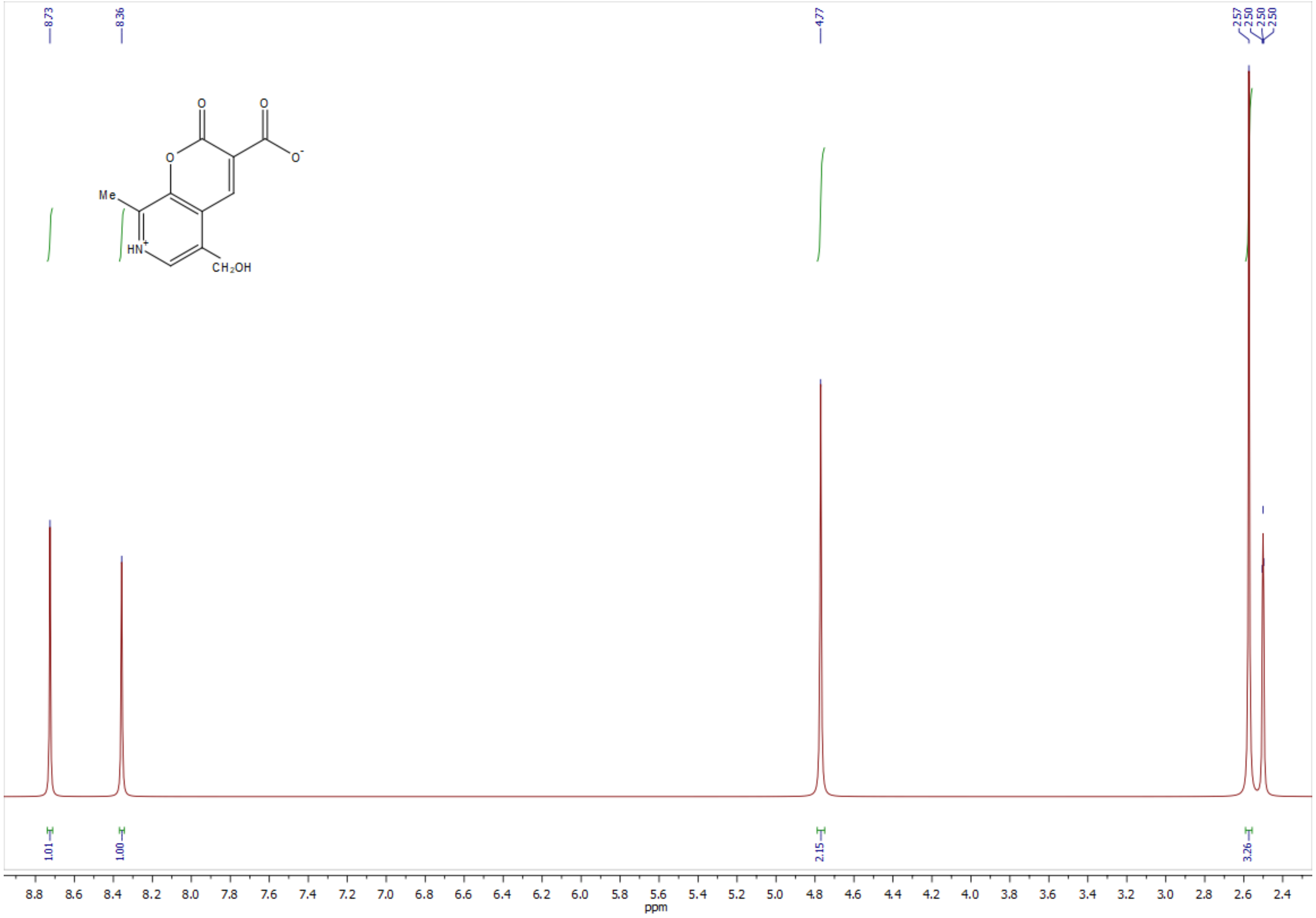

Figure S8. ^{1}H NMR spectra of the compound **3a** (600 MHz, DMSO-d_6)

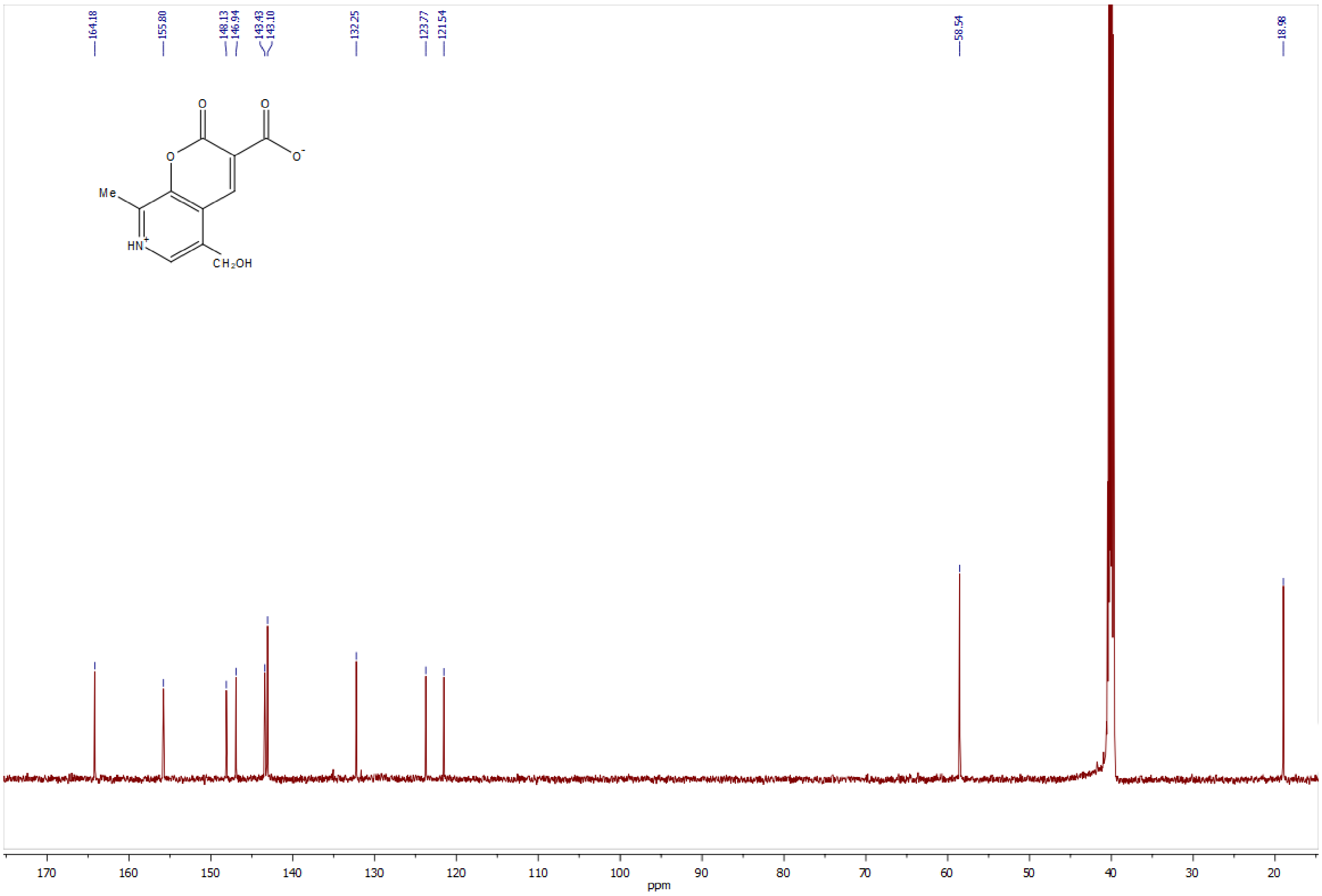

Figure S9. ^{13}C NMR spectra of the compound **3a** (600 MHz, DMSO-d_6)

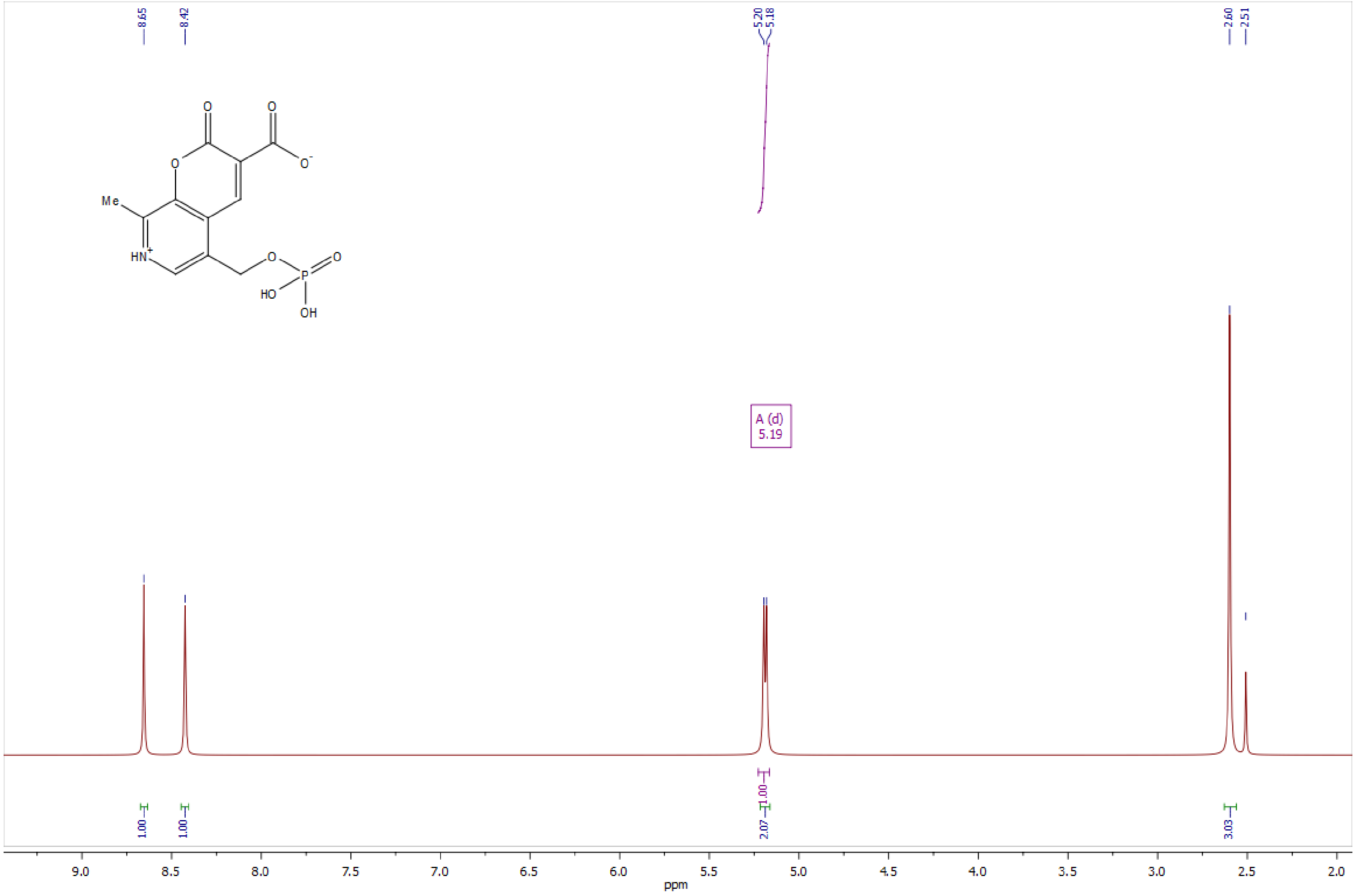

^{1}H NMR (400 MHz, DMSO-d_6) δ 5.19 (d, J = 7.7 Hz, 1H).

Figure S10. ^{1}H NMR spectra of the compound **3b** (400 MHz, DMSO-d_6)

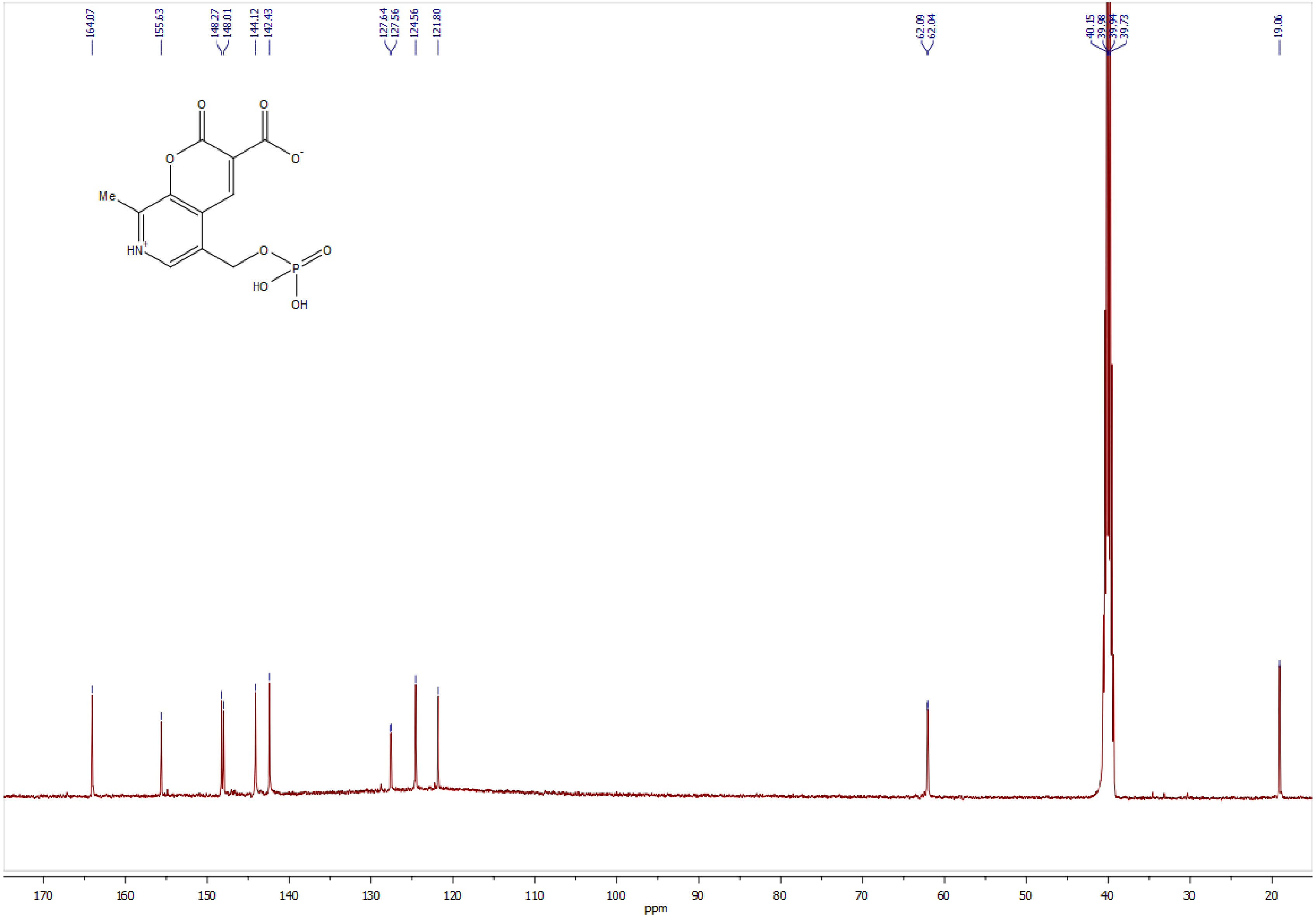

Figure S11. ^{13}C NMR spectra of the compound **3b** (400 MHz, DMSO-d_6)

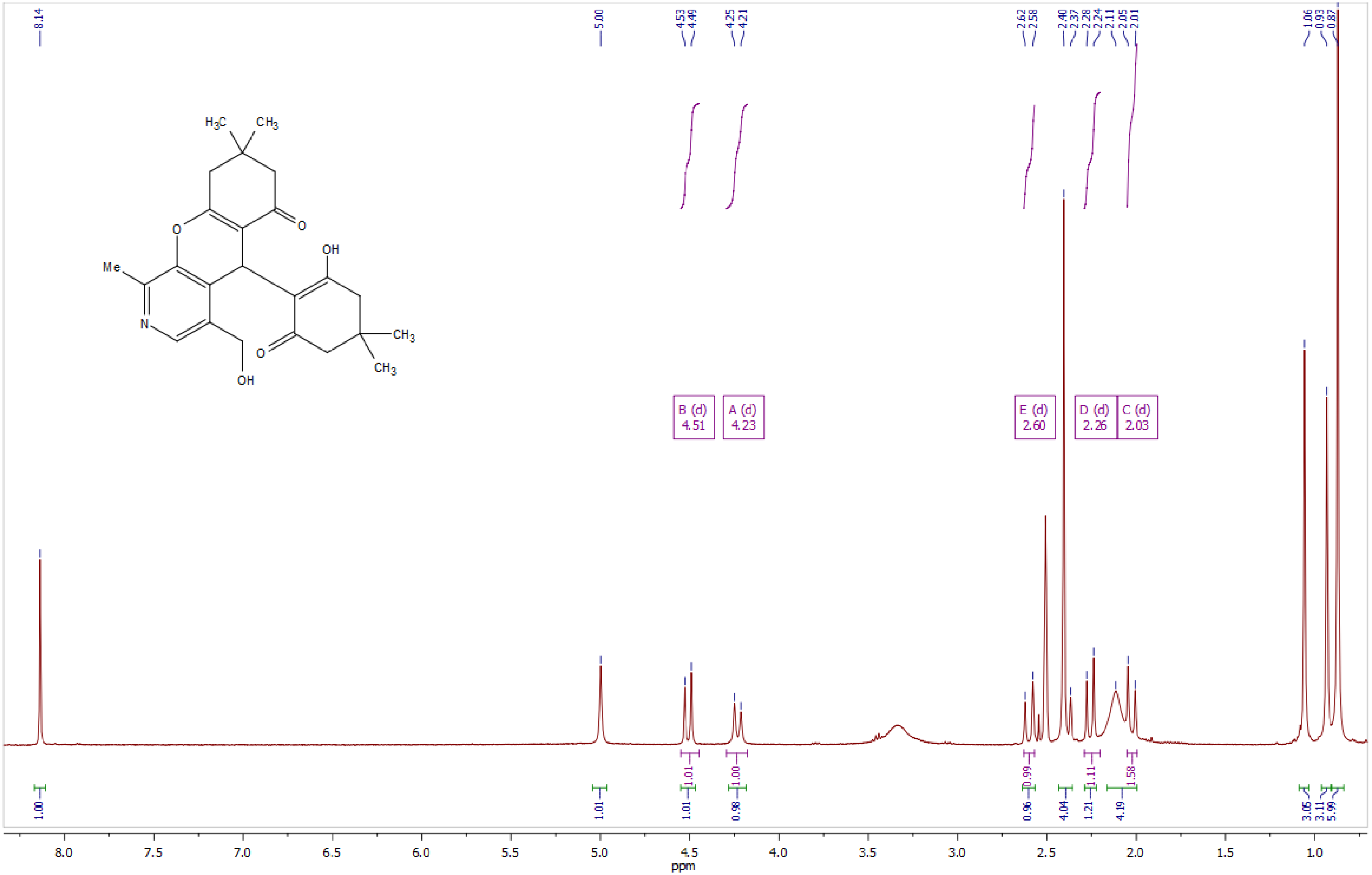

^{1}H NMR (400 MHz, DMSO-d_6) δ 4.51 (d, J = 14.5 Hz, 1H), 4.23 (d, J = 14.5 Hz, 1H), 2.60 (d, J = 17.3 Hz, 1H), 2.26 (d, J = 15.9 Hz, 1H), 2.03 (d, J = 16.0 Hz, 2H).

Figure S12. ^{1}H NMR spectra of the compound **4** (400 MHz, DMSO-d_6)

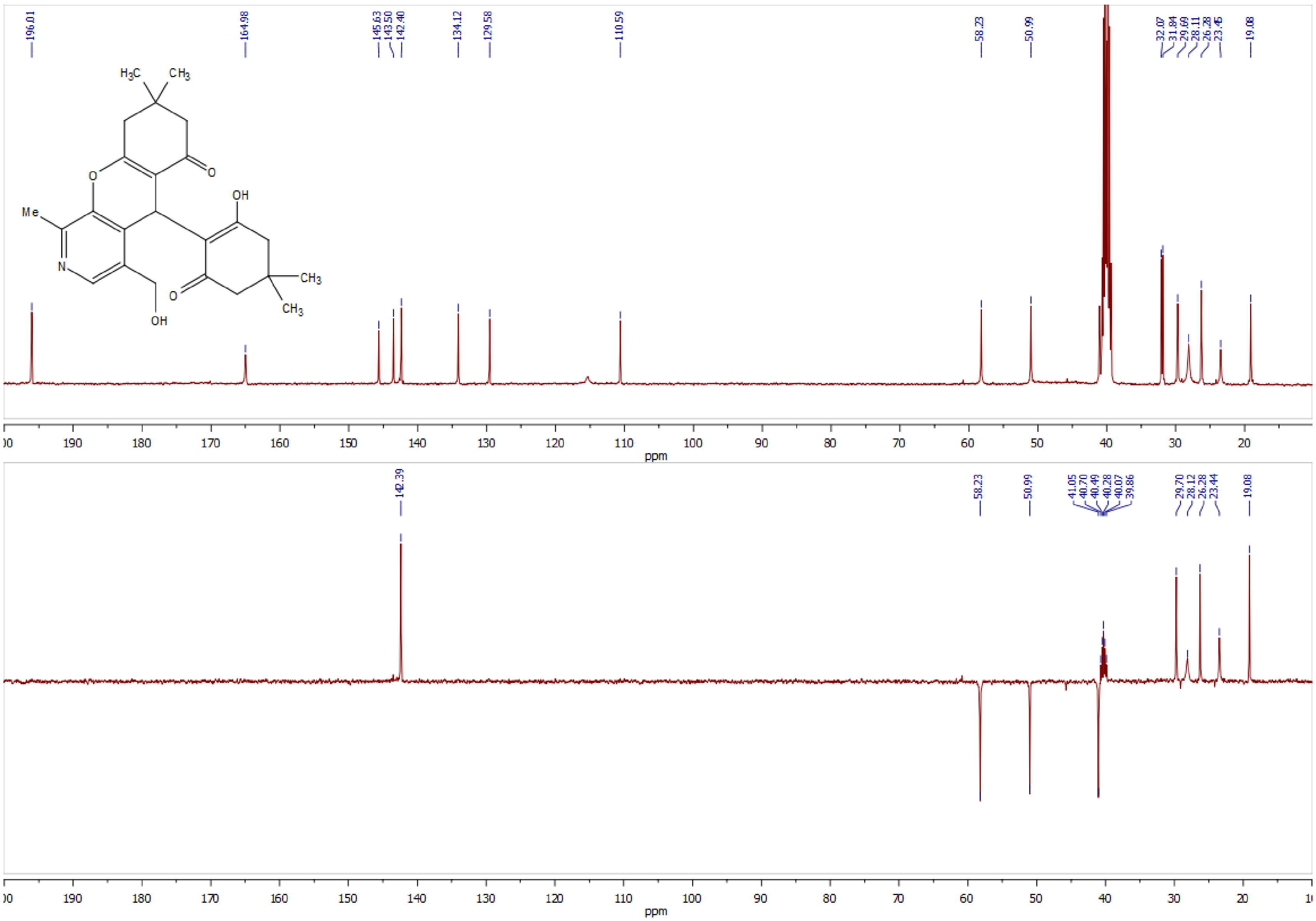

Figure S13. ^{13}C NMR spectra of the compound **4** (400 MHz, DMSO-d_6)

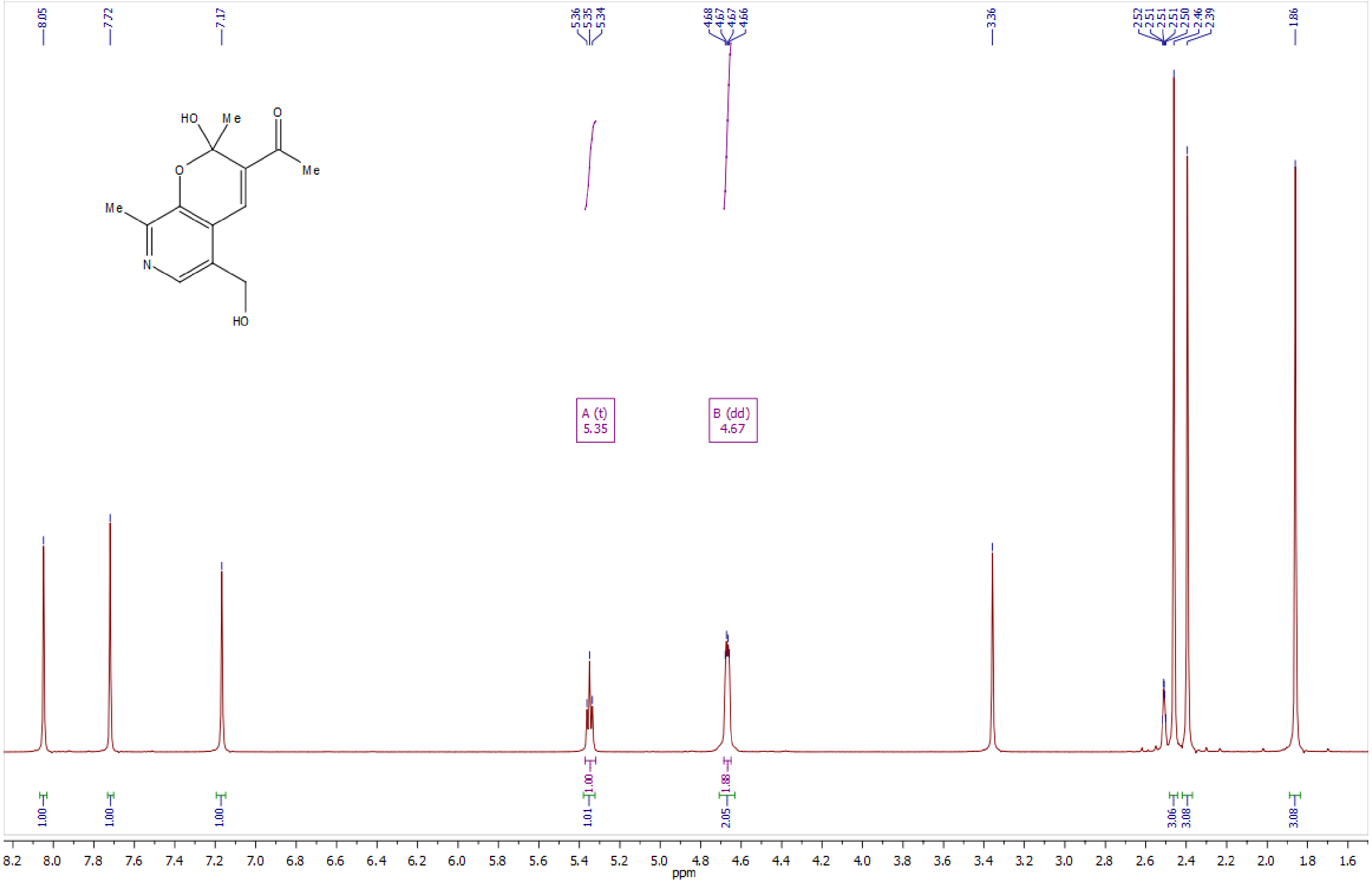

^{1}H NMR (400 MHz, DMSO-d_6) δ 5.35 (t, J = 5.5 Hz, 1H), 4.67 (dd, J = 5.6, 2.3 Hz, 2H).

Figure S14. ^{1}H NMR spectra of the compound **5** (400 MHz, DMSO-d_6)

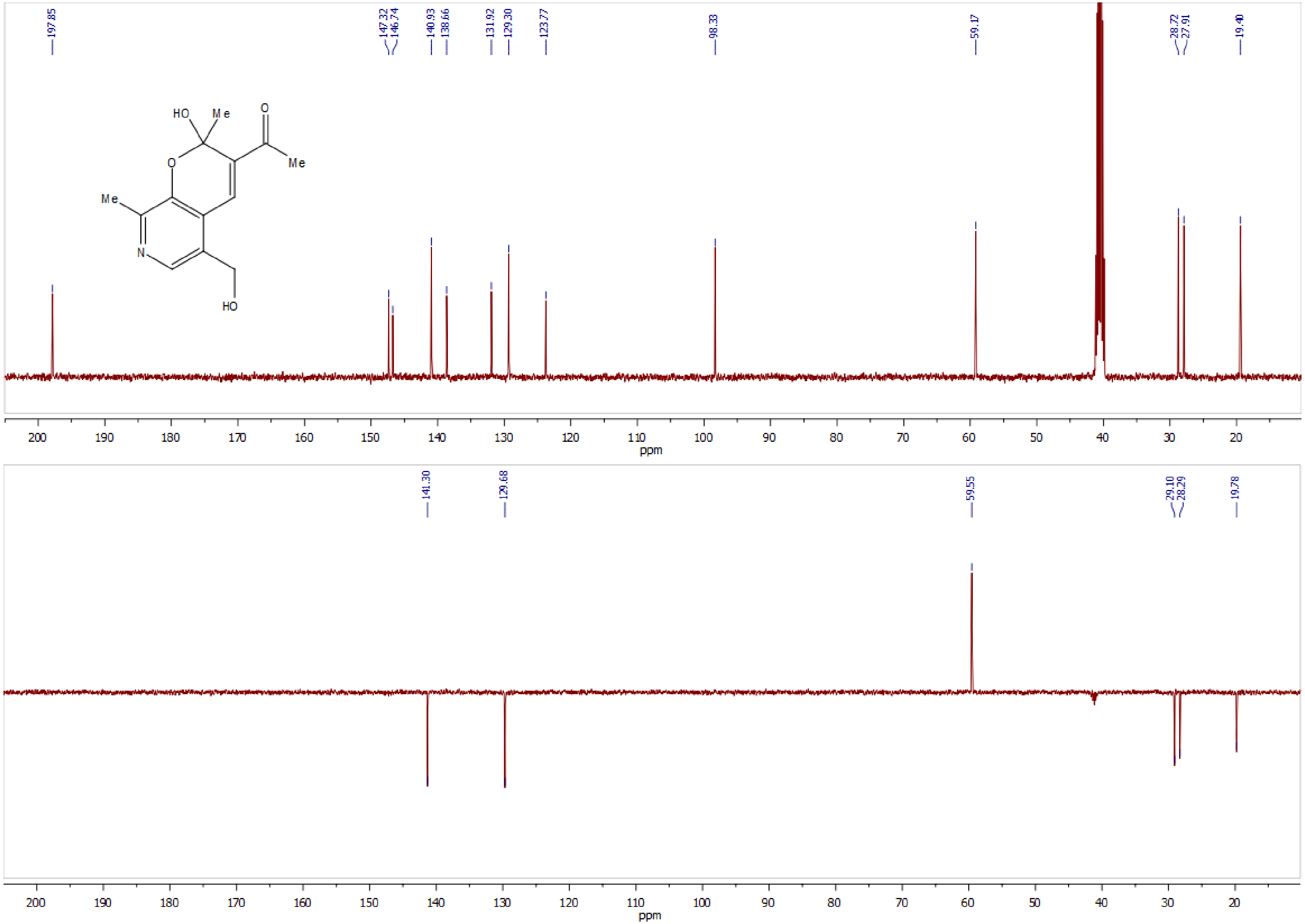

Figure S15. ^{13}C NMR spectra of the compound **5** (400 MHz, DMSO-d_6)

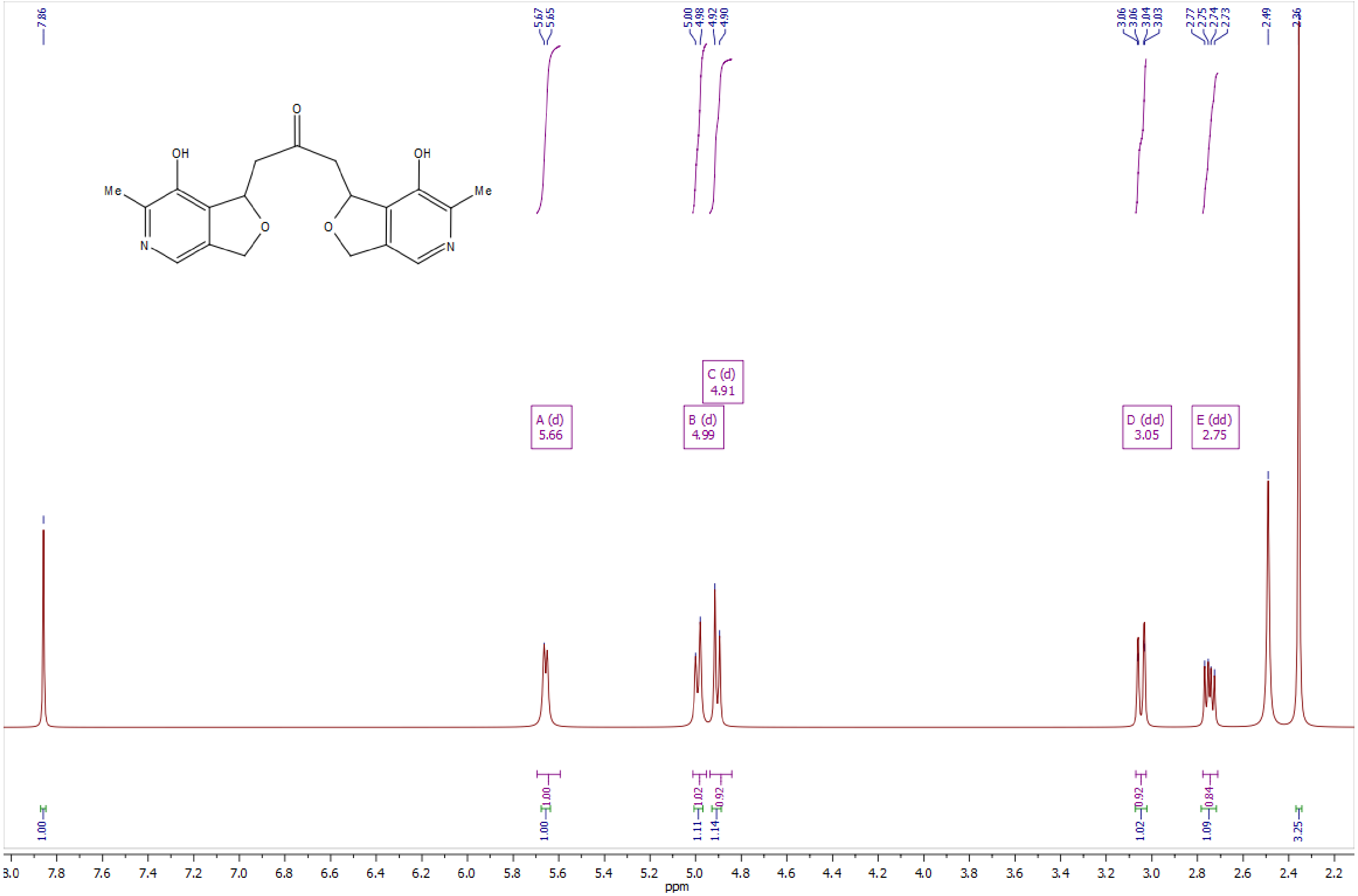

^{1}H NMR (600 MHz, DMSO-d_6) δ 5.66 (d, J = 9.1 Hz, 1H), 4.99 (d, J = 12.2 Hz, 1H), 4.91 (d, J = 12.3 Hz, 1H), 3.05 (dd, J = 16.3, 2.4 Hz, 1H), 2.75 (dd, J = 16.3, 9.2 Hz, 1H).

Figure S16. ^{1}H NMR spectra of the compound **6** (600 MHz, DMSO-d_6)

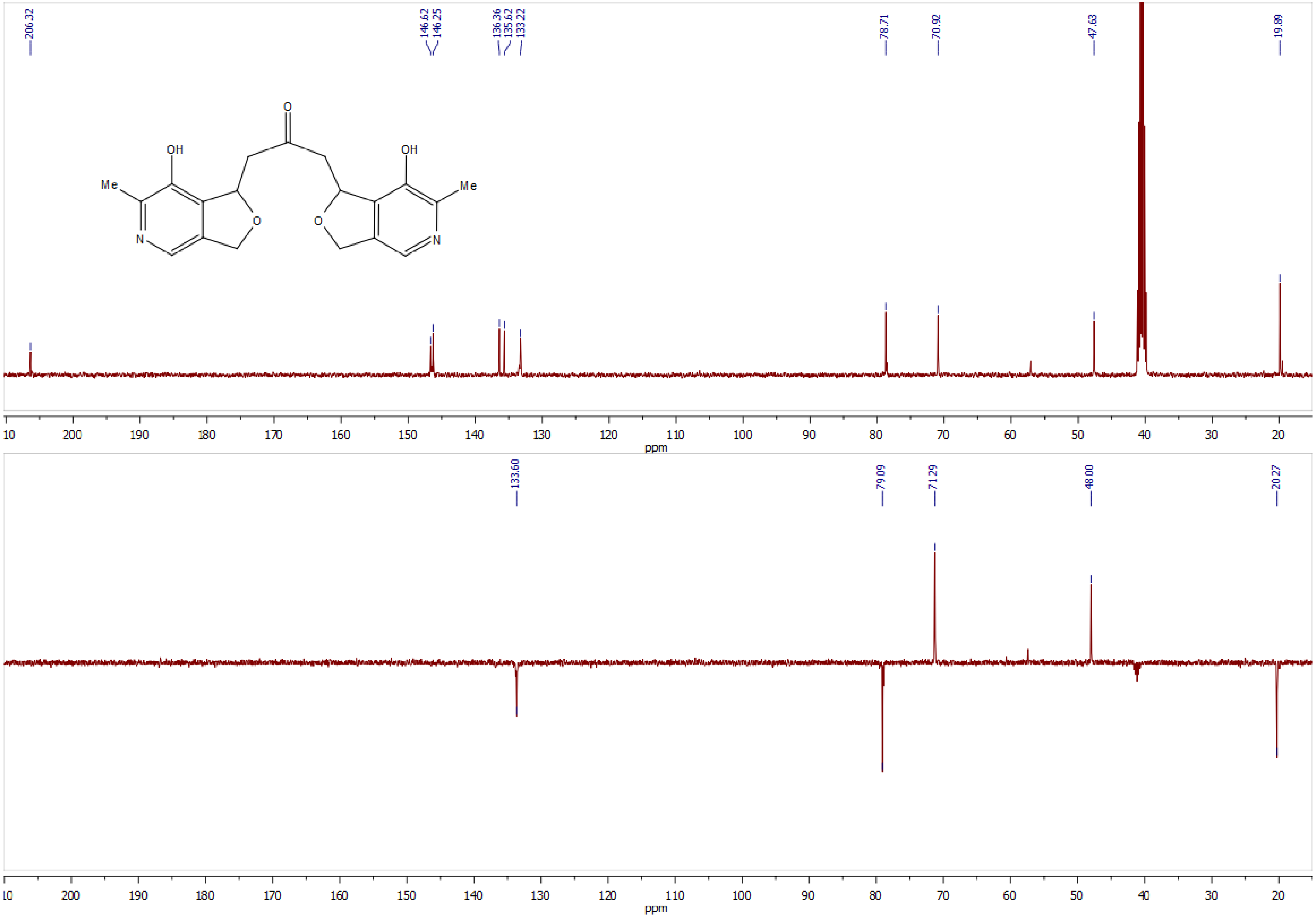

Figure S17. ^{13}C NMR spectra of the compound **6** (600 MHz, DMSO-d_6)

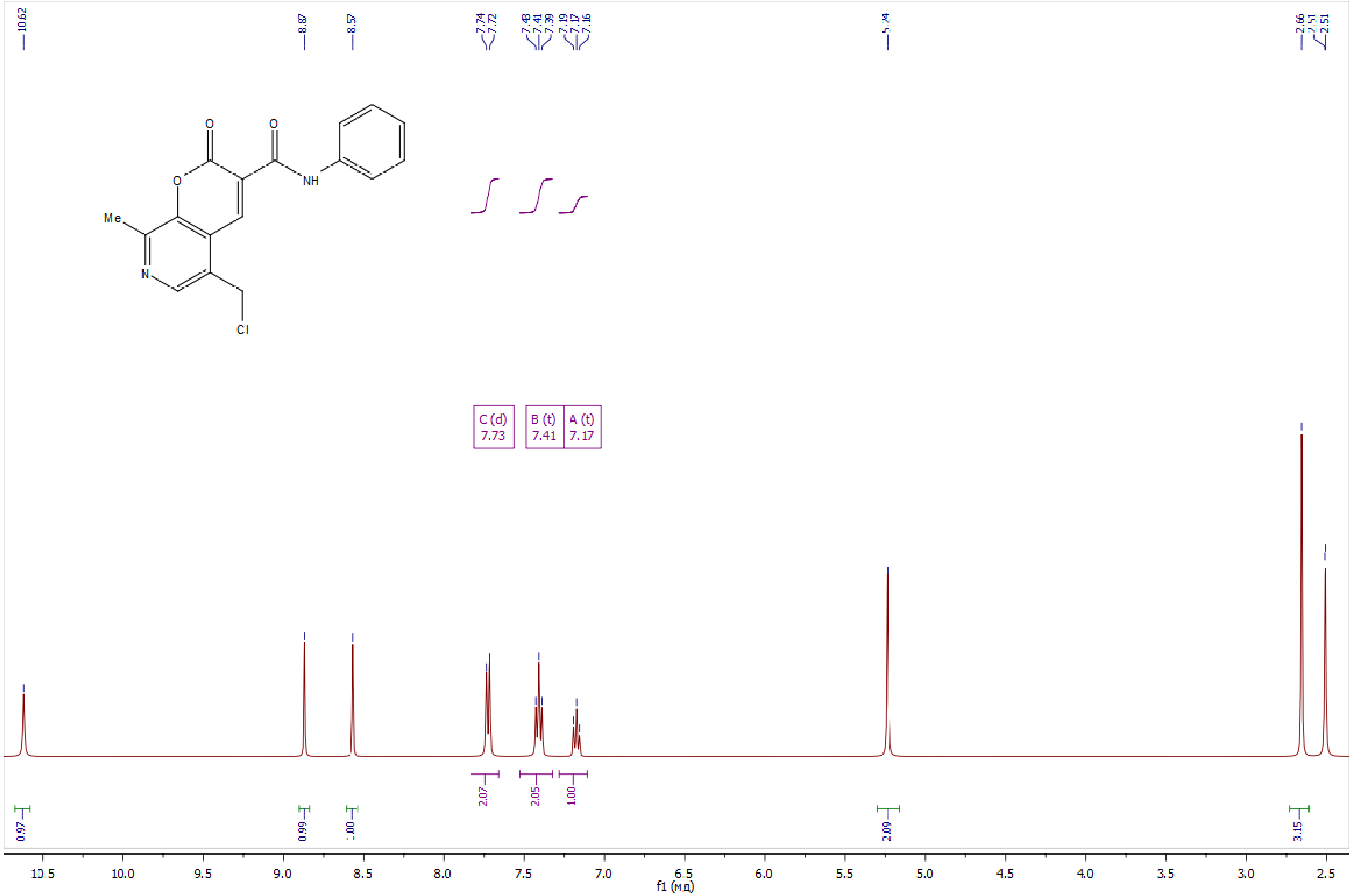

^{1}H NMR (400 MHz, DMSO-d_6) δ 7.73 (d, J = 8.0 Hz, 2H), 7.41 (t, J = 7.7 Hz, 2H), 7.17 (t, J = 7.4 Hz, 1H).

Figure S18. ^{1}H NMR spectra of the compound **7a** (400 MHz, DMSO-d_6)

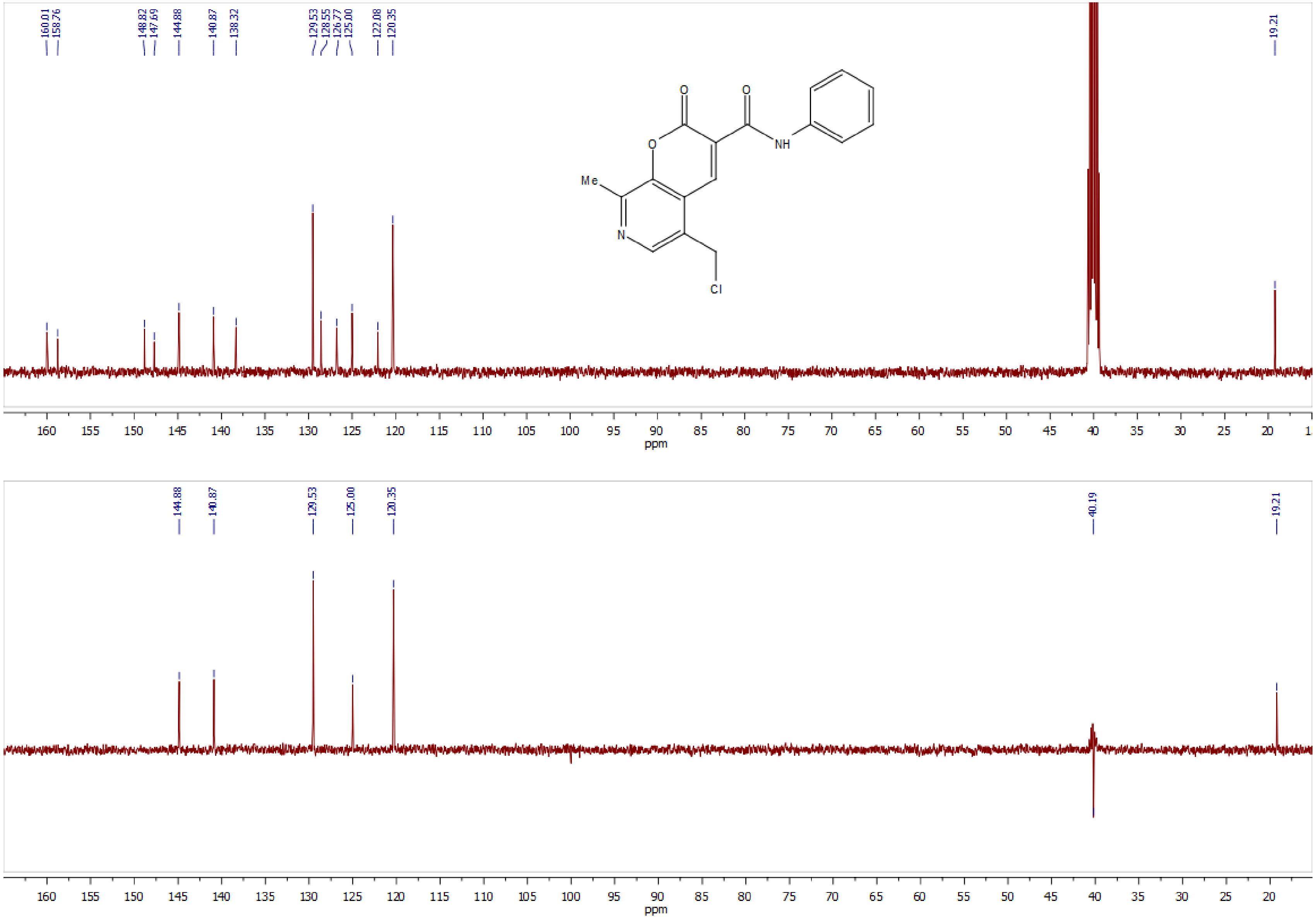

Figure S19. ^{13}C NMR spectra of the compound **7a** (400 MHz, DMSO-d_6)

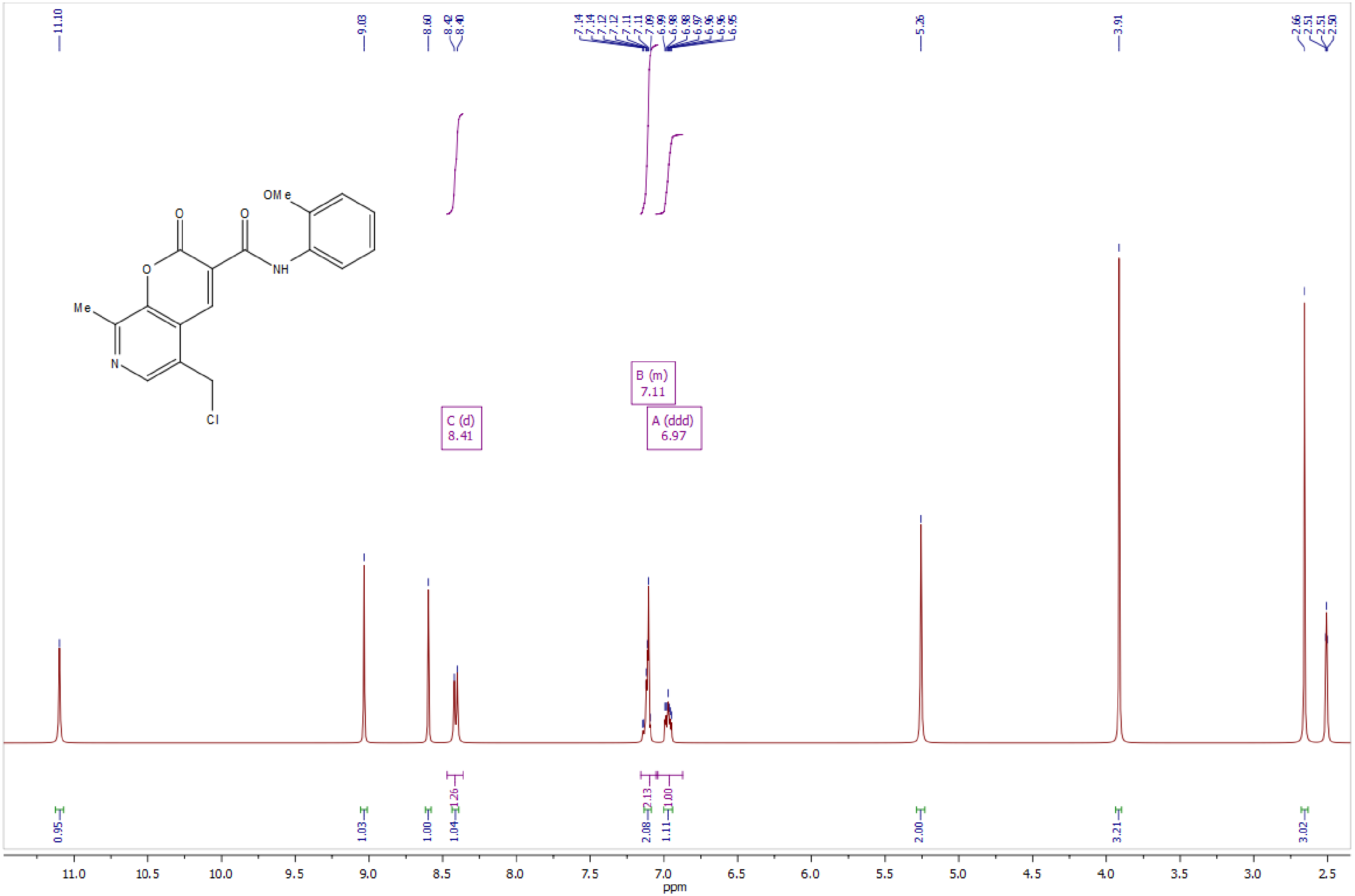

^{1}H NMR (400 MHz, DMSO-d_6) δ 8.41 (d, J = 7.8 Hz, 1H), 7.16 – 7.04 (m, 2H), 6.97 (ddd, J = 8.5, 5.9, 2.9 Hz, 1H).

Figure S20. ^{1}H NMR spectra of the compound **7b** (400 MHz, DMSO-d_6)

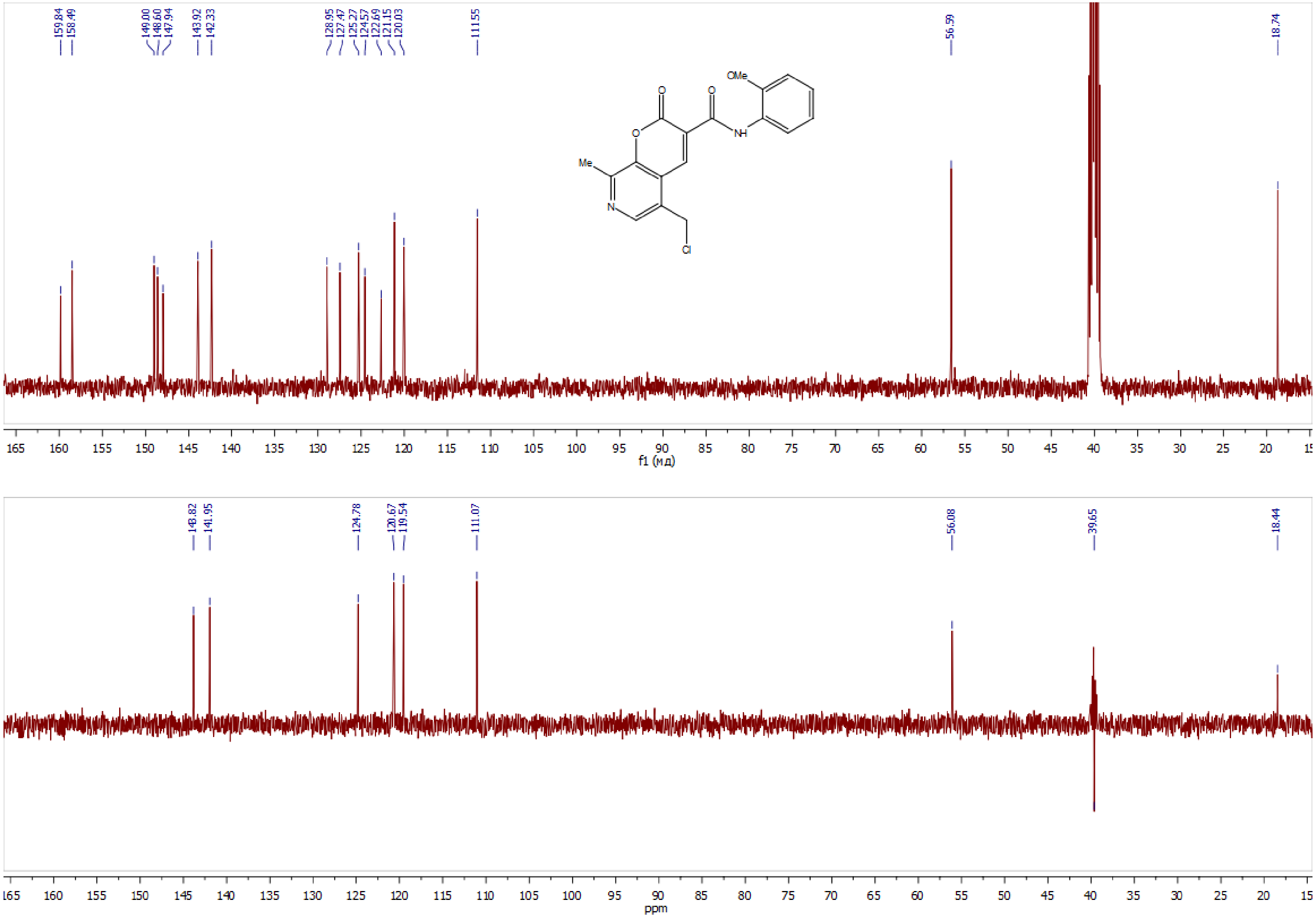

Figure S21. ^{13}C NMR spectra of the compound **7b** (400 MHz, DMSO-d_6)

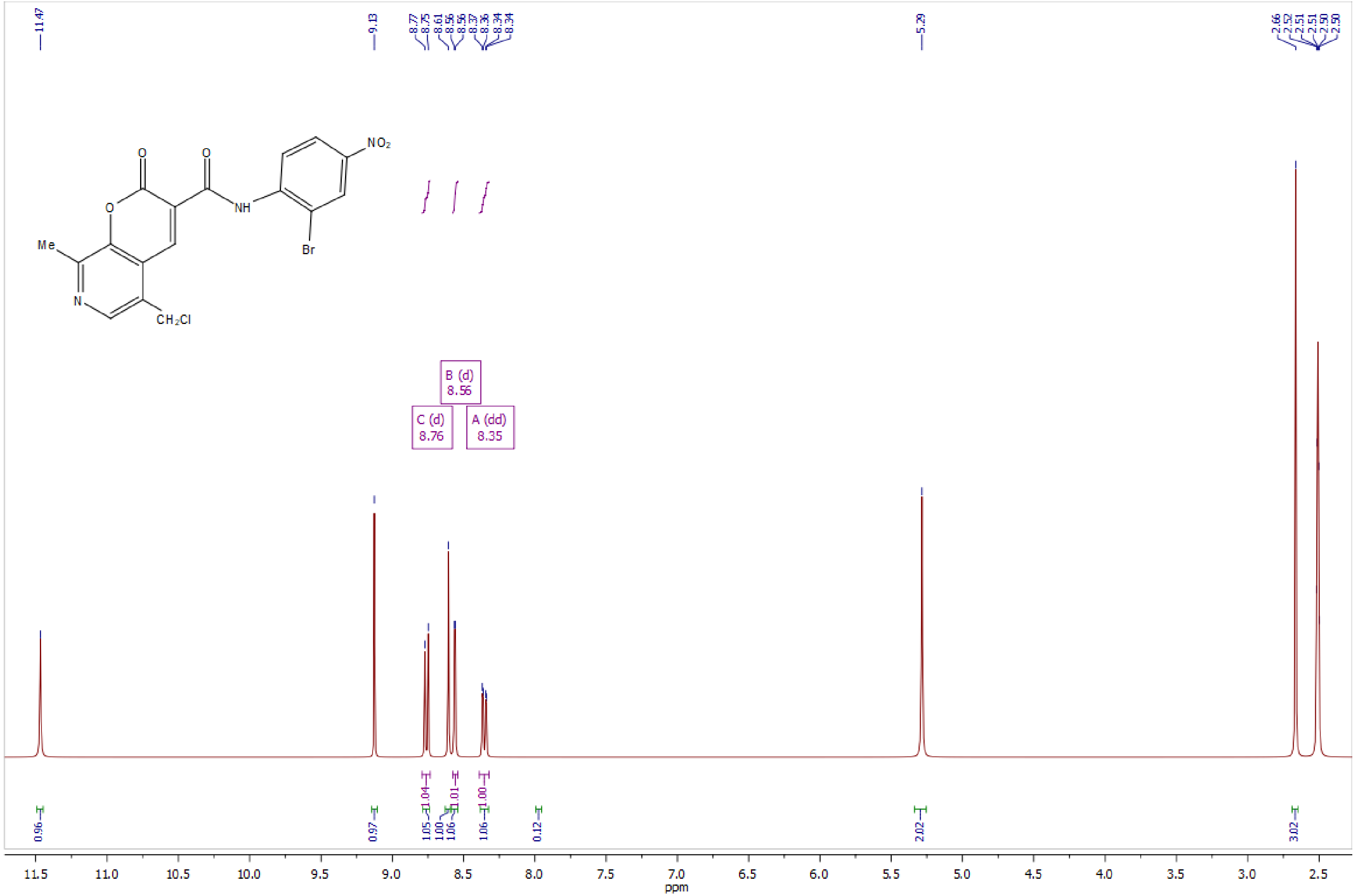

^{1}H NMR (400 MHz, DMSO-d_6) δ 8.76 (d, J = 9.2 Hz, 1H), 8.56 (d, J = 2.6 Hz, 1H), 8.35 (dd, J = 9.2, 2.7 Hz, 1H).

Figure S22. ^{1}H NMR spectra of the compound **7c** (400 MHz, DMSO-d_6)

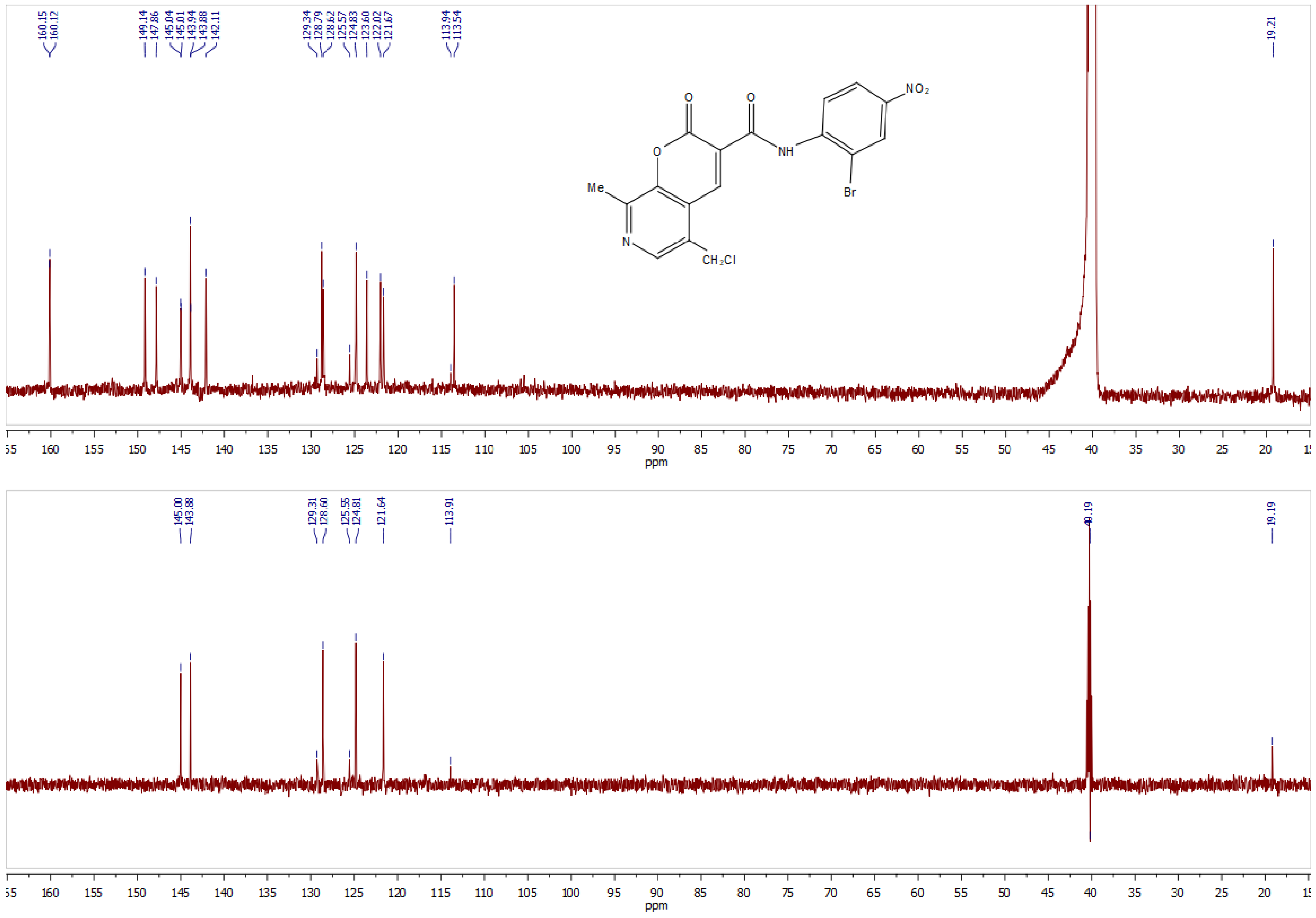

Figure S23. ^{13}C NMR spectra of the compound **7c** (400 MHz, DMSO-d_6)

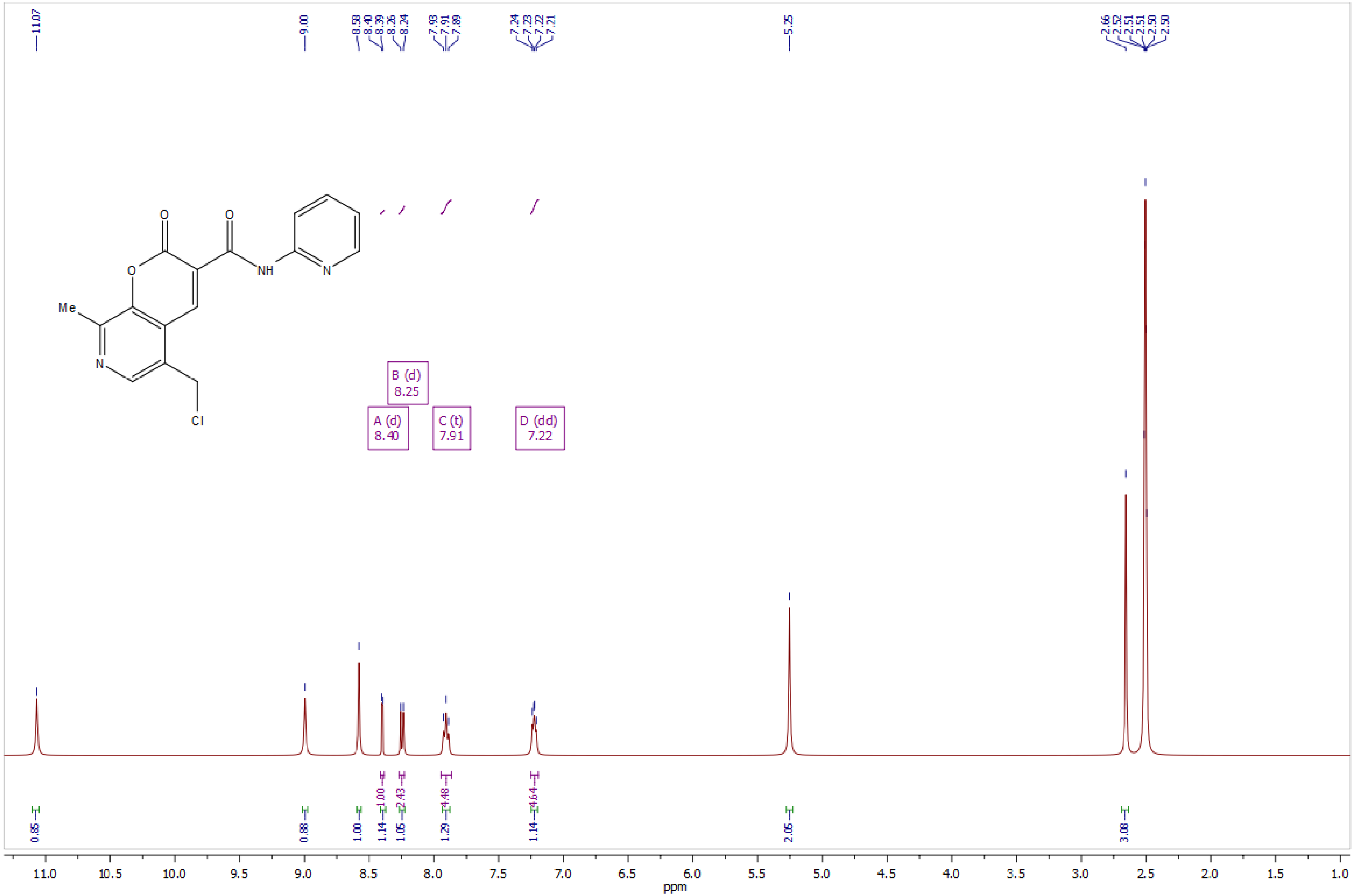

^{1}H NMR (400 MHz, DMSO-d_6) δ 8.40 (d, J = 4.0 Hz, 1H), 8.25 (d, J = 8.1 Hz, 2H), 7.91 (t, J = 7.9 Hz, 4H), 7.22 (dd, J = 7.2, 5.1 Hz, 5H).

Figure S24. ^{1}H NMR spectra of the compound **7d** (400 MHz, DMSO-d_6)

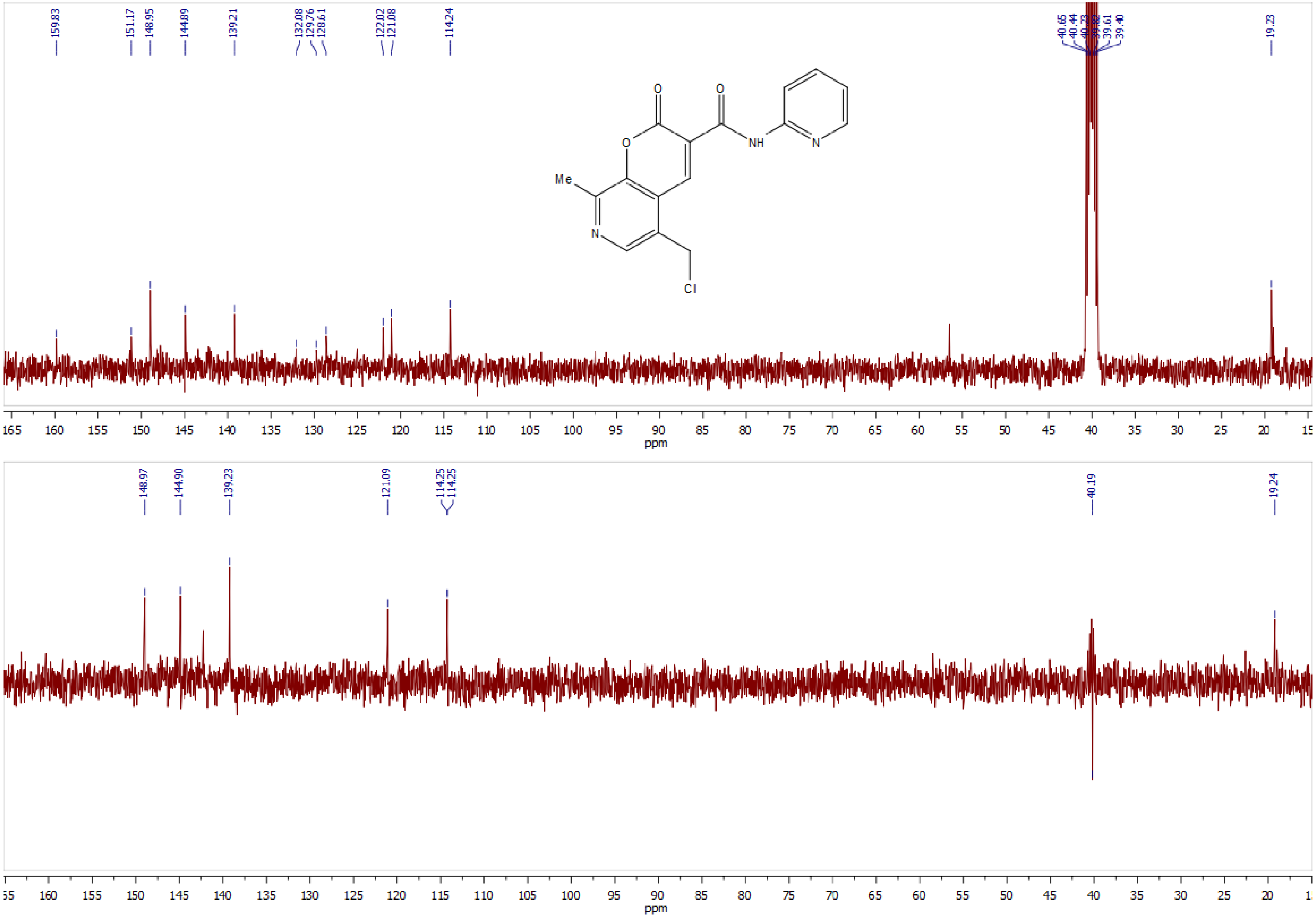

Figure S25. ^{13}C NMR spectra of the compound **7d** (400 MHz, DMSO-d_6)

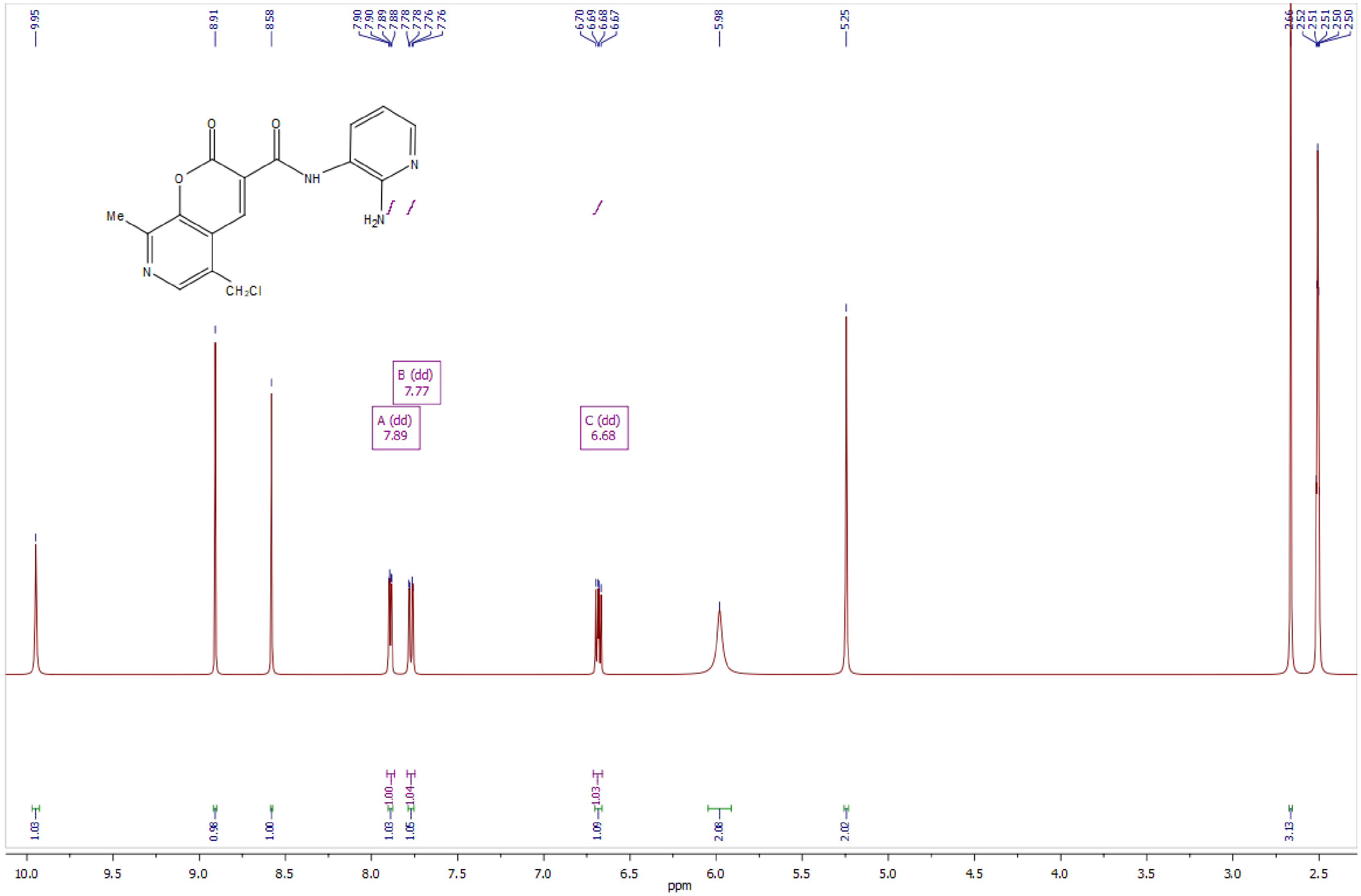

^{1}H NMR (400 MHz, DMSO-d_6) δ 7.89 (dd, J = 5.0, 1.7 Hz, 1H), 7.77 (dd, J = 7.6, 1.7 Hz, 1H), 6.68 (dd, J = 7.7, 5.0 Hz, 1H).

Figure S26. ^{1}H NMR spectra of the compound **7e** (400 MHz, DMSO-d_6)

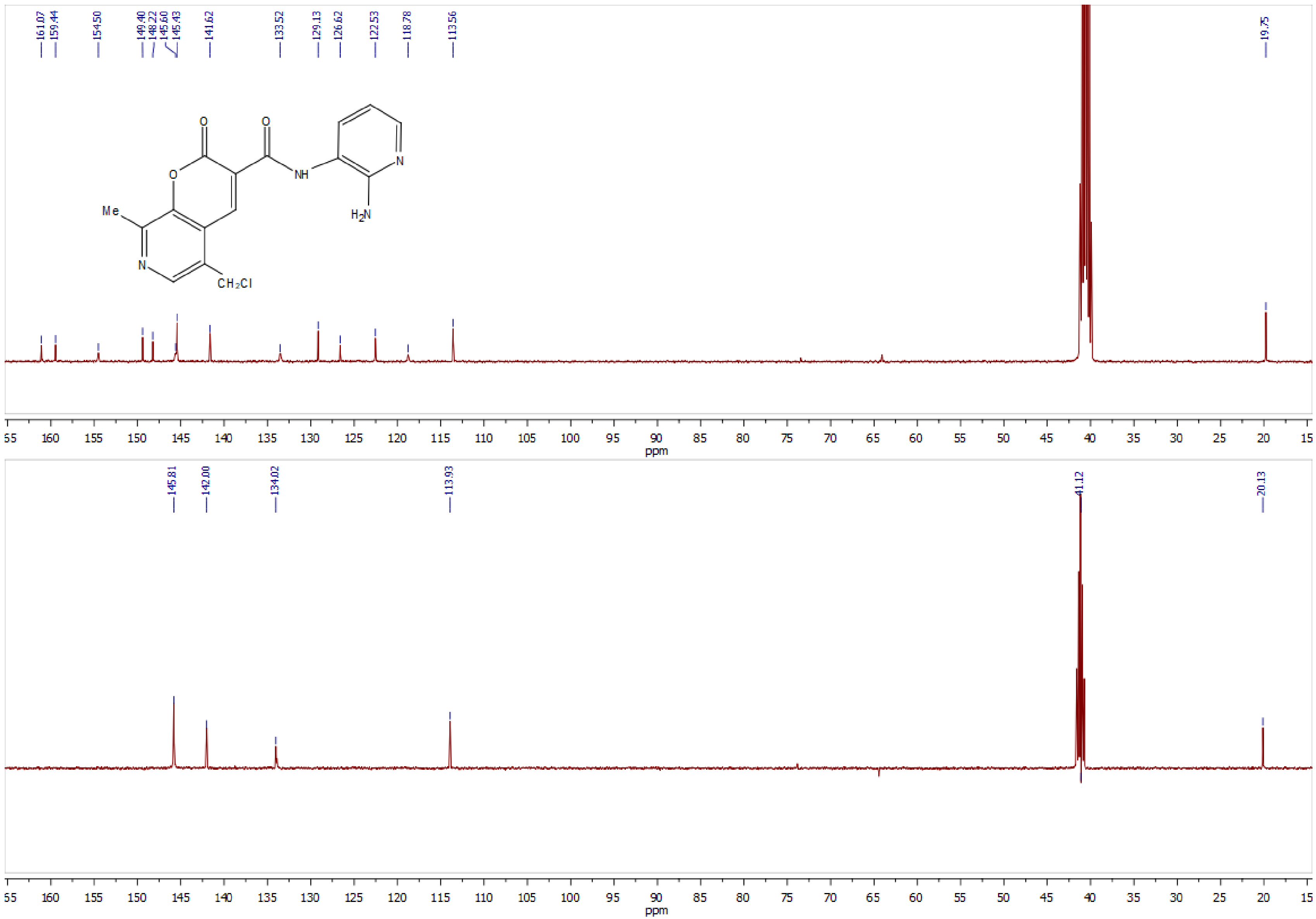

Figure S27. ^{13}C NMR spectra of the compound **7e** (400 MHz, DMSO-d_6)

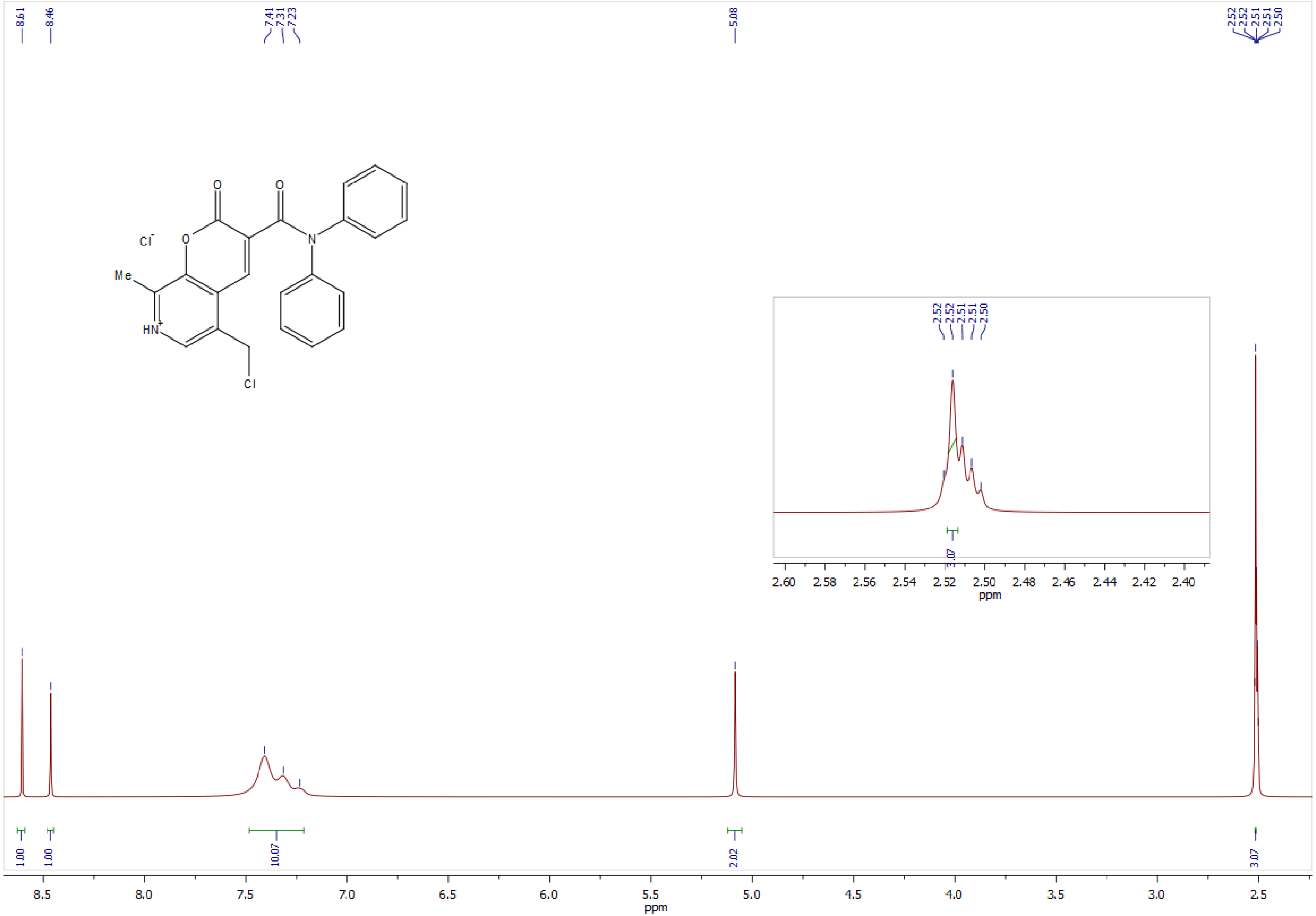

Figure S28. ^{1}H NMR spectra of the compound **7f** (400 MHz, DMSO-d_6)

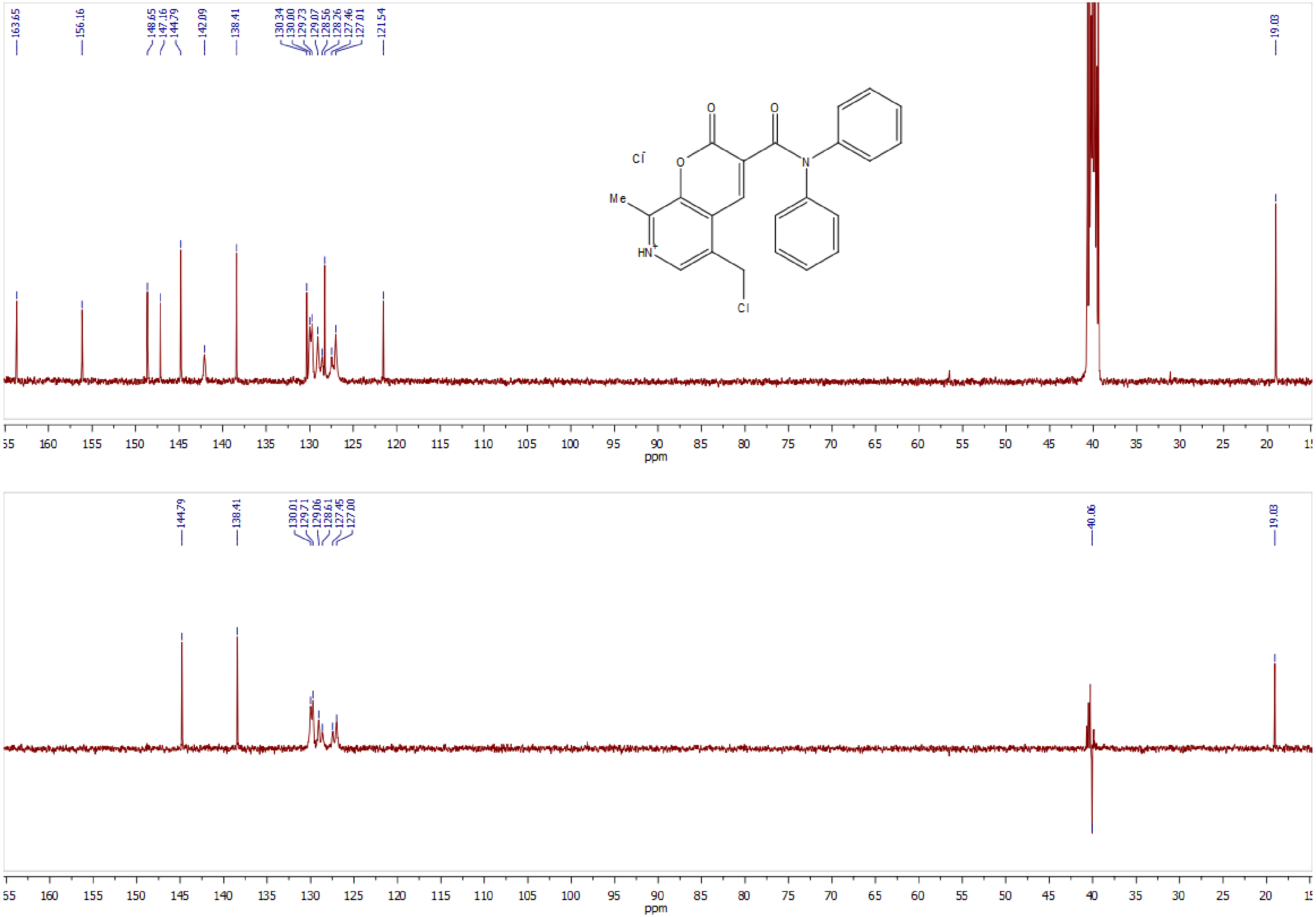

Figure S29. ^{13}C NMR spectra of the compound **7f** (400 MHz, DMSO-d_6)

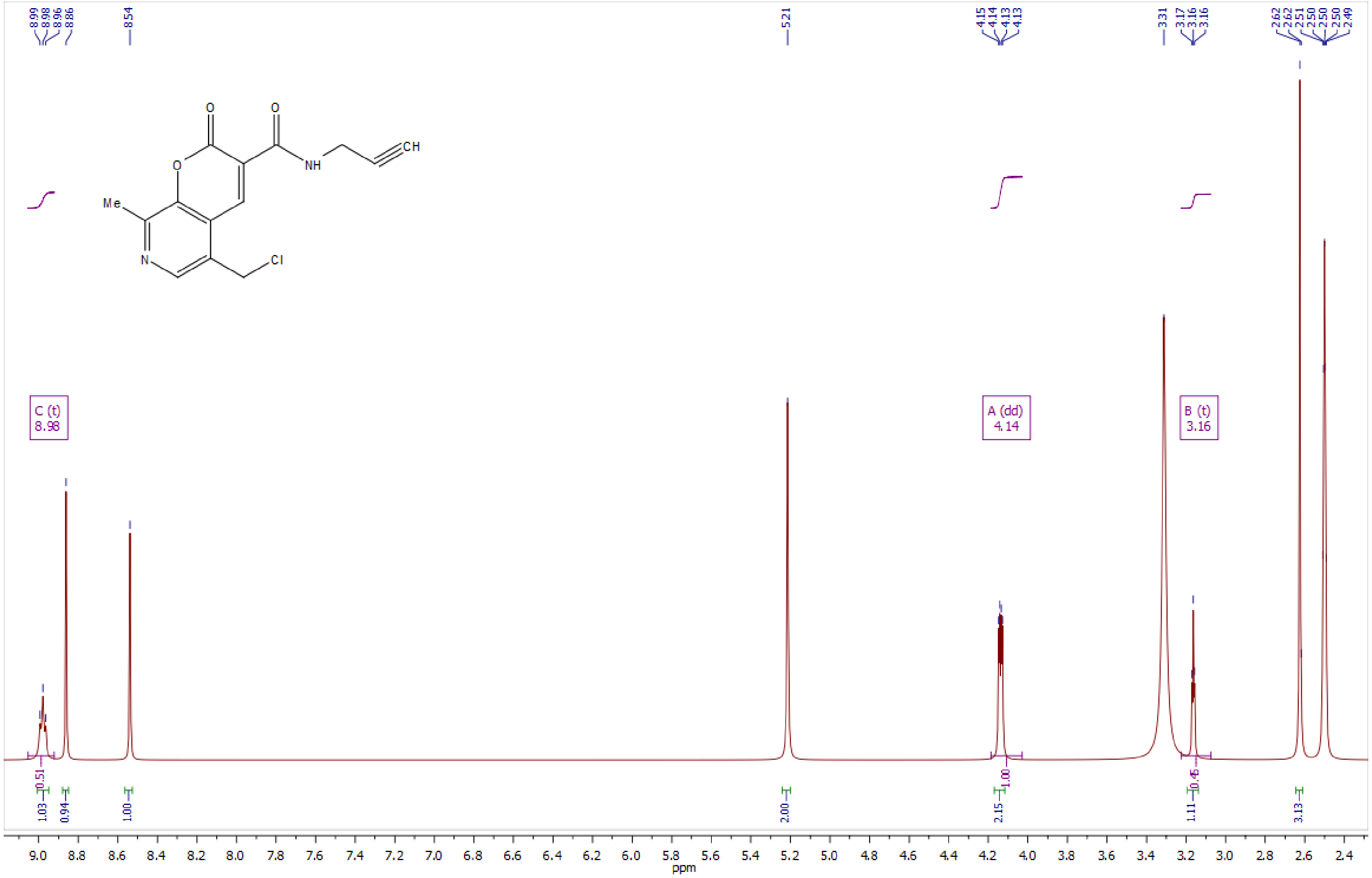

^{1}H NMR (400 MHz, DMSO-d_6) δ 8.98 (t, J = 5.7 Hz, 1H), 4.14 (dd, J = 5.7, 2.5 Hz, 1H), 3.16 (t, J = 2.5 Hz, 0H).

Figure S30. ^{1}H NMR spectra of the compound **7g** (400 MHz, DMSO-d_6)

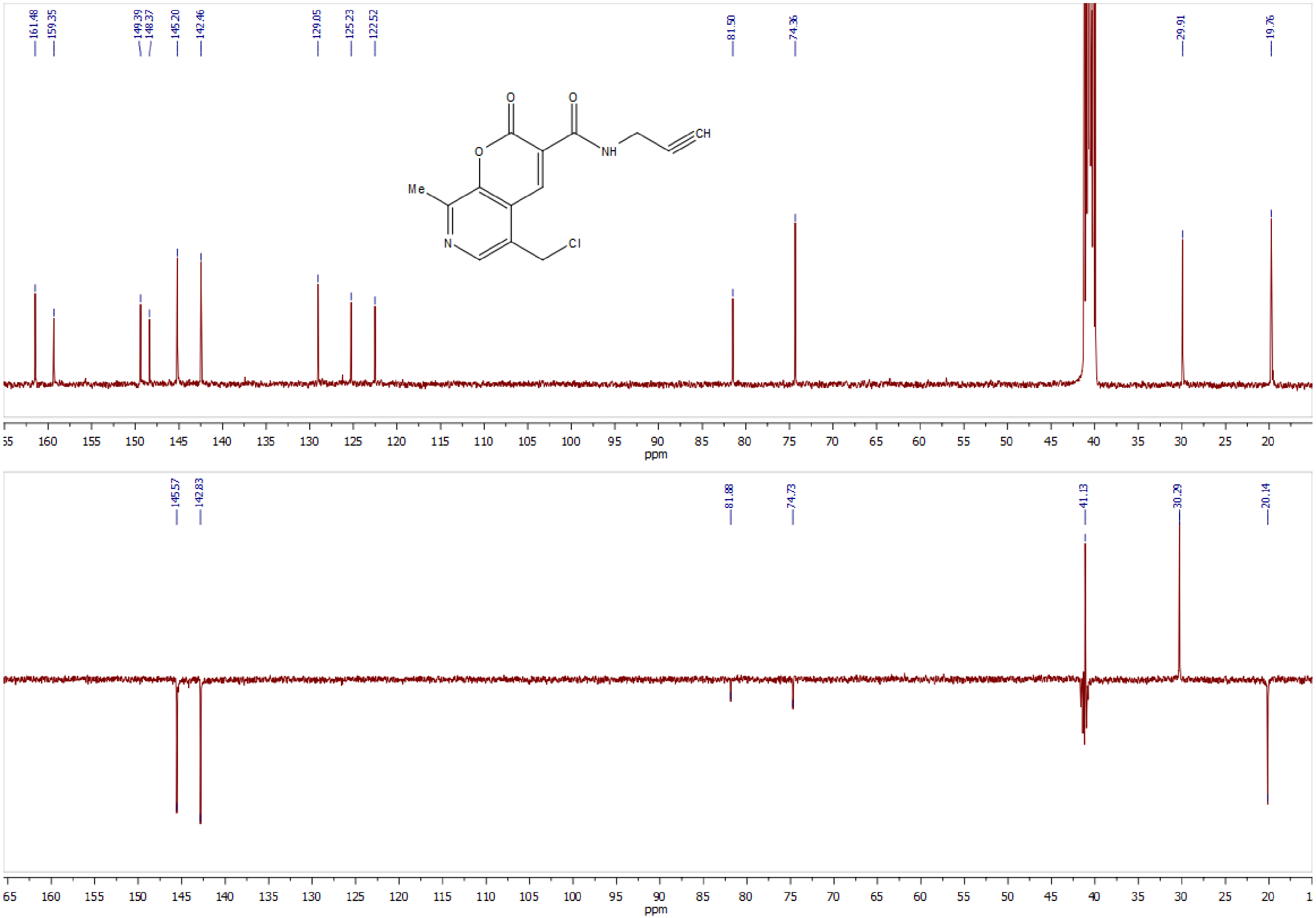

Figure S31. ^{13}C NMR spectra of the compound **7g** (400 MHz, DMSO-d_6)

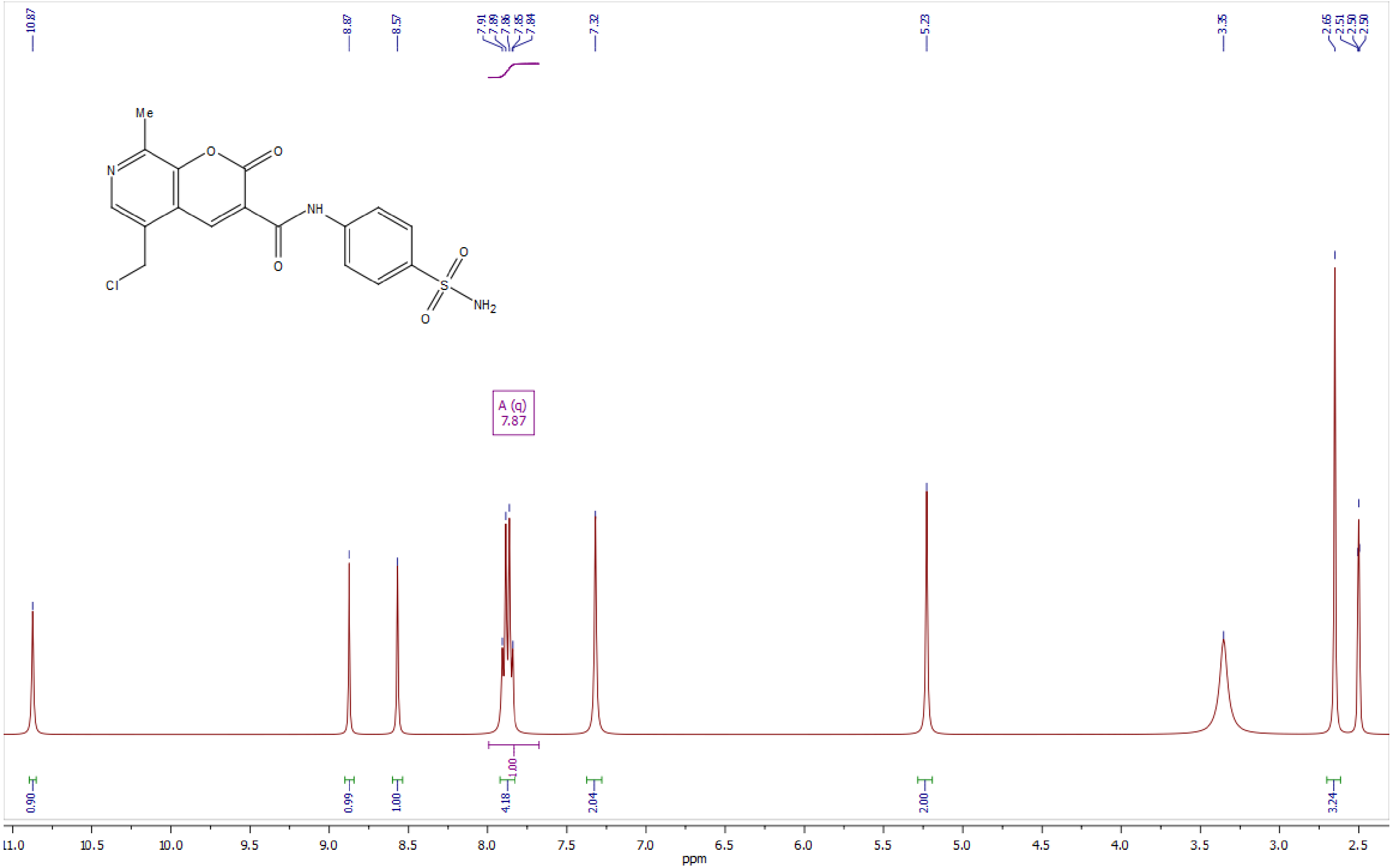

^{1}H NMR (400 MHz, DMSO-d_6) δ 7.87 (q, J = 8.8 Hz, 1H).

Figure S32. ^{1}H NMR spectra of the compound **7h** (400 MHz, DMSO-d_6)

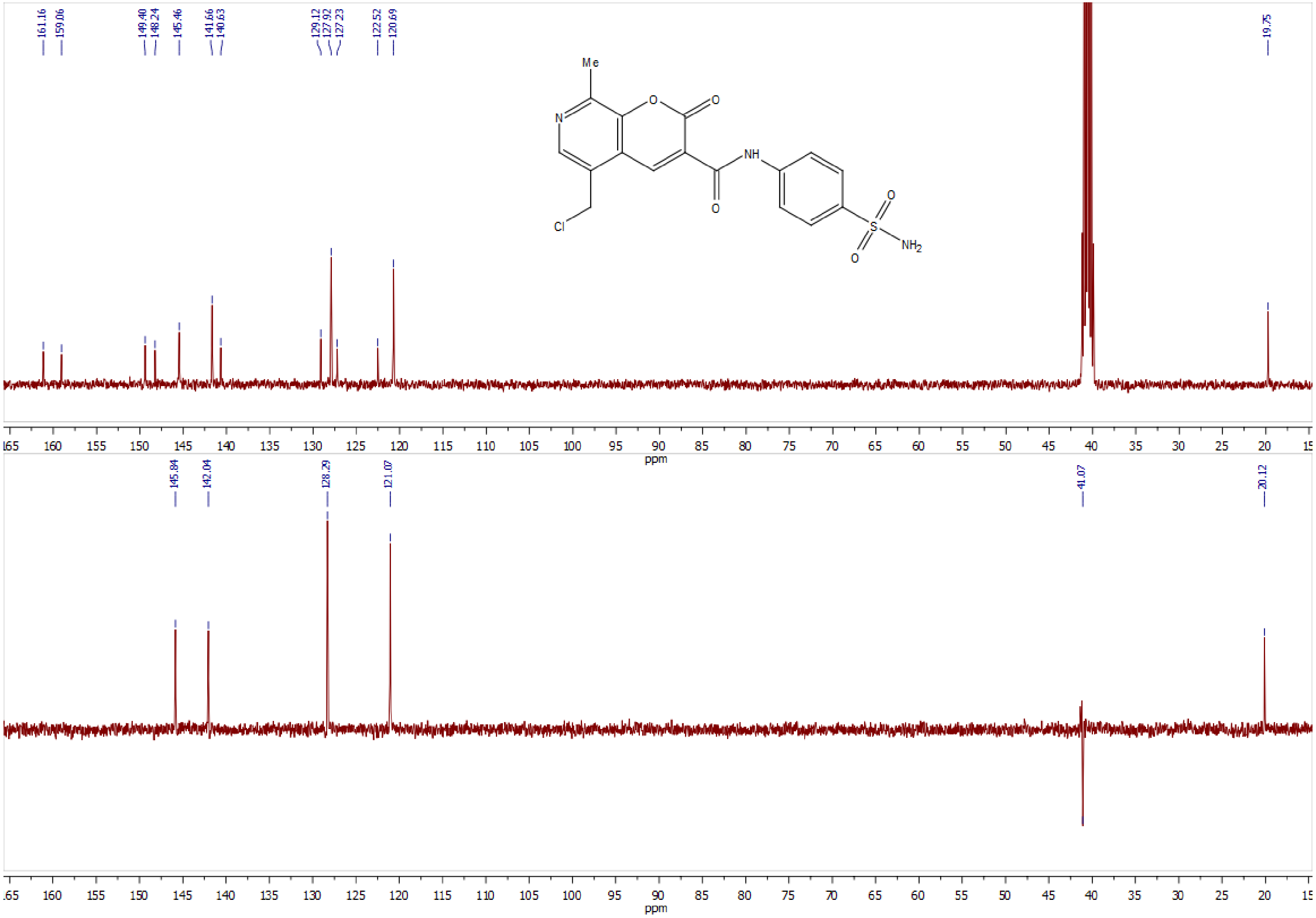

Figure S33. ^{13}C NMR spectra of the compound **7h** (400 MHz, DMSO-d_6)

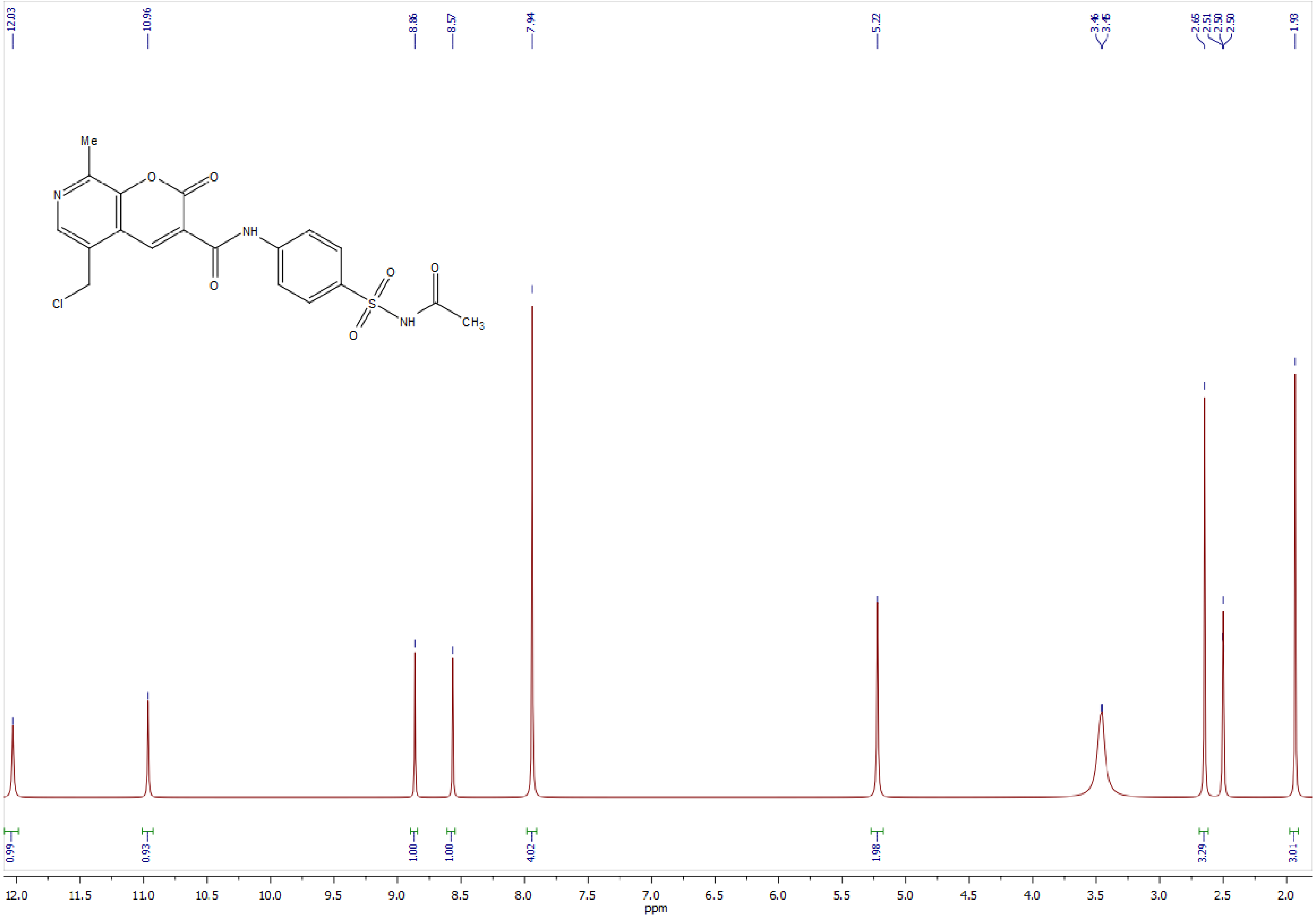

Figure S34. ^{1}H NMR spectra of the compound **7i** (400 MHz, DMSO-d_6)

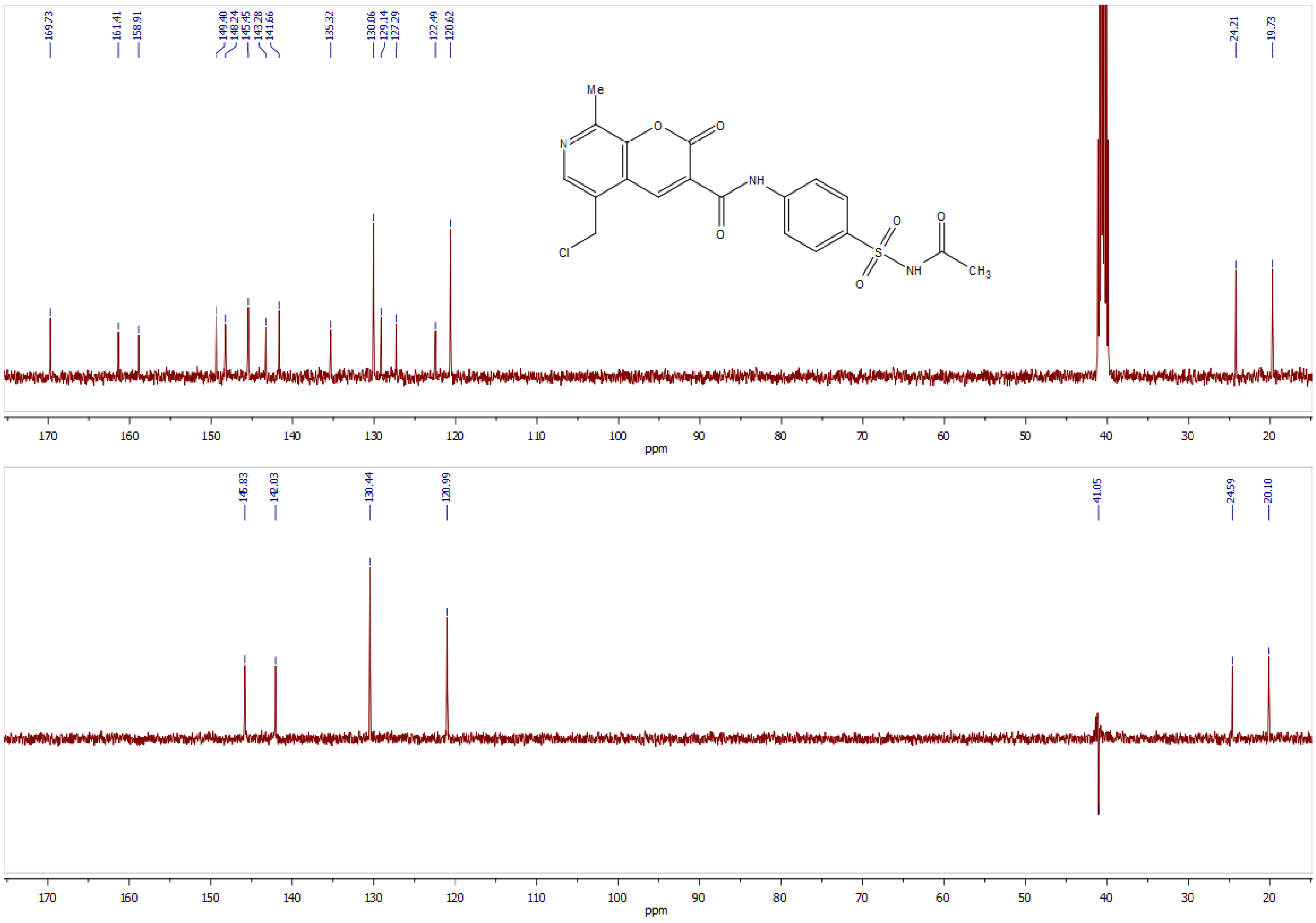

Figure S35. ^{13}C NMR spectra of the compound **7i** (400 MHz, DMSO-d_6)

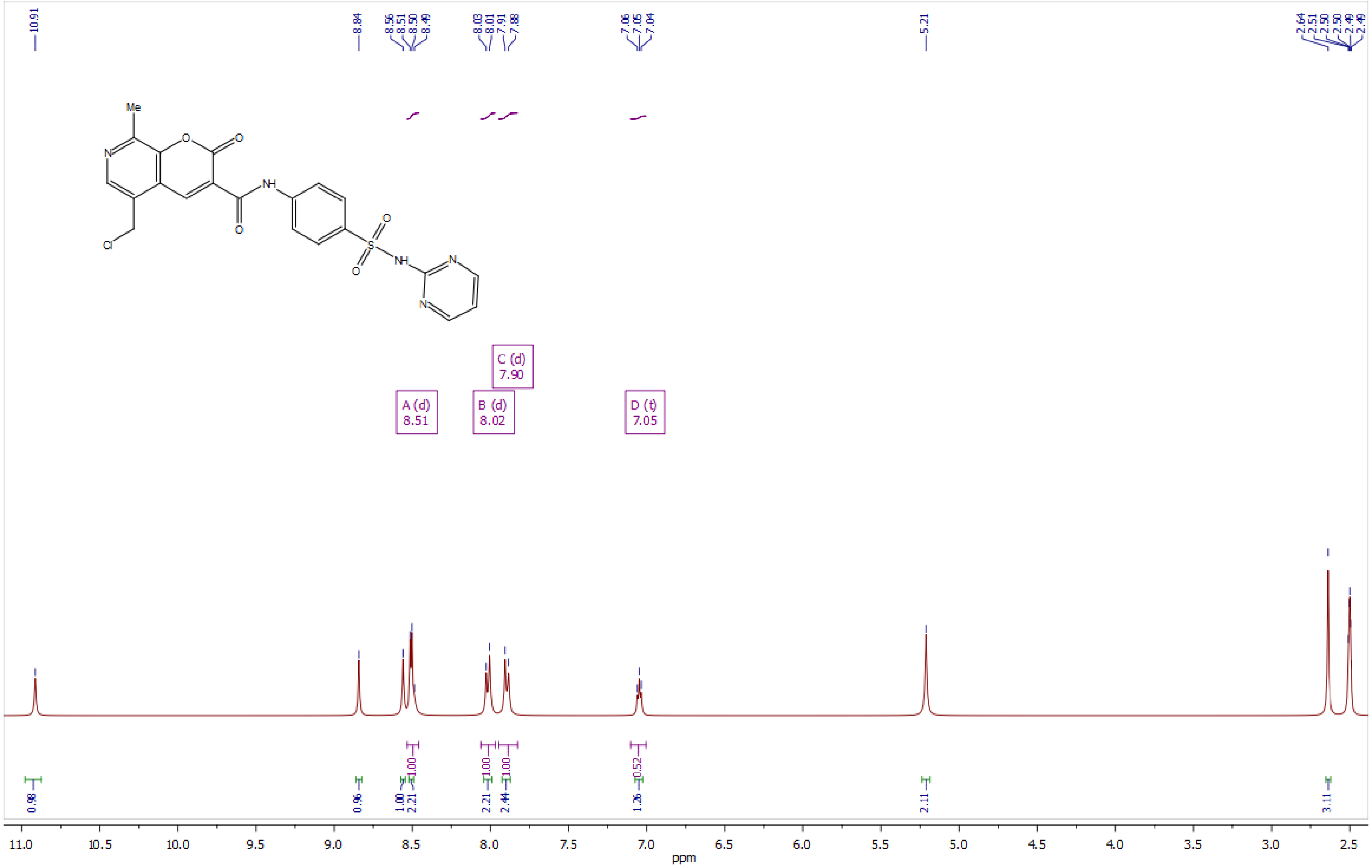

^{1}H NMR (400 MHz, DMSO-d_6) δ 8.51 (d, J = 4.9 Hz, 1H), 8.02 (d, J = 8.6 Hz, 1H), 7.90 (d, J = 8.6 Hz, 1H), 7.05 (t, J = 4.9 Hz, 1H).

Figure S36. ^{1}H NMR spectra of the compound **7j** (400 MHz, DMSO-d_6)

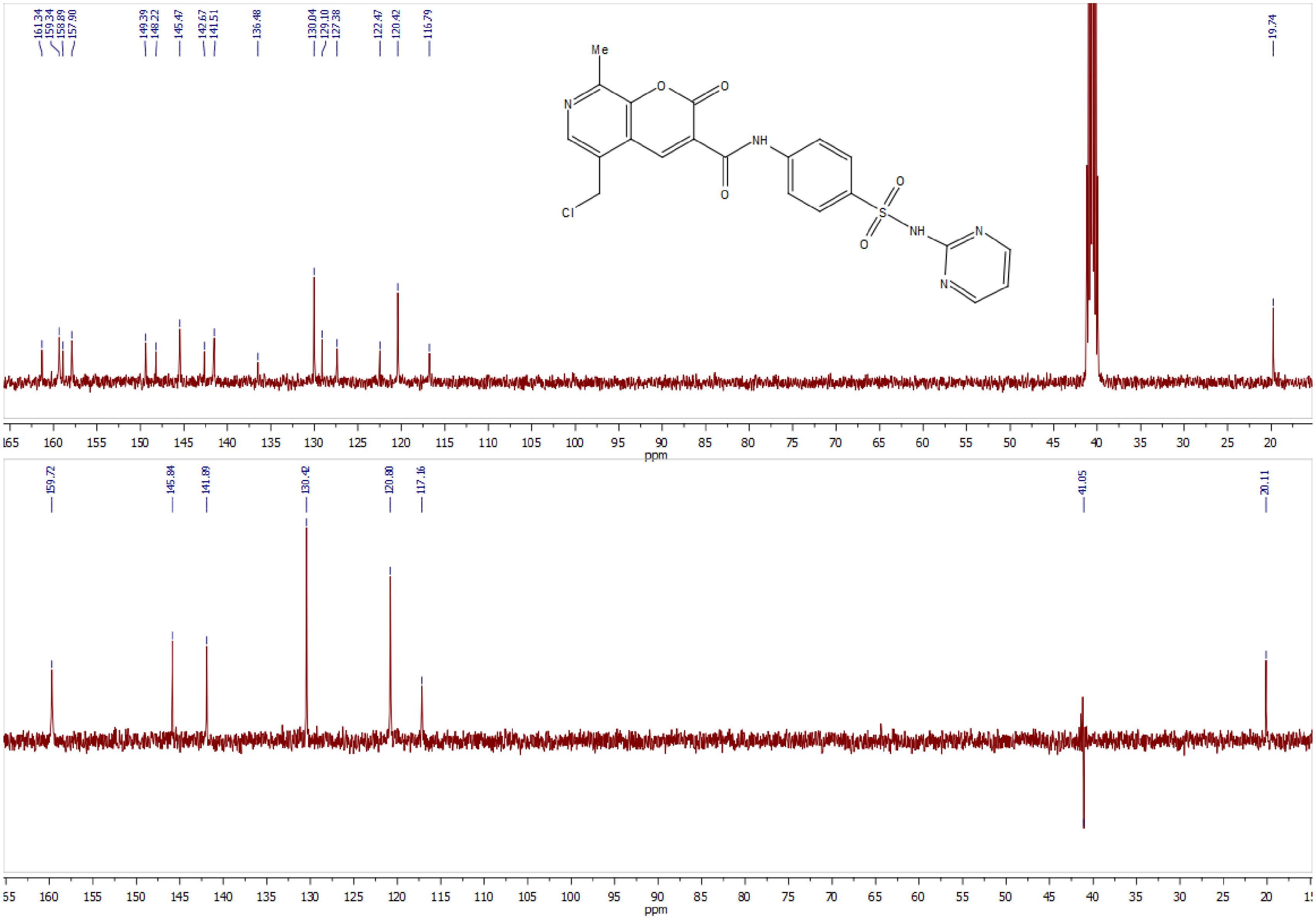

Figure S37. ^{13}C NMR spectra of the compound **7j** (400 MHz, DMSO-d_6)

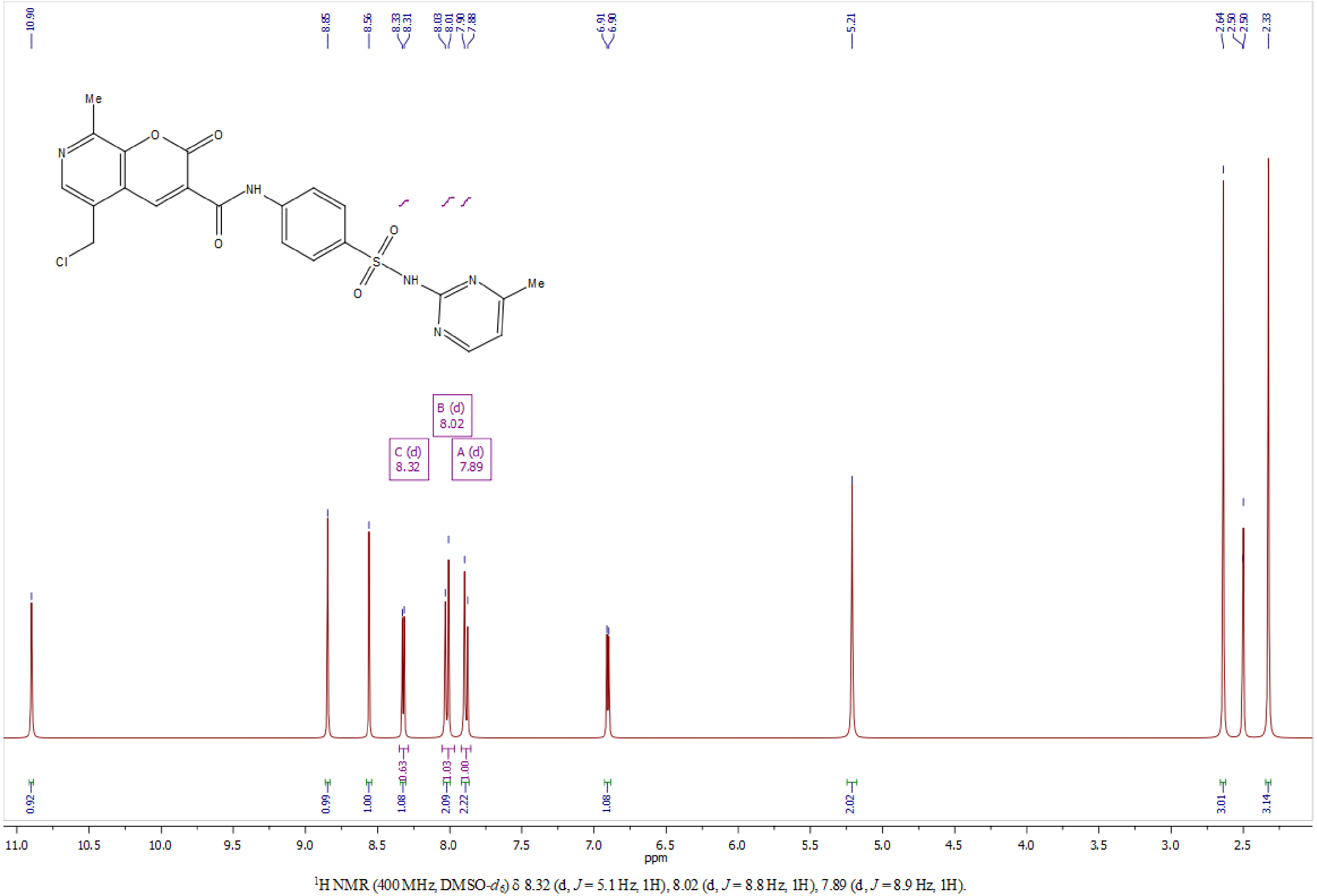

Figure S38. ^{1}H NMR spectra of the compound **7k** (400 MHz, DMSO-d_6)

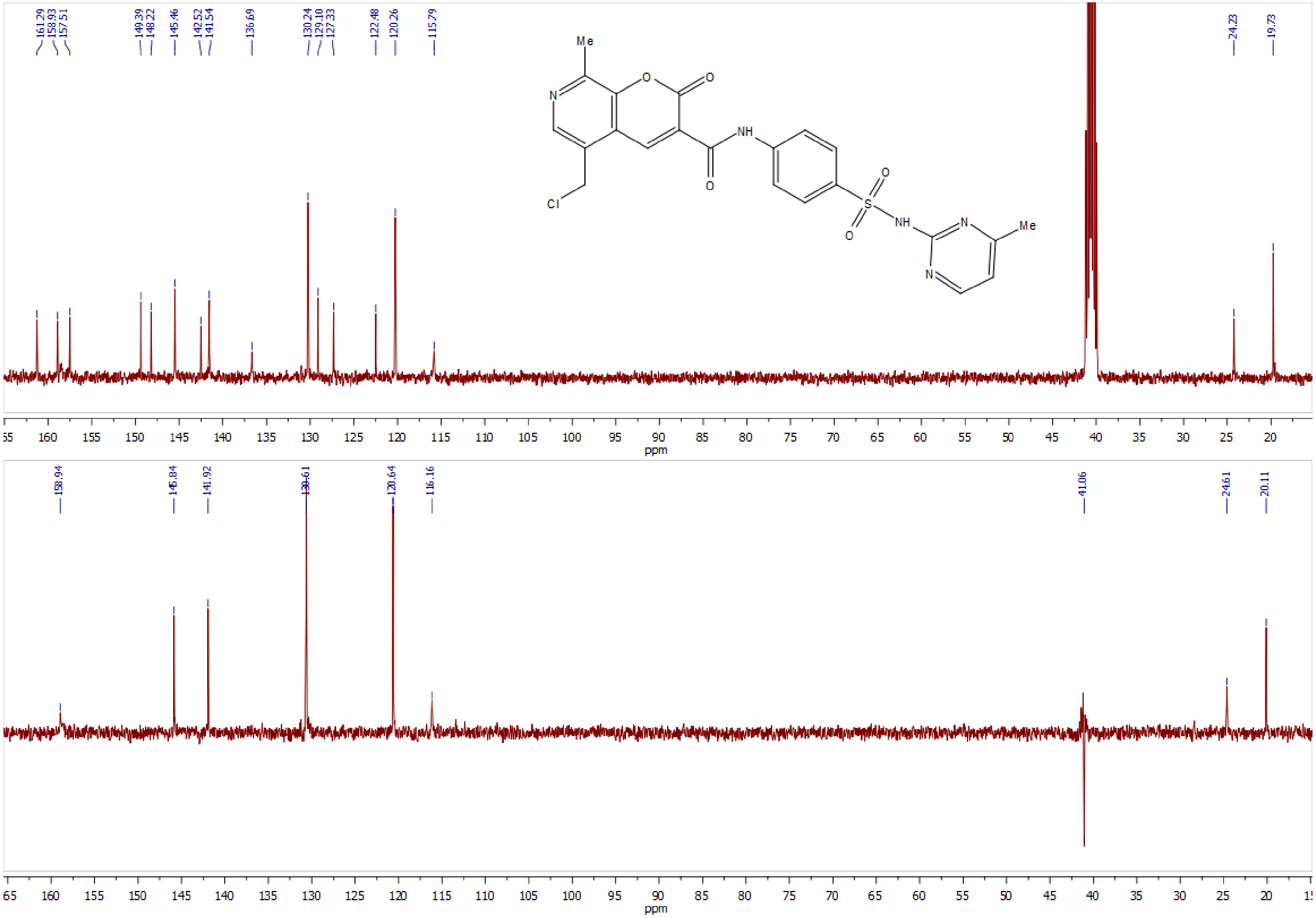

Figure S39. ^{13}C NMR spectra of the compound **7k** (400 MHz, DMSO-d_6)

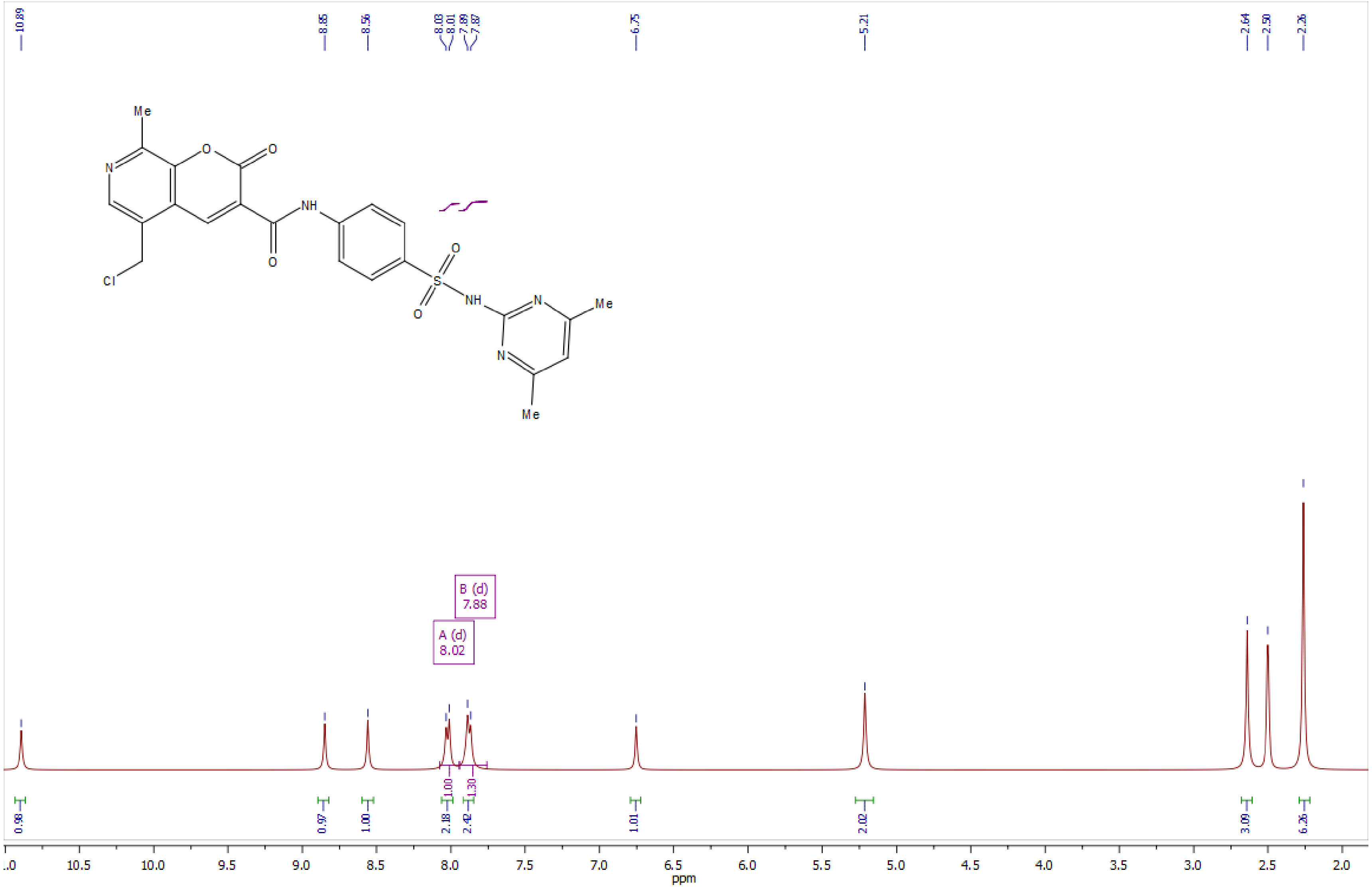

^{1}H NMR (400 MHz, DMSO-d_6) δ 8.02 (d, J = 8.5 Hz, 1H), 7.88 (d, J = 8.7 Hz, 1H).

Figure S40. ^{1}H NMR spectra of the compound **7l** (400 MHz, DMSO-d_6)

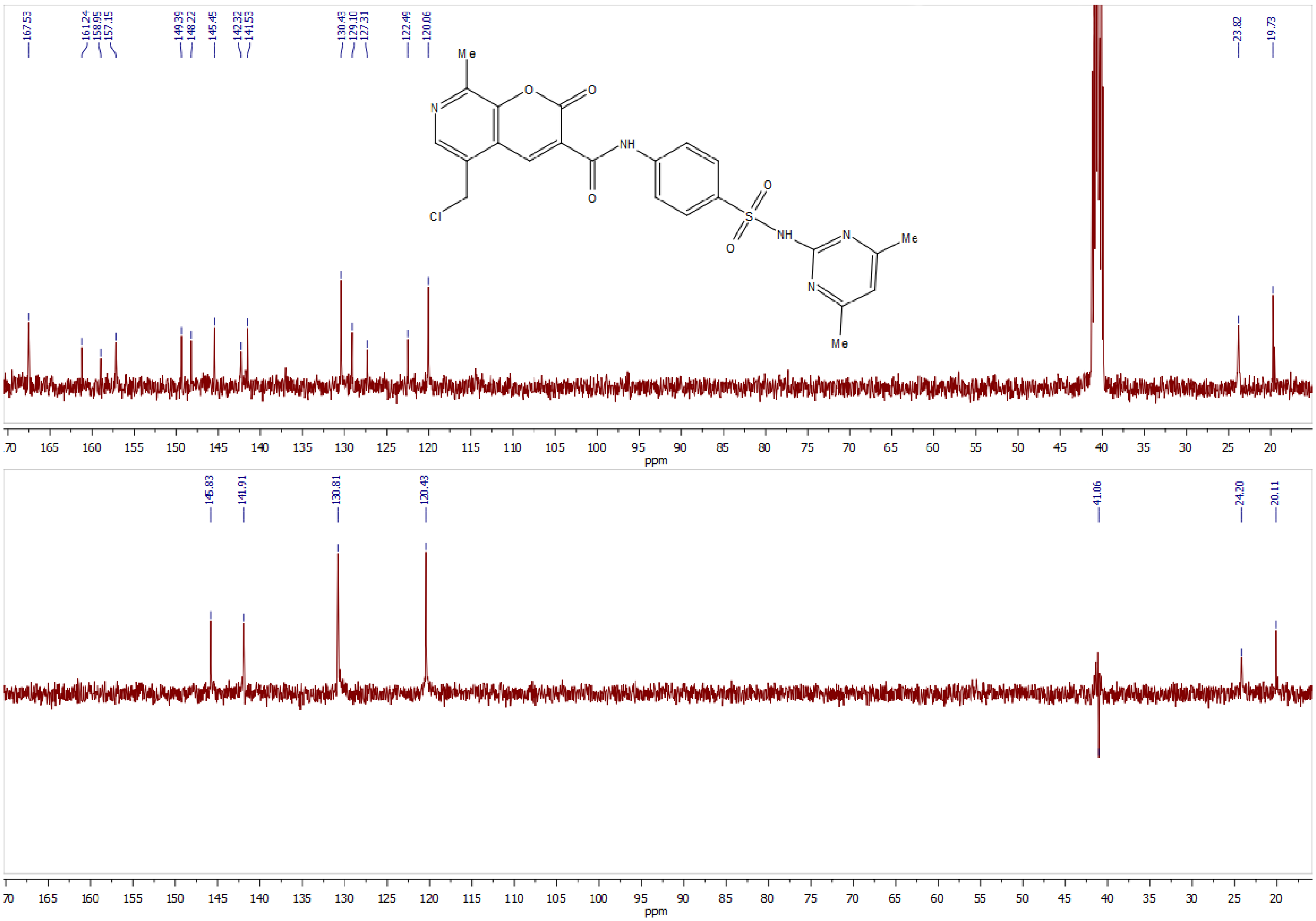

Figure S41. ^{13}C NMR spectra of the compound **7l** (400 MHz, DMSO-d_6)

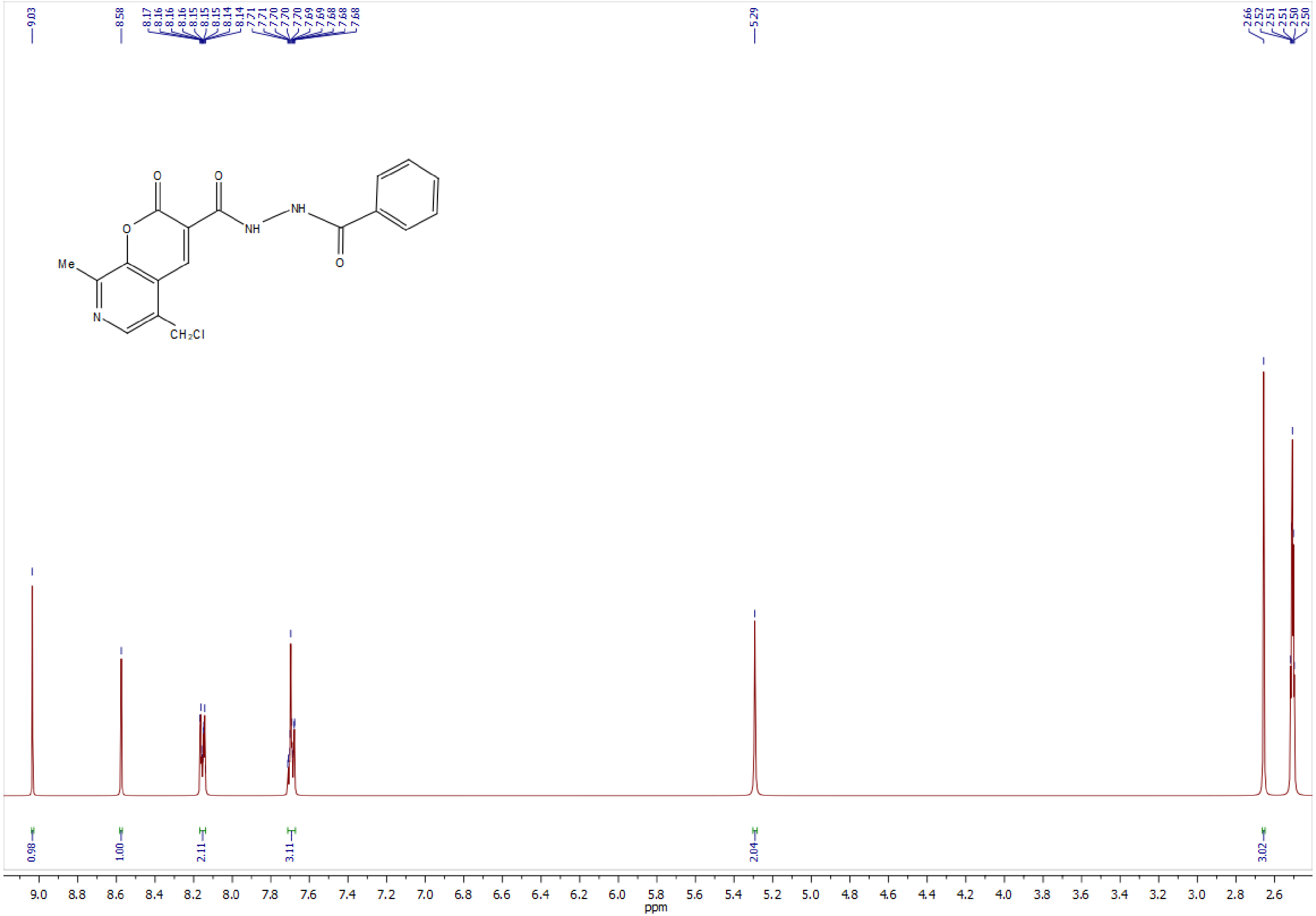

Figure S42. ^{1}H NMR spectra of the compound **7m** (400 MHz, DMSO-d_6)

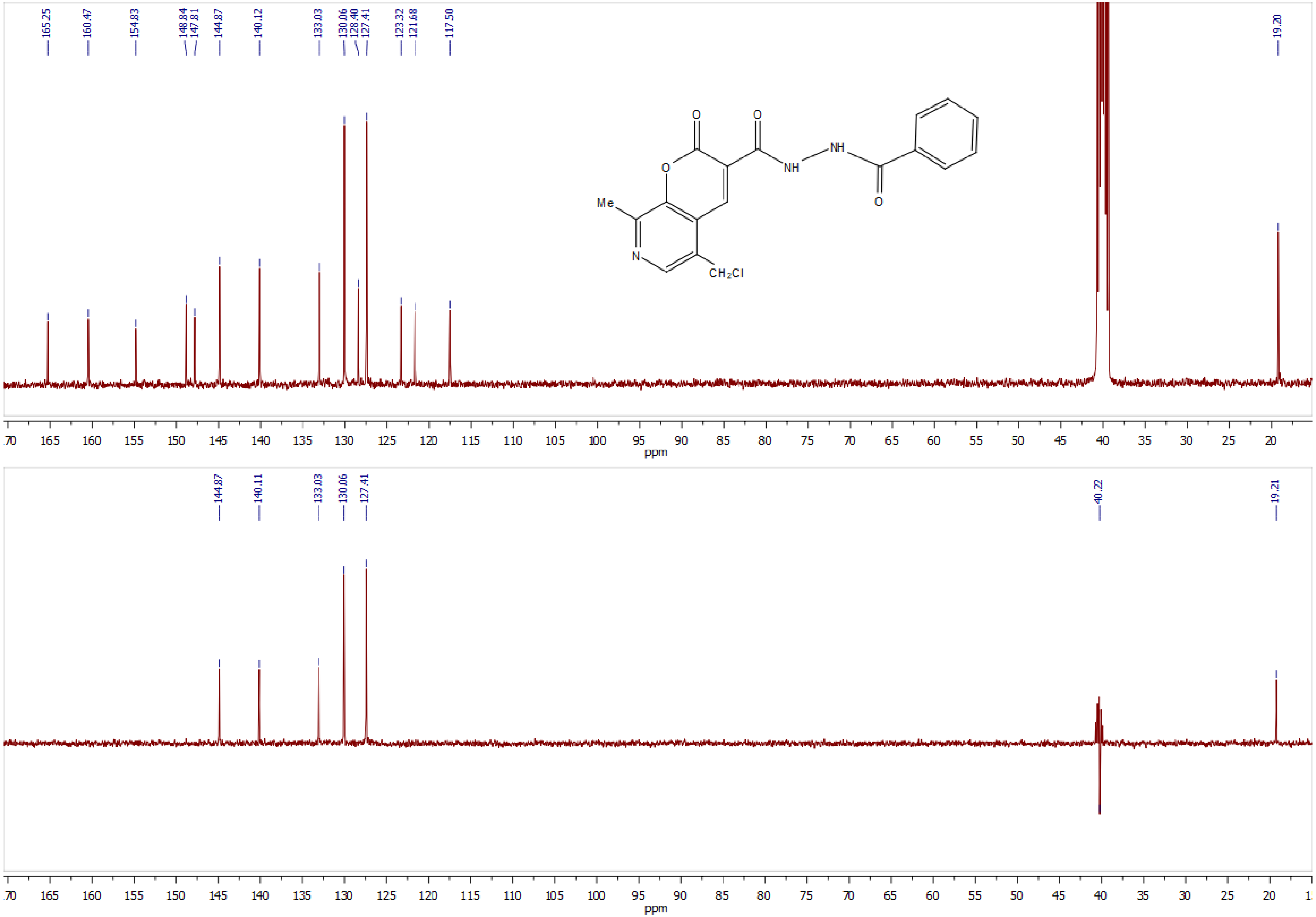

Figure S43. ^{13}C NMR spectra of the compound **7m** (400 MHz, DMSO-d_6)

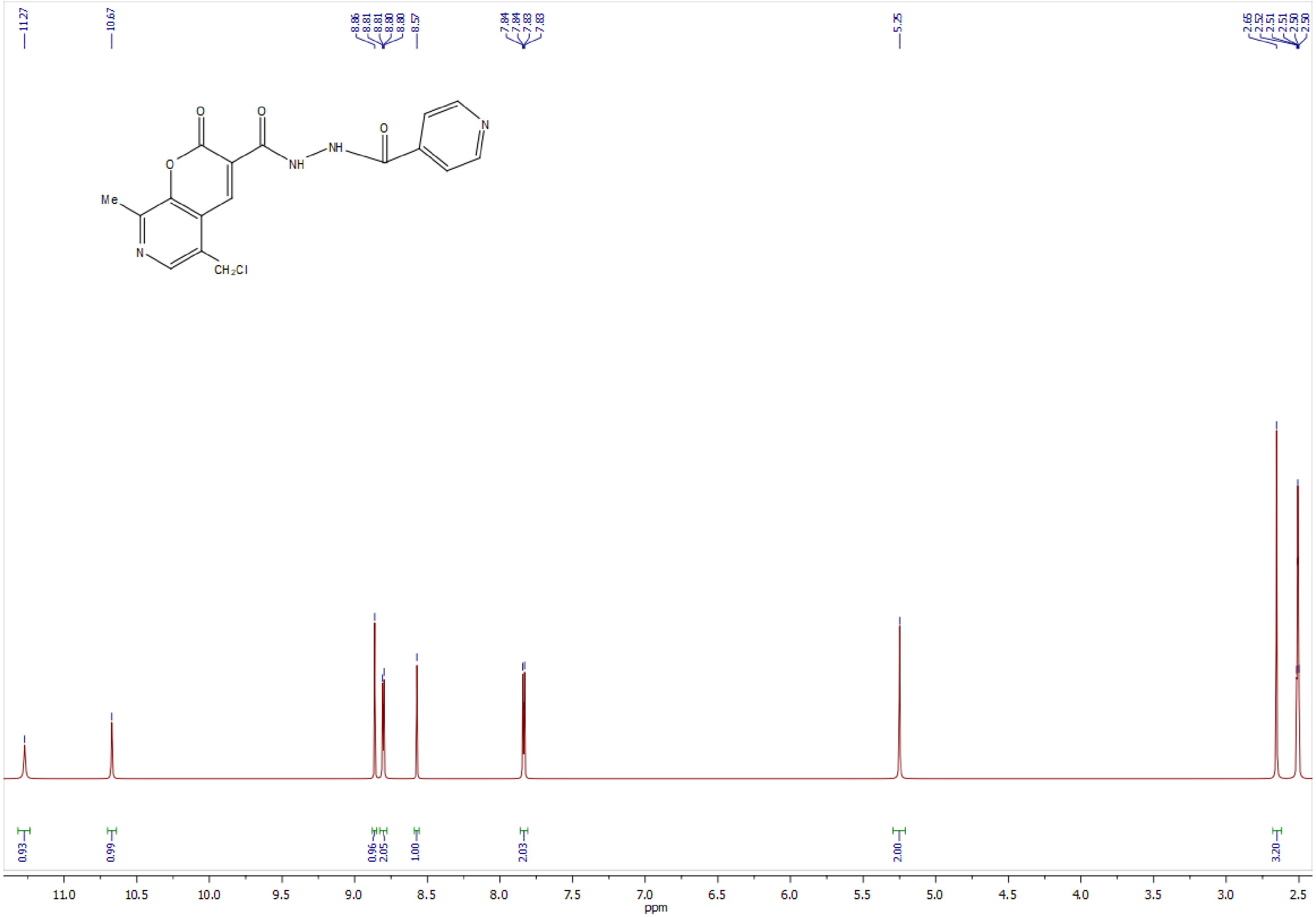

Figure S44. ^{1}H NMR spectra of the compound **7n** (400 MHz, DMSO-d_6)

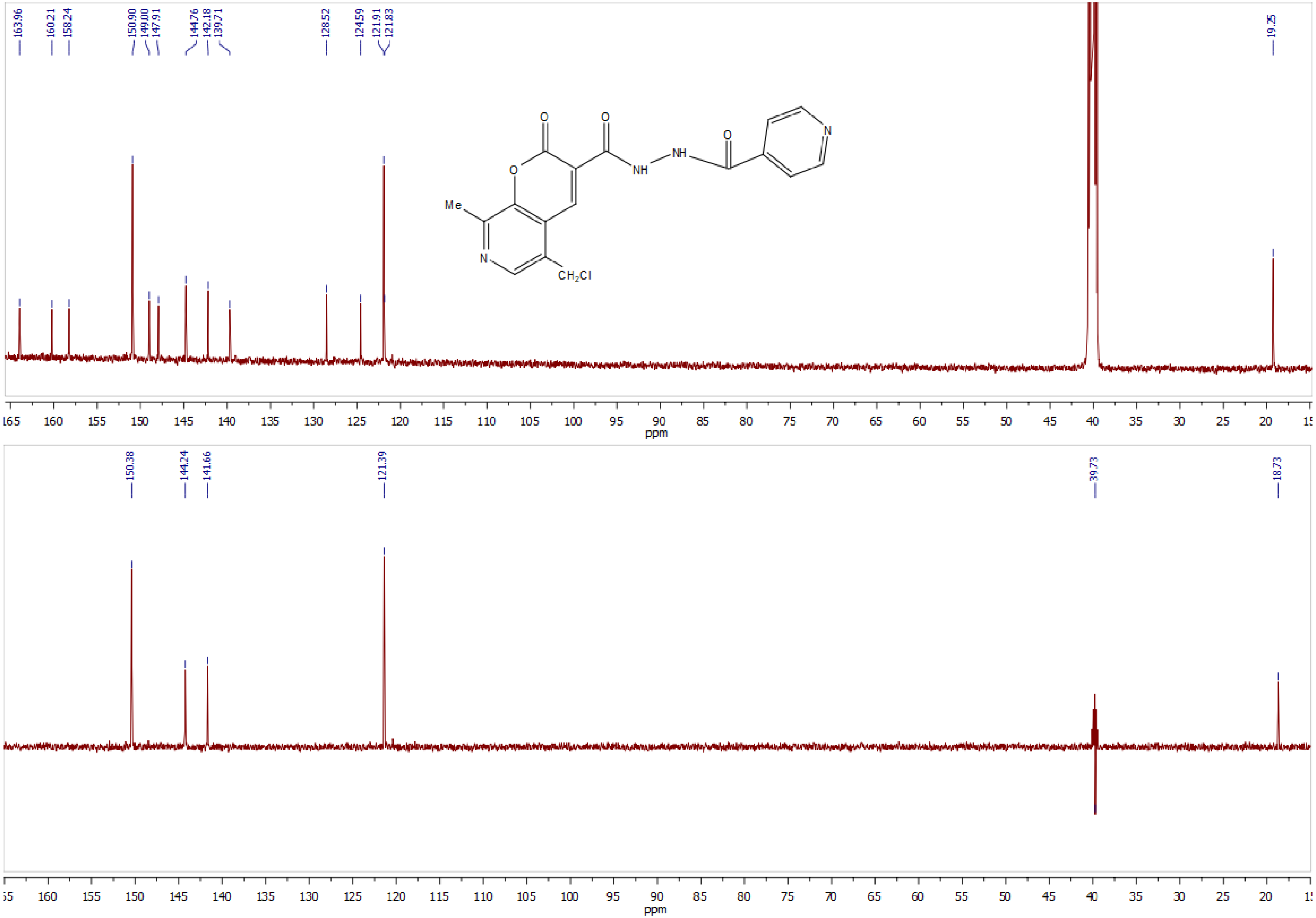

Figure S45. ^{13}C NMR spectra of the compound **7n** (400 MHz, DMSO-d_6)

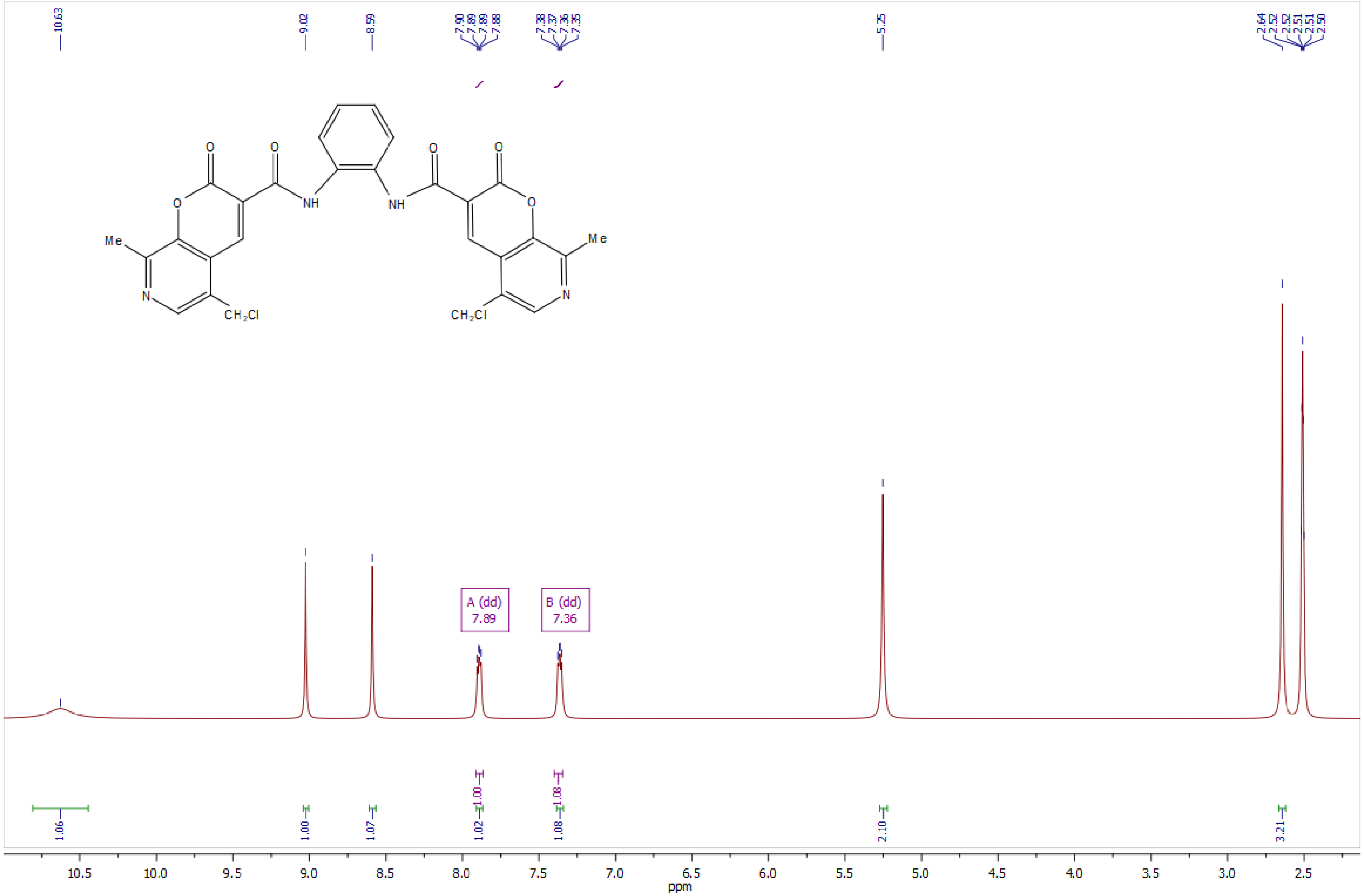

^{1}H NMR (400 MHz, DMSO-d_6) δ 7.89 (dd, J = 6.0, 3.5 Hz, 1H), 7.36 (dd, J = 6.1, 3.5 Hz, 1H).

Figure S46. ^{1}H NMR spectra of the compound **7o** (400 MHz, DMSO-d_6)

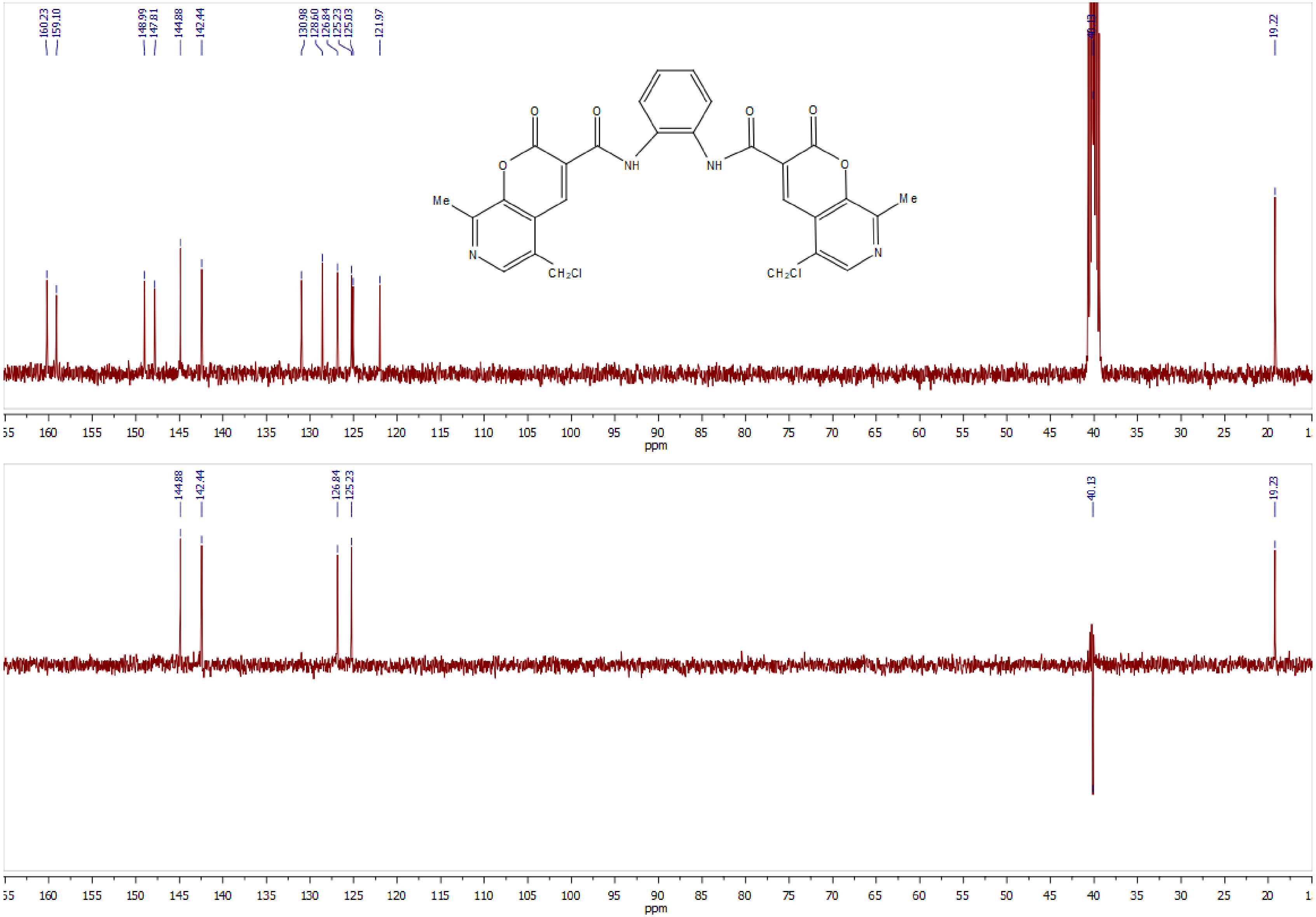

Figure S47. ^{13}C NMR spectra of the compound **7o** (400 MHz, DMSO-d_6)

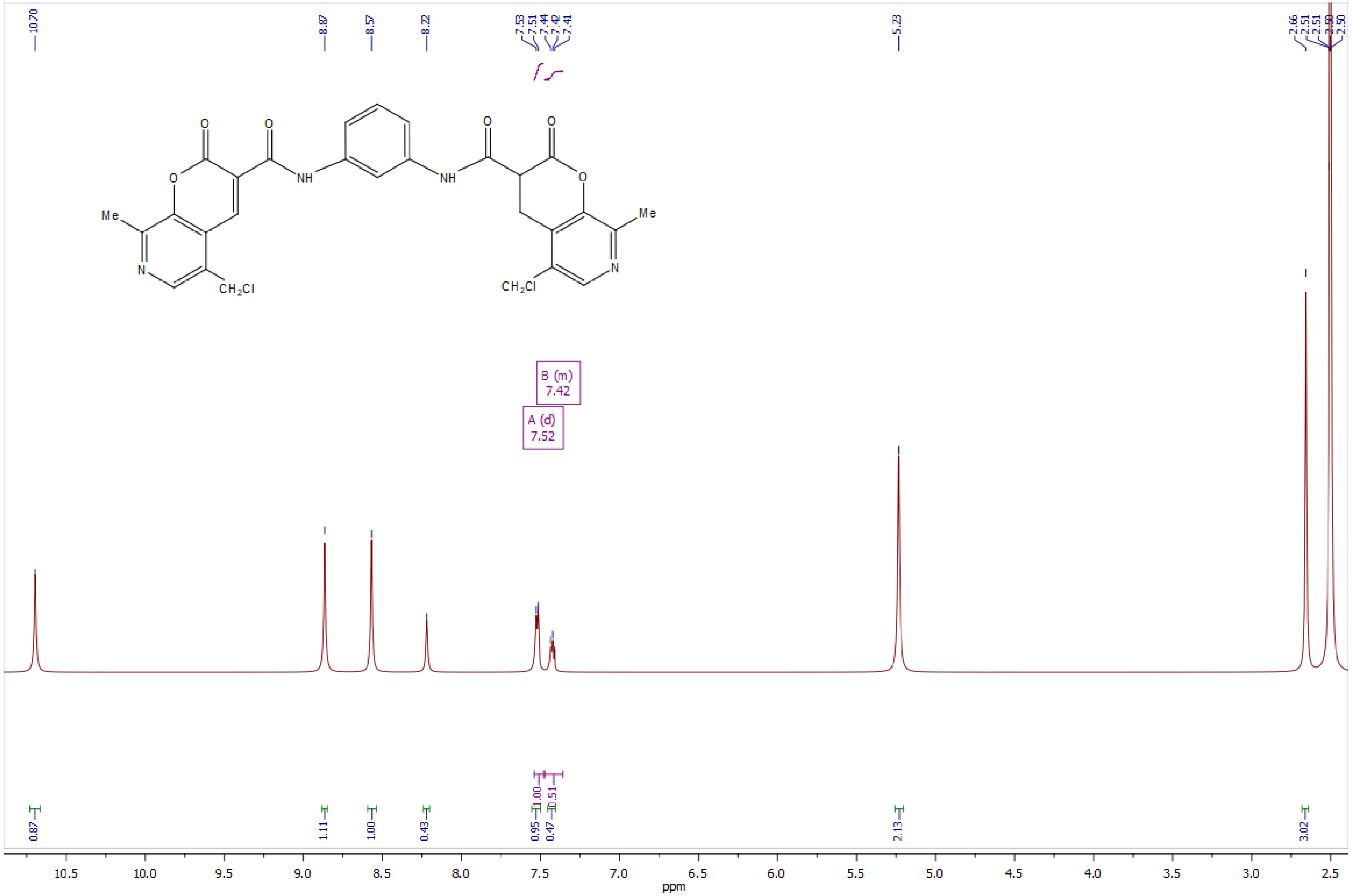

^{1}H NMR (600 MHz, DMSO-d_6) δ 7.52 (d, J = 8.0 Hz, 1H), 7.44 – 7.40 (m, 1H).

Figure S48. ^{1}H NMR spectra of the compound **7p** (600 MHz, DMSO-d_6)

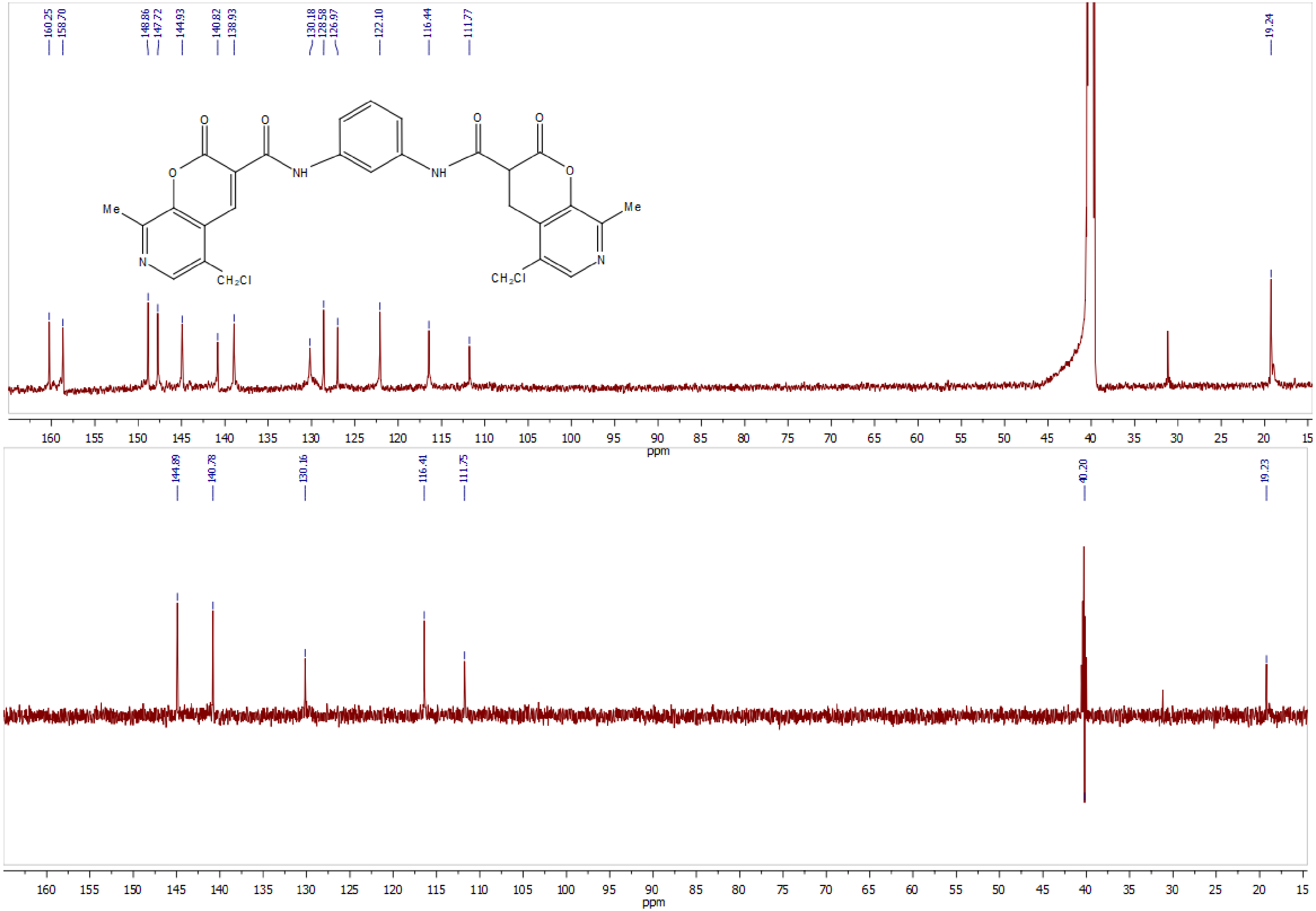

Figure S49. ^{13}C NMR spectra of the compound **7p** (600 MHz, DMSO-d_6)

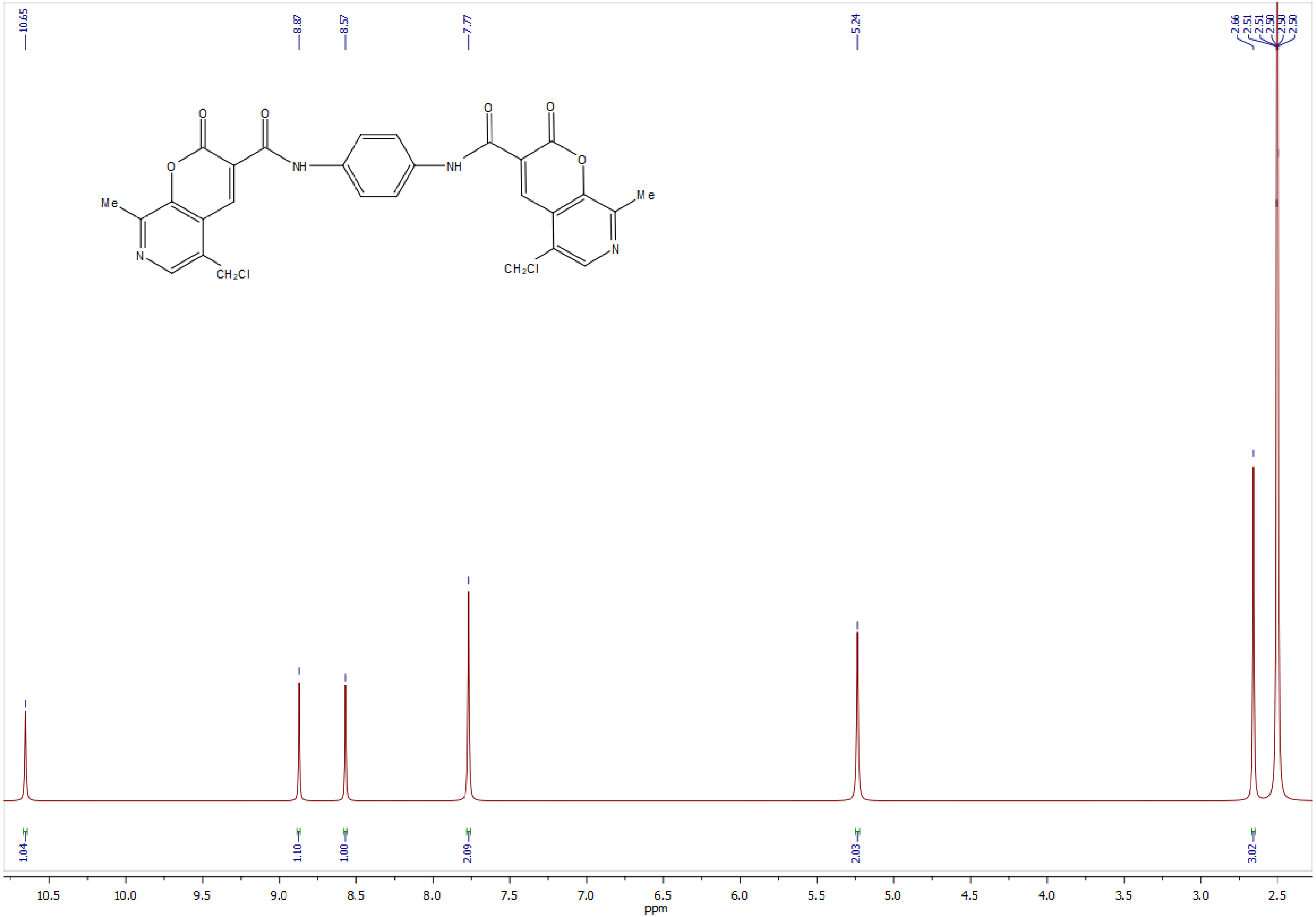

Figure S50. ^{1}H NMR spectra of the compound **7q** (600 MHz, DMSO-d_6)

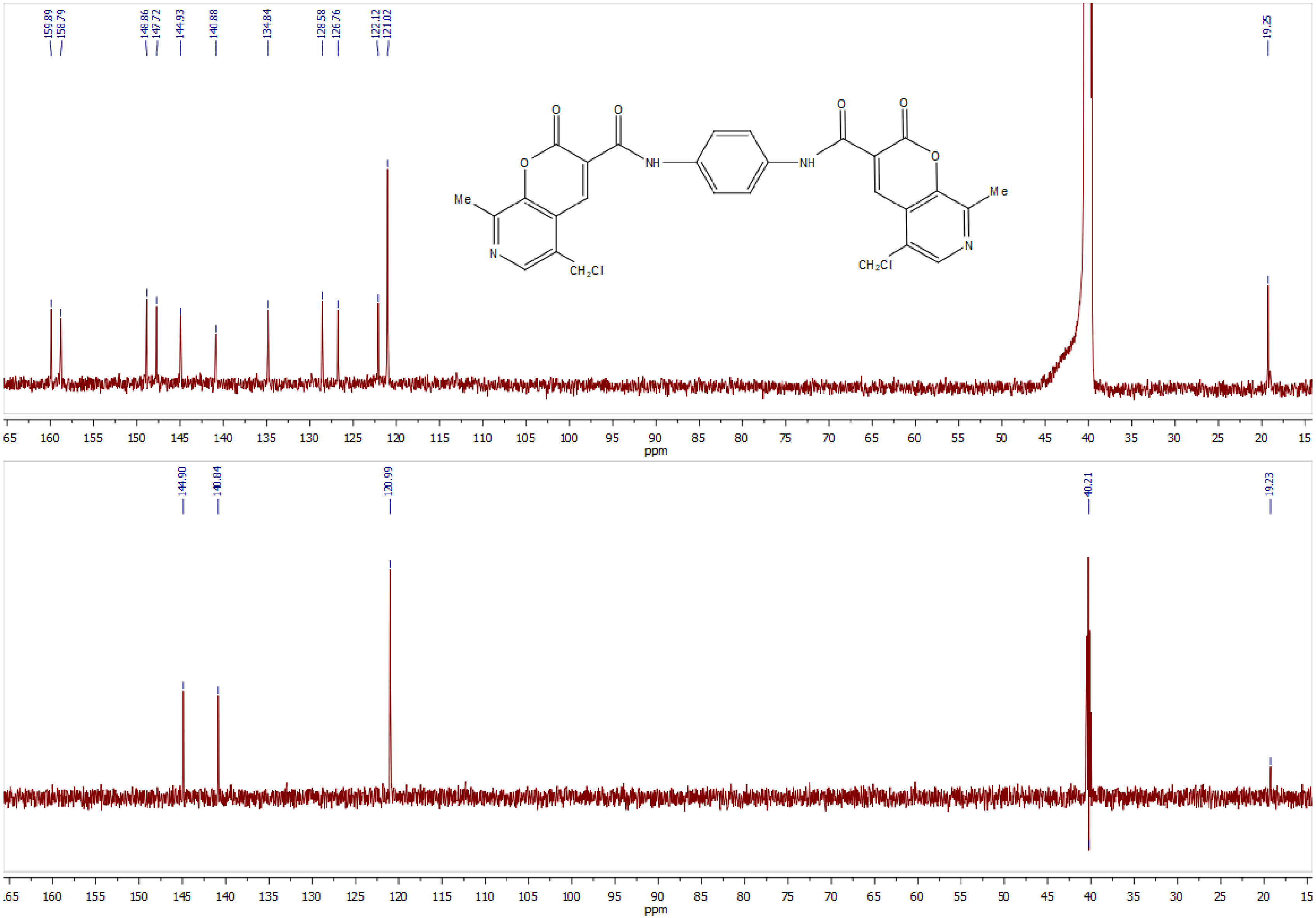

Figure S51. ^{13}C NMR spectra of the compound **7q** (600 MHz, DMSO-d_6)

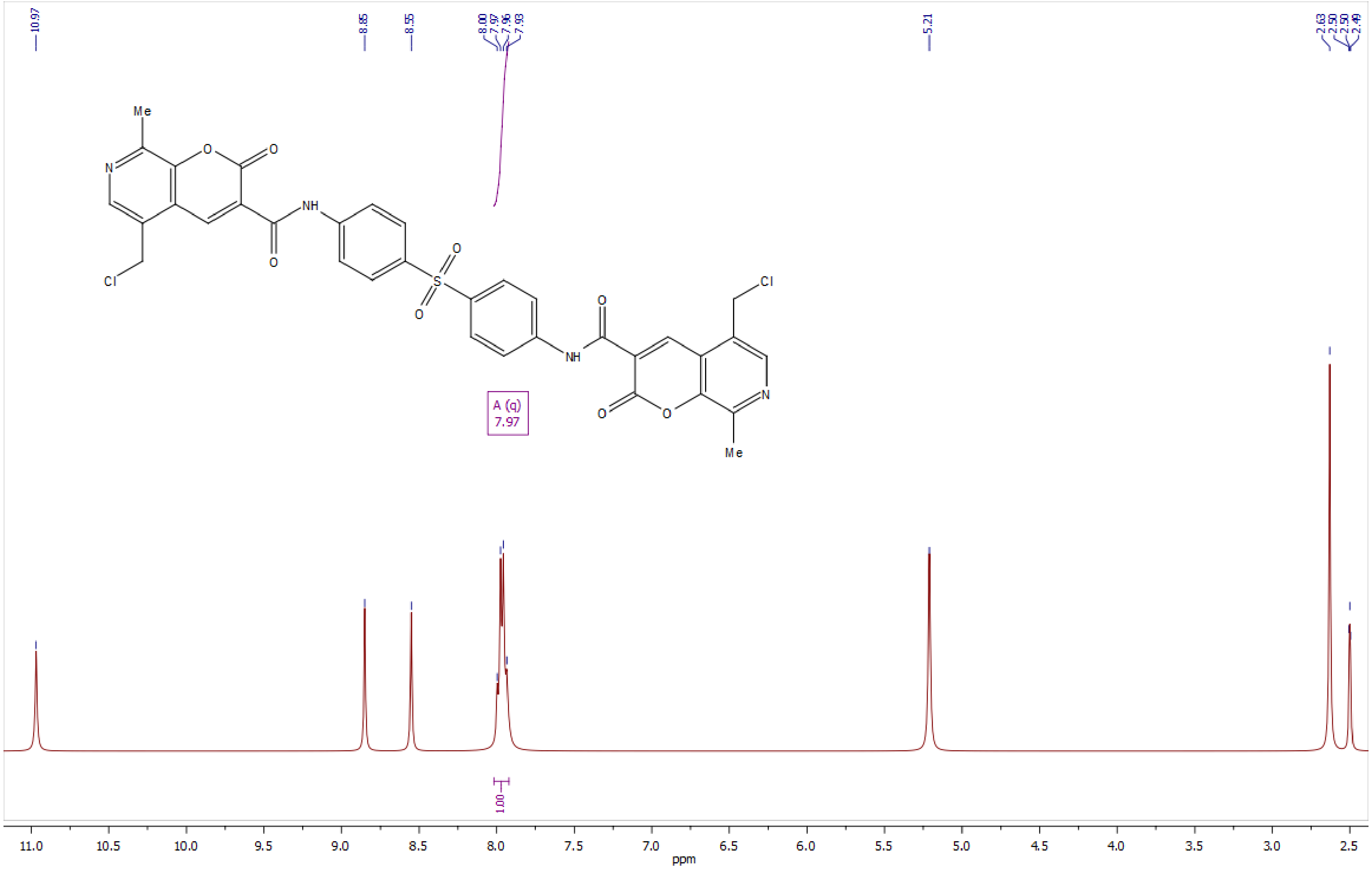

^{1}H NMR (400 MHz, DMSO-d_6) δ 7.97 (q, J = 8.7 Hz, 1H).

Figure S52. ^{1}H NMR spectra of the compound **7r** (400 MHz, DMSO-d_6)

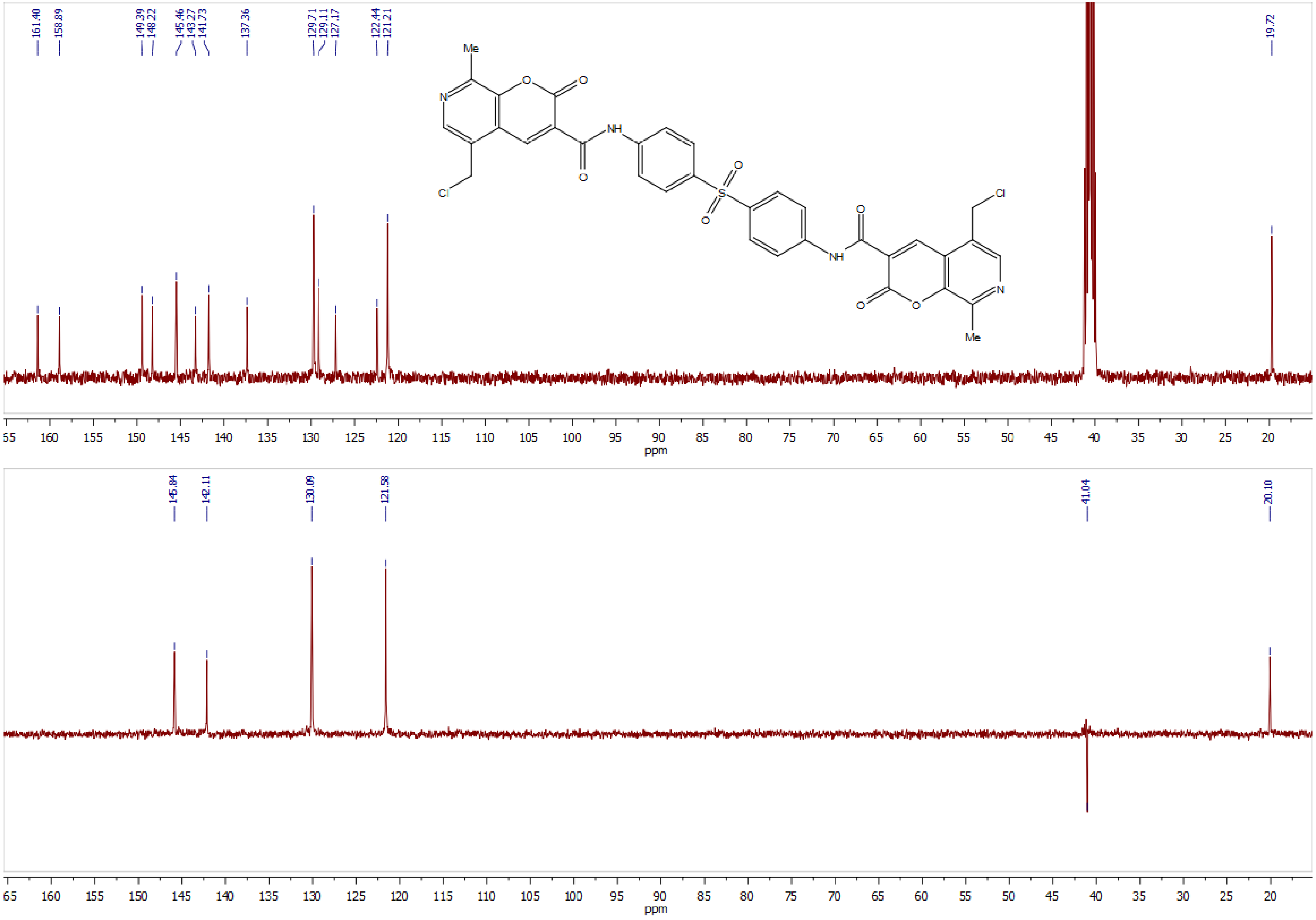

Figure S53. ^{13}C NMR spectra of the compound **7r** (400 MHz, DMSO-d_6)

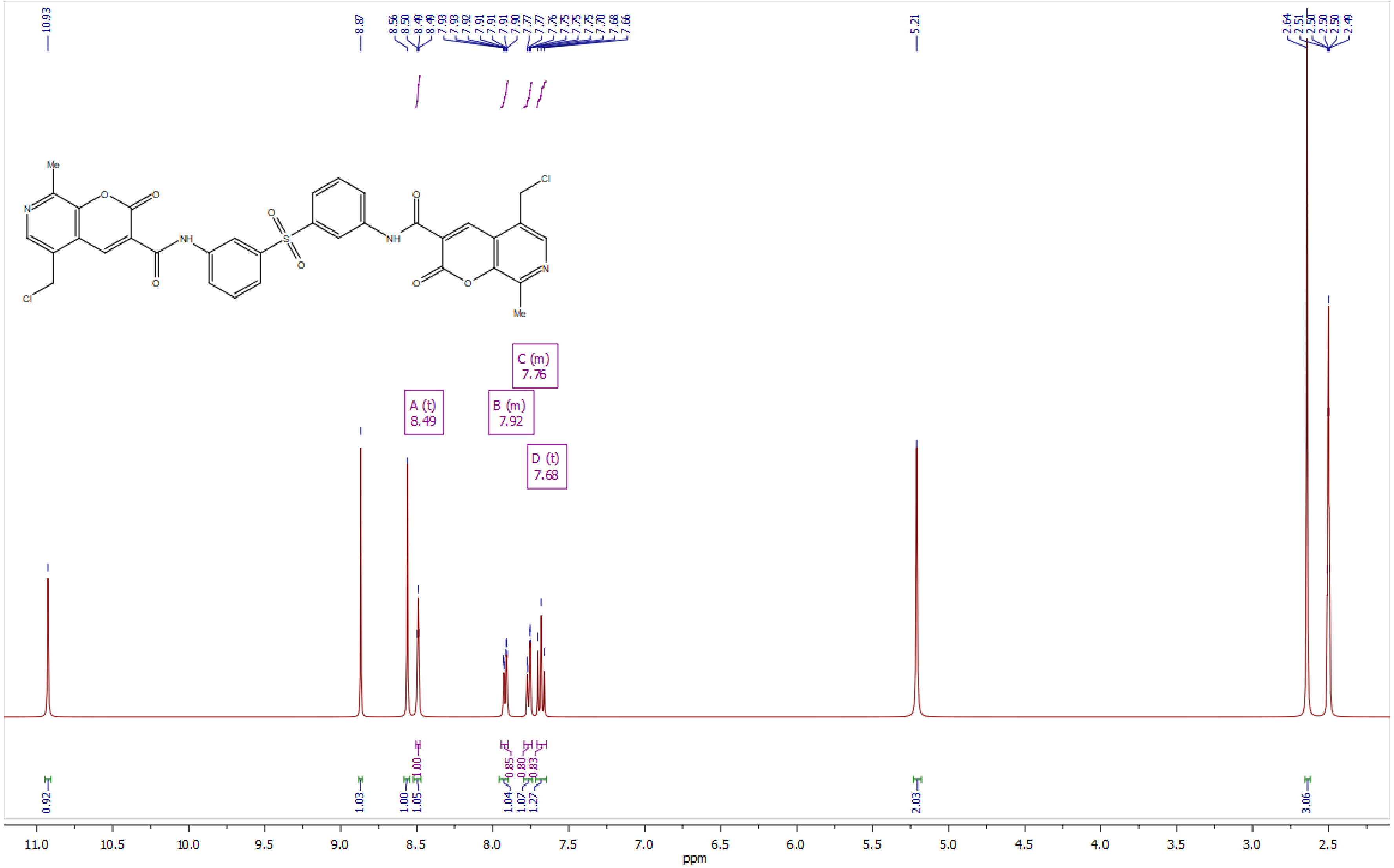

^{1}H NMR (400 MHz, DMSO-d_6) δ 8.49 (t, J = 2.0 Hz, 1H), 7.94 – 7.90 (m, 1H), 7.79 – 7.74 (m, 1H), 7.68 (t, J = 7.9 Hz, 1H).

Figure S54. ^{1}H NMR spectra of the compound **7s** (400 MHz, DMSO-d_6)

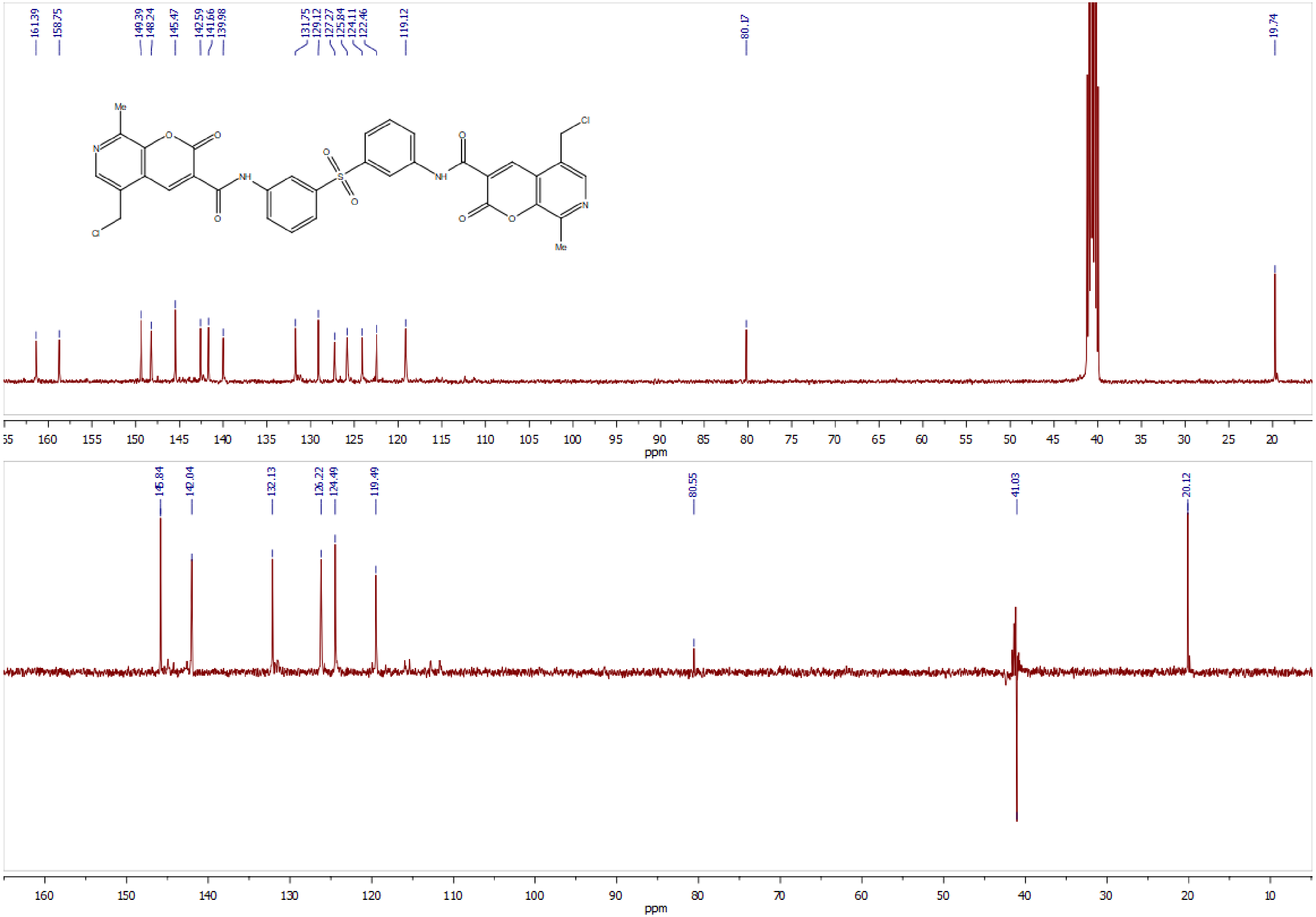

Figure S55. ^{13}C NMR spectra of the compound **7s** (400 MHz, DMSO-d_6)

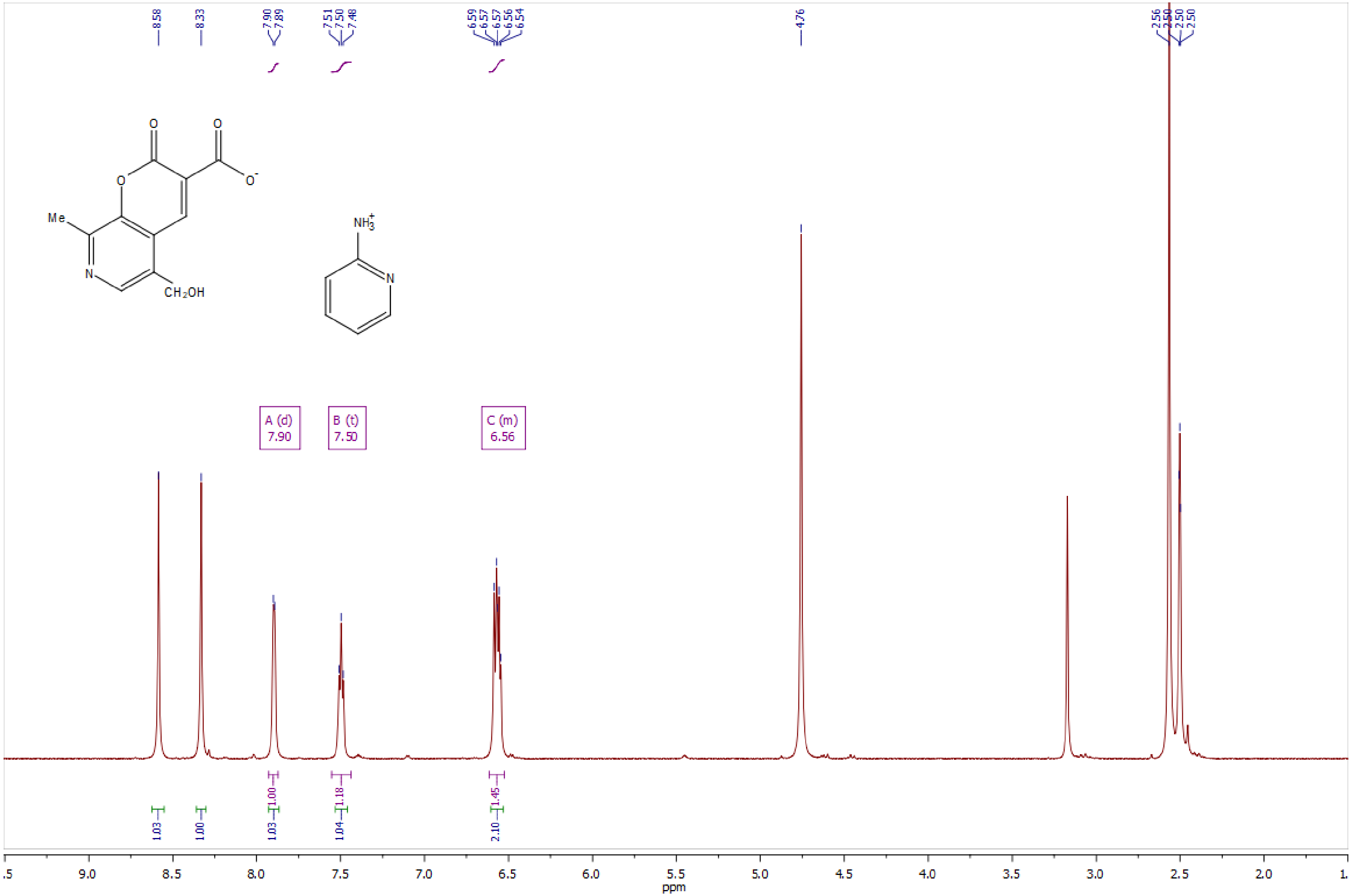

^{1}H NMR (600 MHz, DMSO-d_6) δ 7.90 (d, J = 4.5 Hz, 1H), 7.50 (t, J = 7.0 Hz, 1H), 6.61 – 6.53 (m, 1H).

Figure S56. ^{1}H NMR spectra of the compound **8a** (600 MHz, DMSO-d_6)

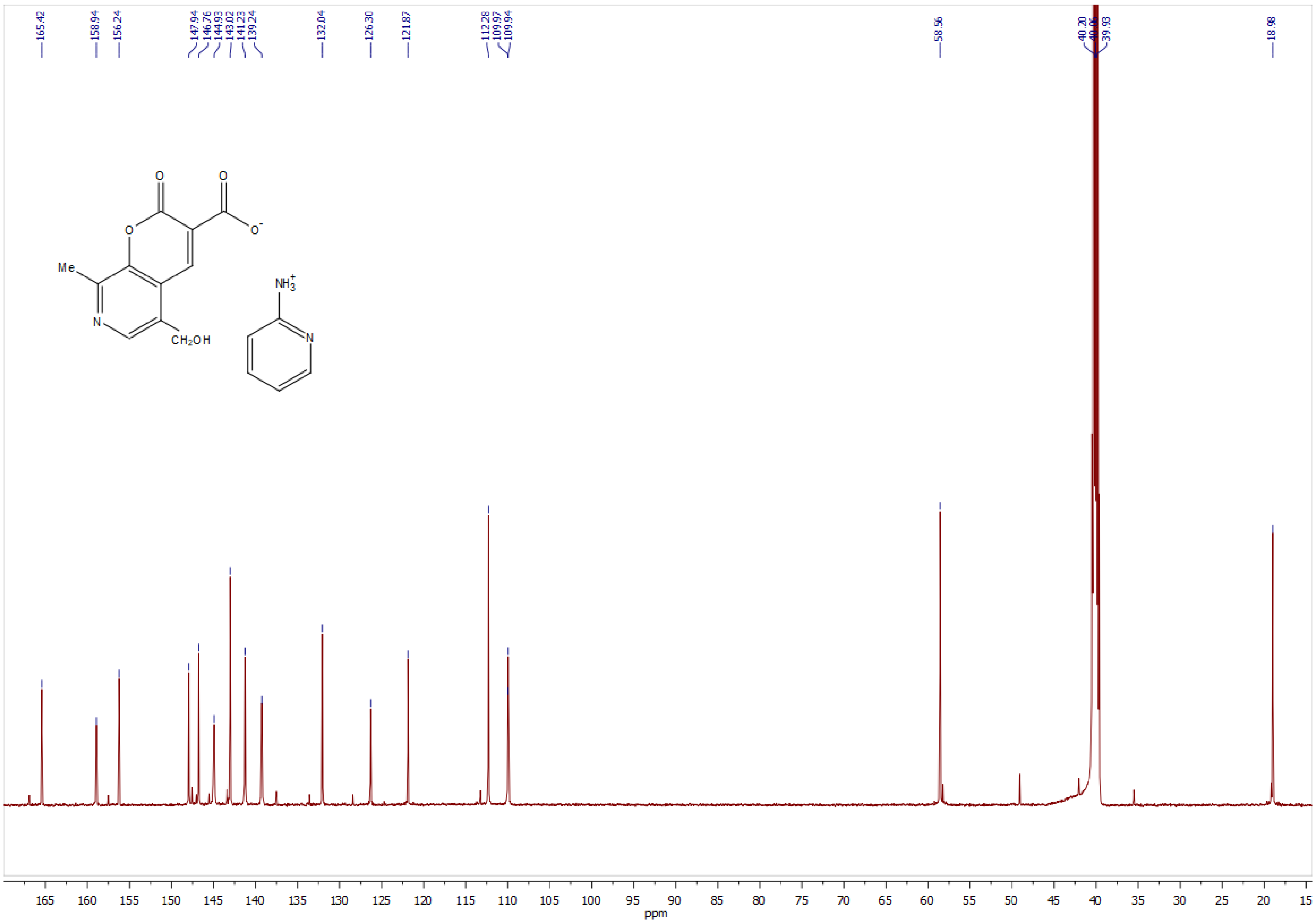

Figure S57. ^{13}C NMR spectra of the compound **8a** (600 MHz, DMSO-d_6)

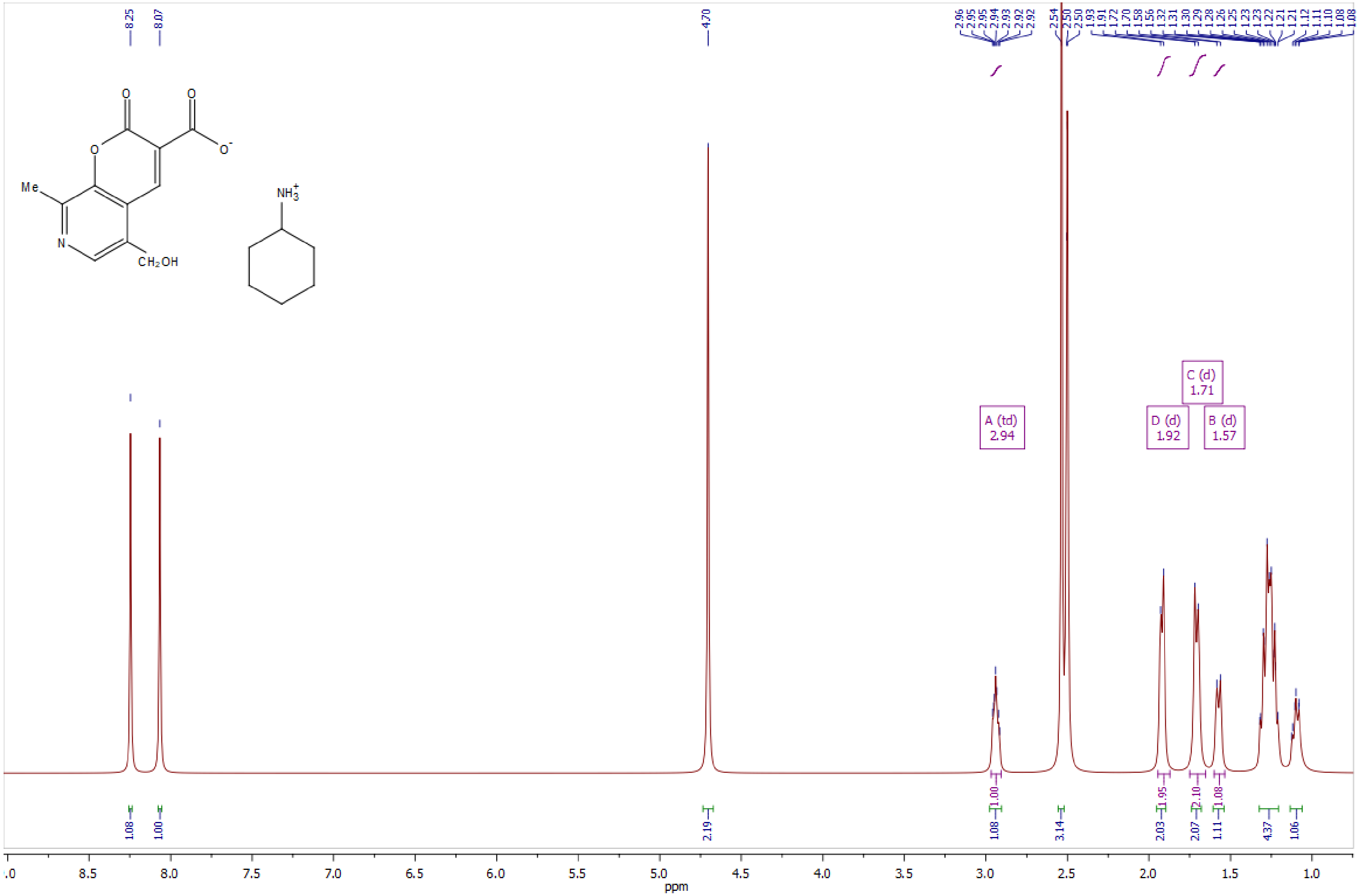

^{1}H NMR (600 MHz, DMSO-d_6) δ 2.94 (td, J = 10.5, 10.0, 5.4 Hz, 1H), 1.92 (d, J = 11.2 Hz, 2H), 1.71 (d, J = 12.2 Hz, 2H), 1.57 (d, J = 13.0 Hz, 1H).

Figure S58. ^{1}H NMR spectra of the compound **8b** (600 MHz, DMSO-d_6)

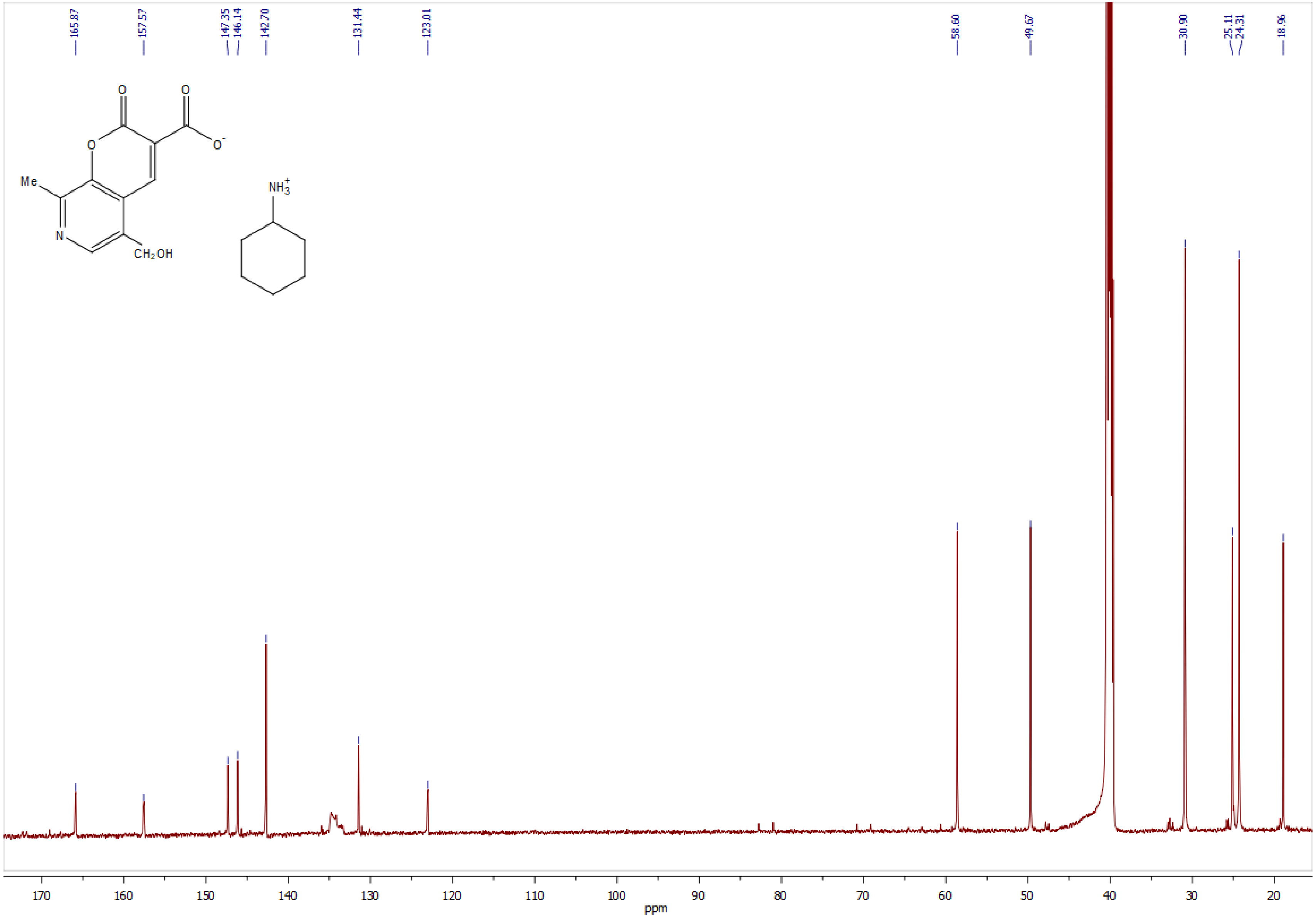

Figure S59. ^{13}C NMR spectra of the compound **8b** (600 MHz, DMSO-d_6)

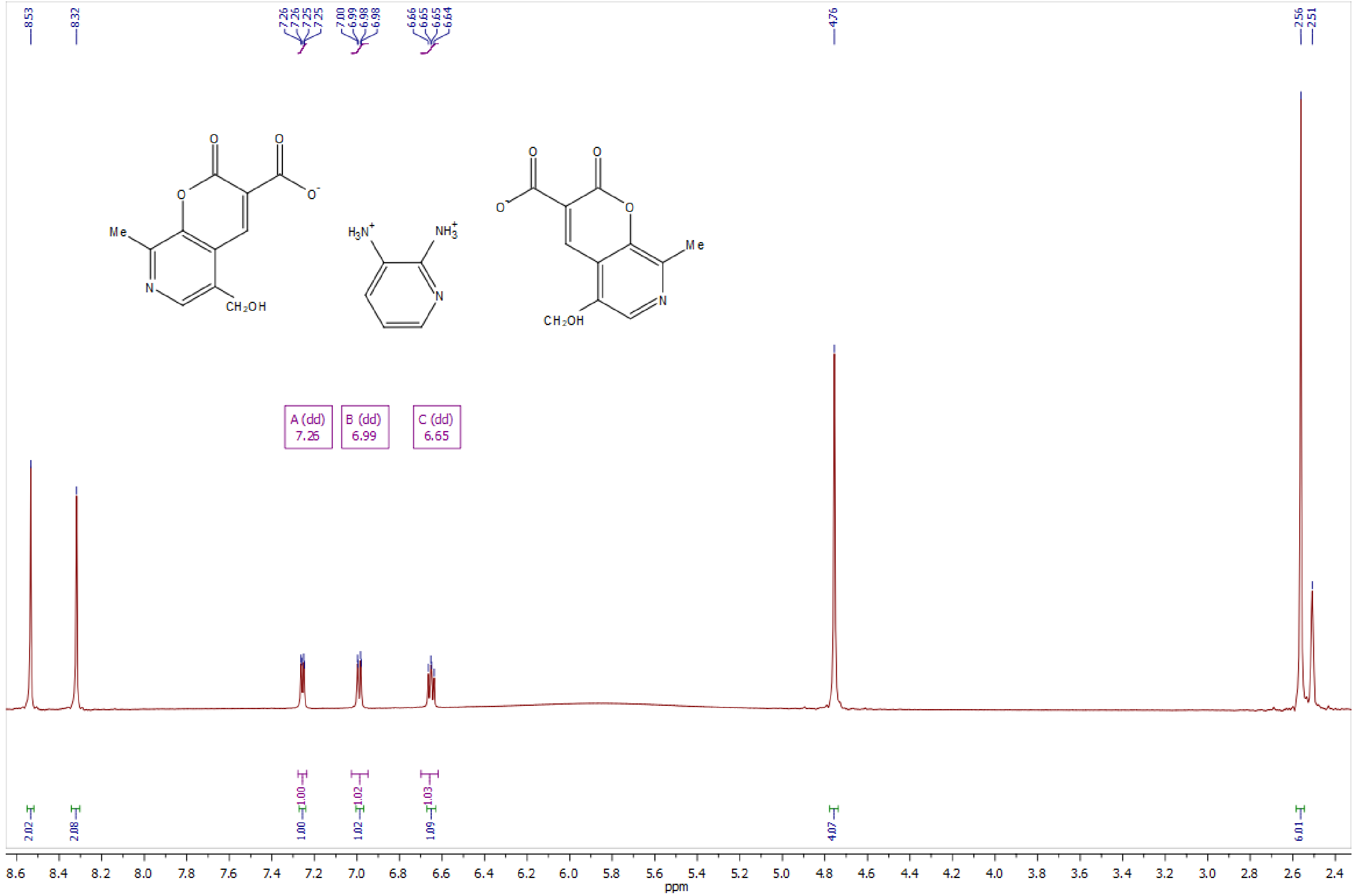

^{1}H NMR (400 MHz, DMSO-d_6) δ 7.26 (dd, J = 6.0, 1.4 Hz, 1H), 6.99 (dd, J = 7.7, 1.4 Hz, 1H), 6.65 (dd, J = 7.6, 5.9 Hz, 1H).

Figure S60. ^{1}H NMR spectra of the compound **8c** (400 MHz, DMSO-d_6)

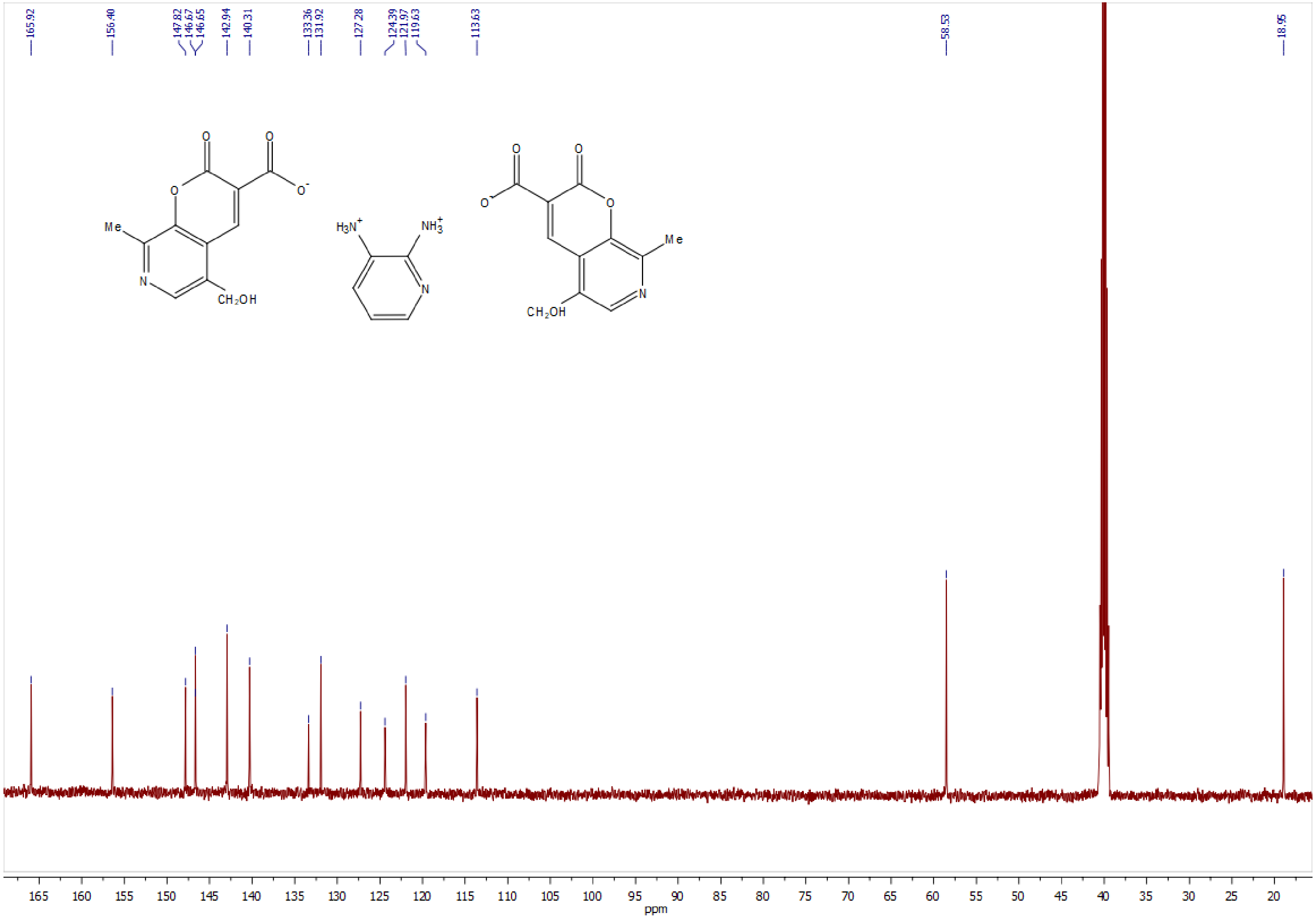

Figure S61. ^{13}C NMR spectra of the compound **8c** (400 MHz, DMSO-d_6)

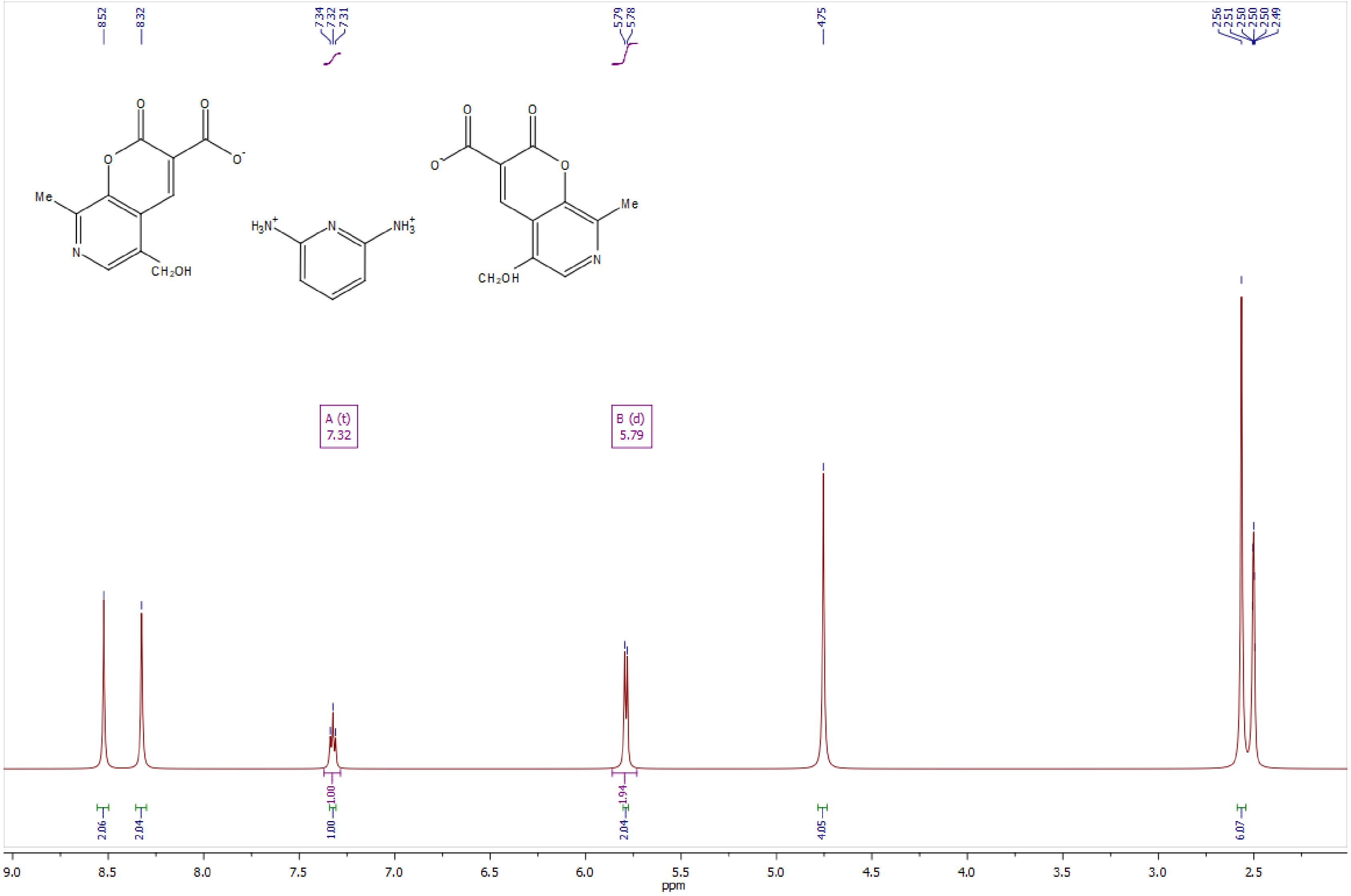

^{1}H NMR (600 MHz, DMSO-$d6$) δ 7.32 (t, J = 8.0 Hz, 1H), 5.79 (d, J = 8.0 Hz, 2H).

Figure S62. ^{1}H NMR spectra of the compound **8d** (600 MHz, DMSO-d_6)

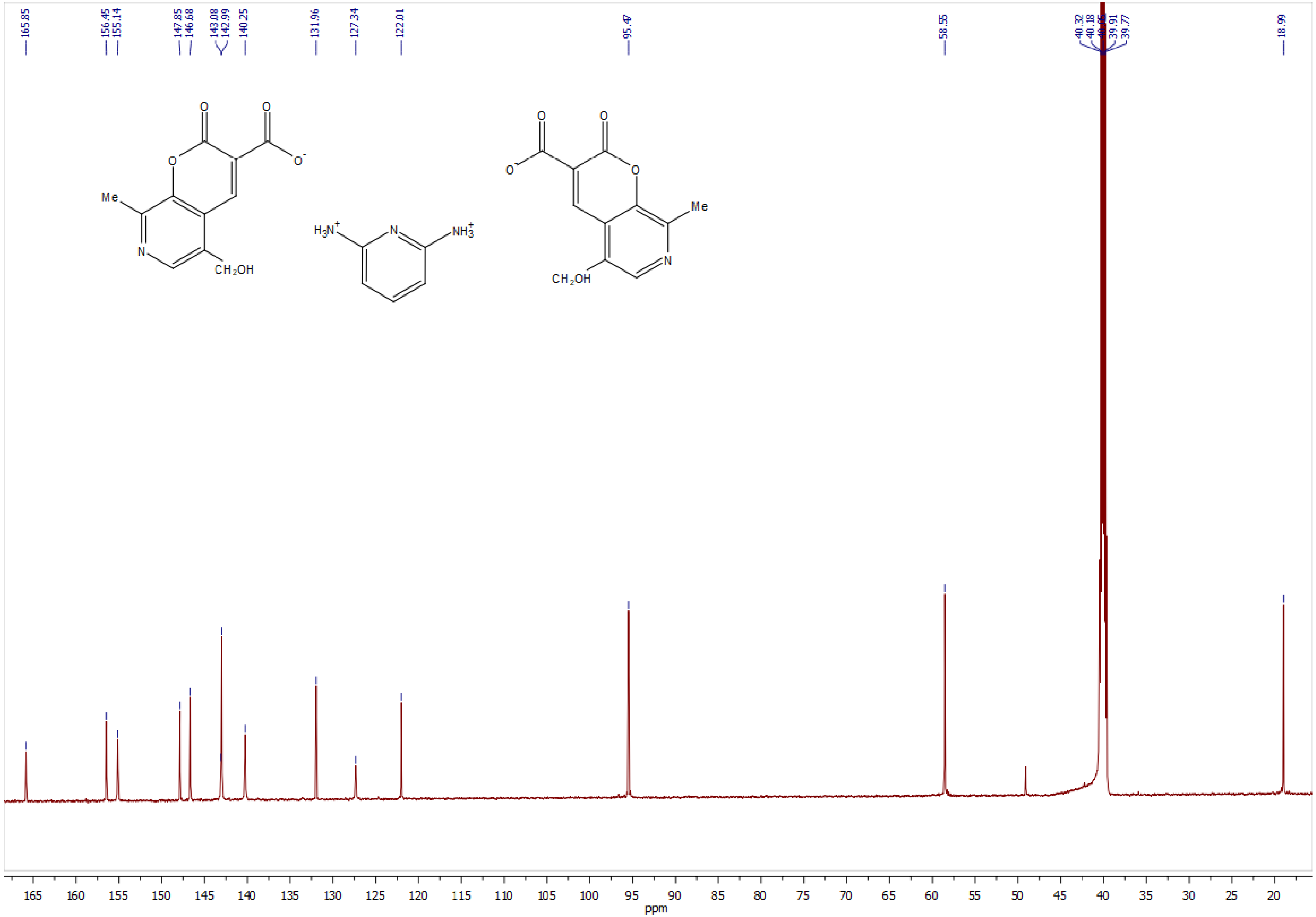

Figure S63. ^{13}C NMR spectra of the compound **8d** (600 MHz, DMSO-d_6)

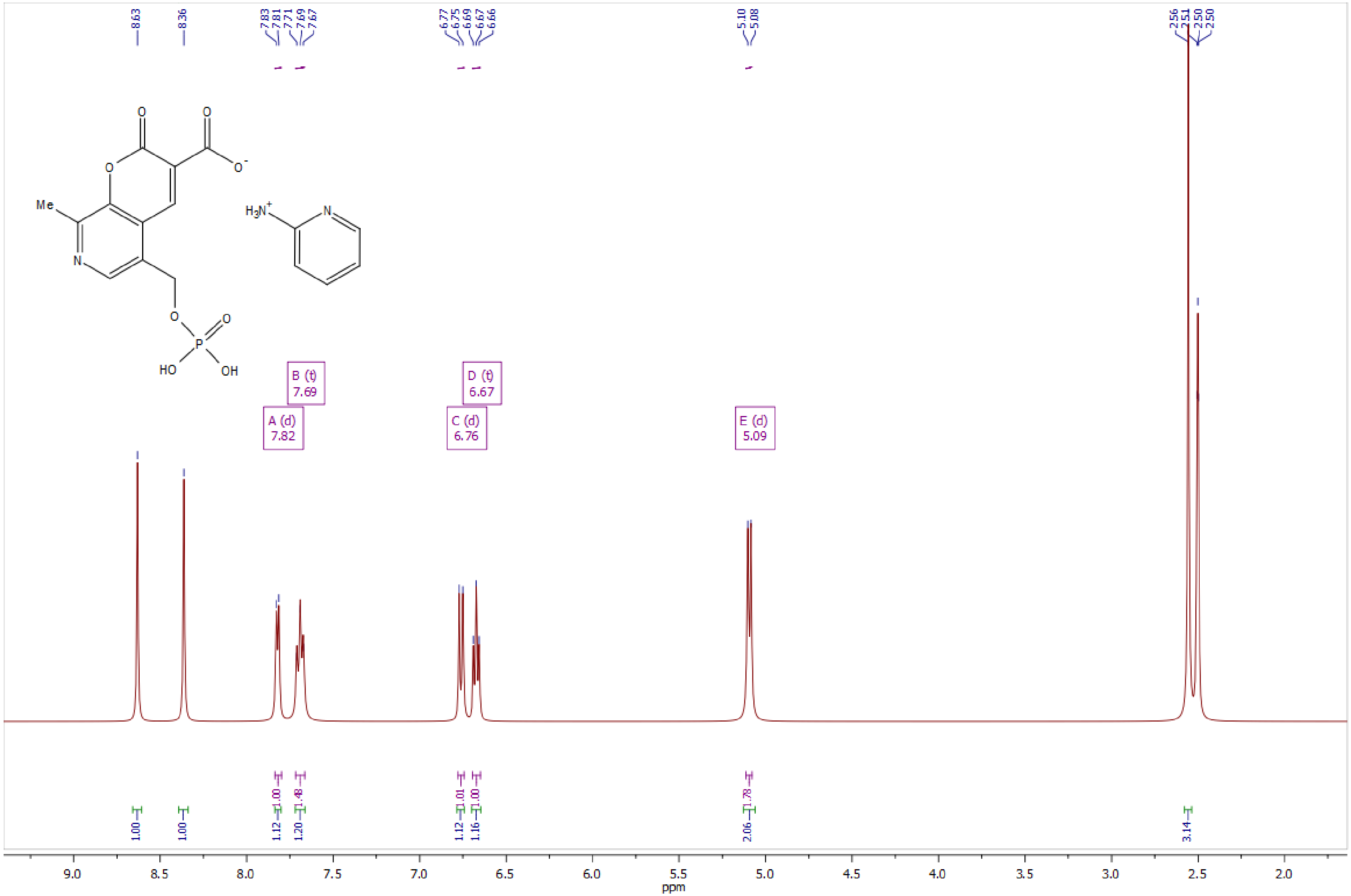

^1H NMR (400 MHz, DMSO-d_6) δ 7.82 (d, J = 6.0 Hz, 1H), 7.69 (t, J = 7.8 Hz, 1H), 6.76 (d, J = 8.7 Hz, 1H), 6.67 (t, J = 6.5 Hz, 1H), 5.09 (d, J = 7.5 Hz, 2H).

Figure S64. ^1H NMR spectra of the compound **8e** (400 MHz, DMSO-d_6)

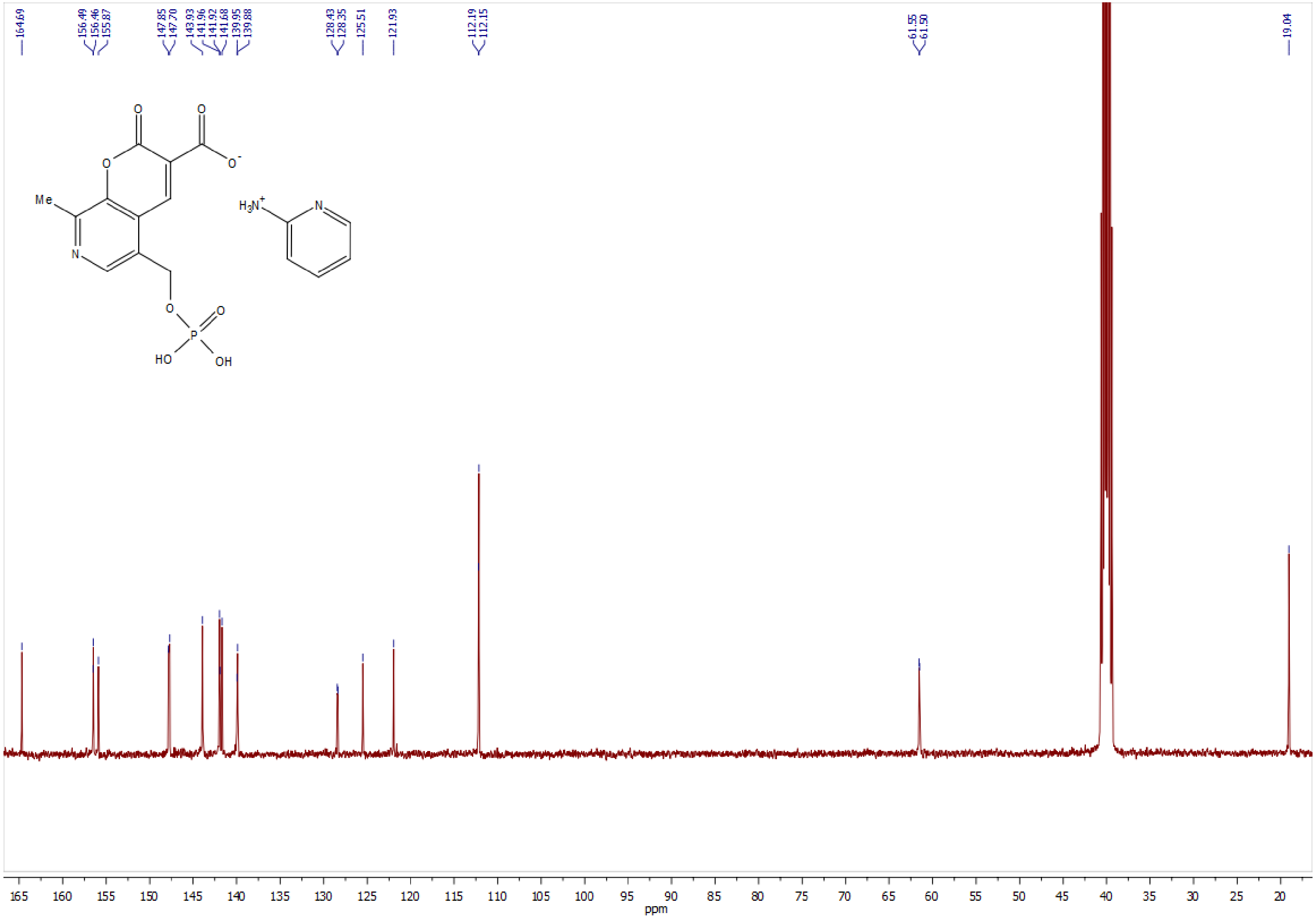

Figure S65. ^{13}C NMR spectra of the compound **8e** (400 MHz, DMSO-d_6)

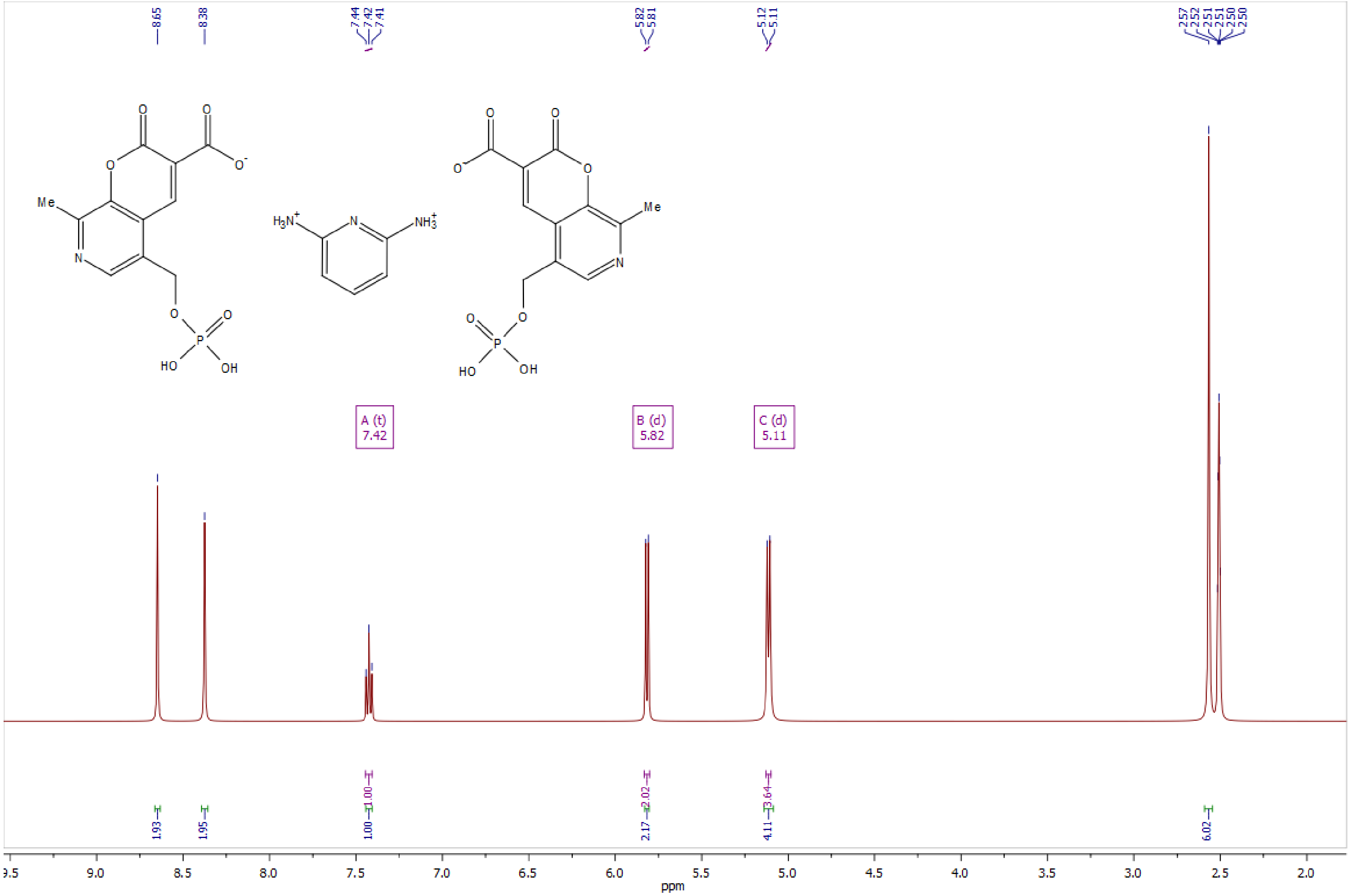

^{1}H NMR (600 MHz, DMSO-d_6) δ 7.42 (t, J = 8.2 Hz, 1H), 5.82 (d, J = 8.2 Hz, 2H), 5.11 (d, J = 7.7 Hz, 4H).

Figure S66. ^{1}H NMR spectra of the compound **8f** (600 MHz, DMSO-d_6)

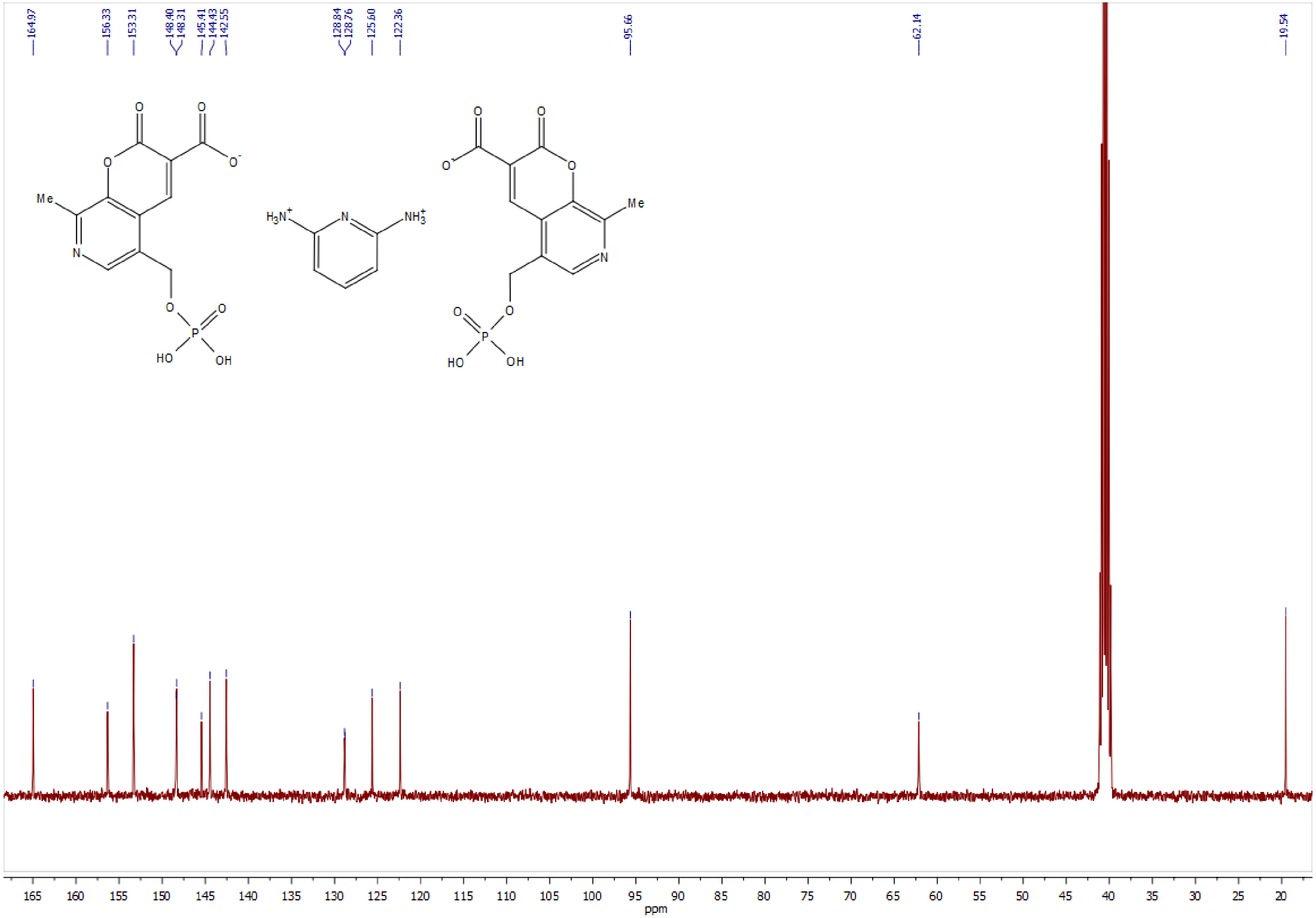

Figure S67. ^{13}C NMR spectra of the compound **8f** (600 MHz, DMSO-d_6)

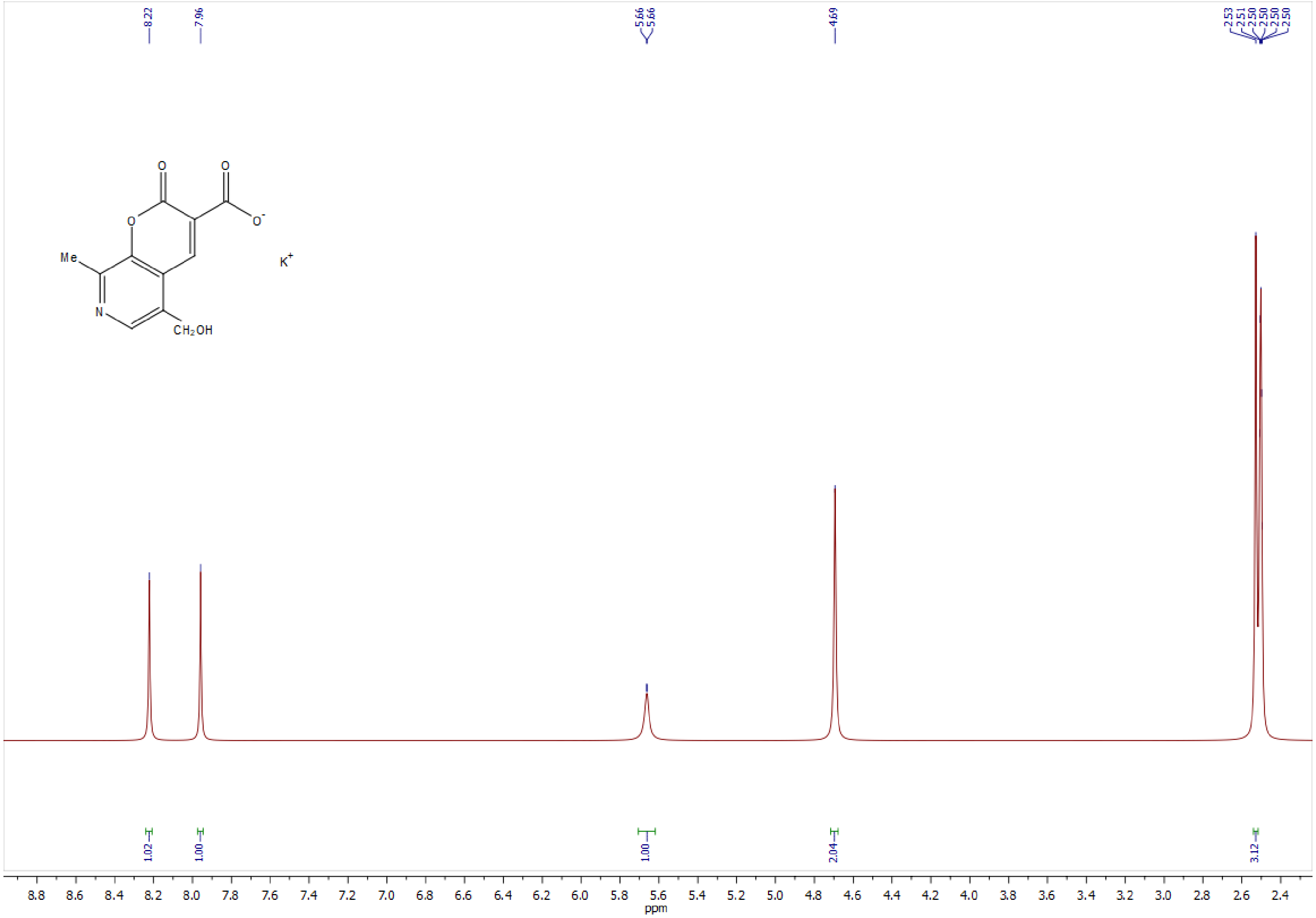

Figure S68. ^{1}H NMR spectra of the compound **S1** (600 MHz, DMSO-d_6)

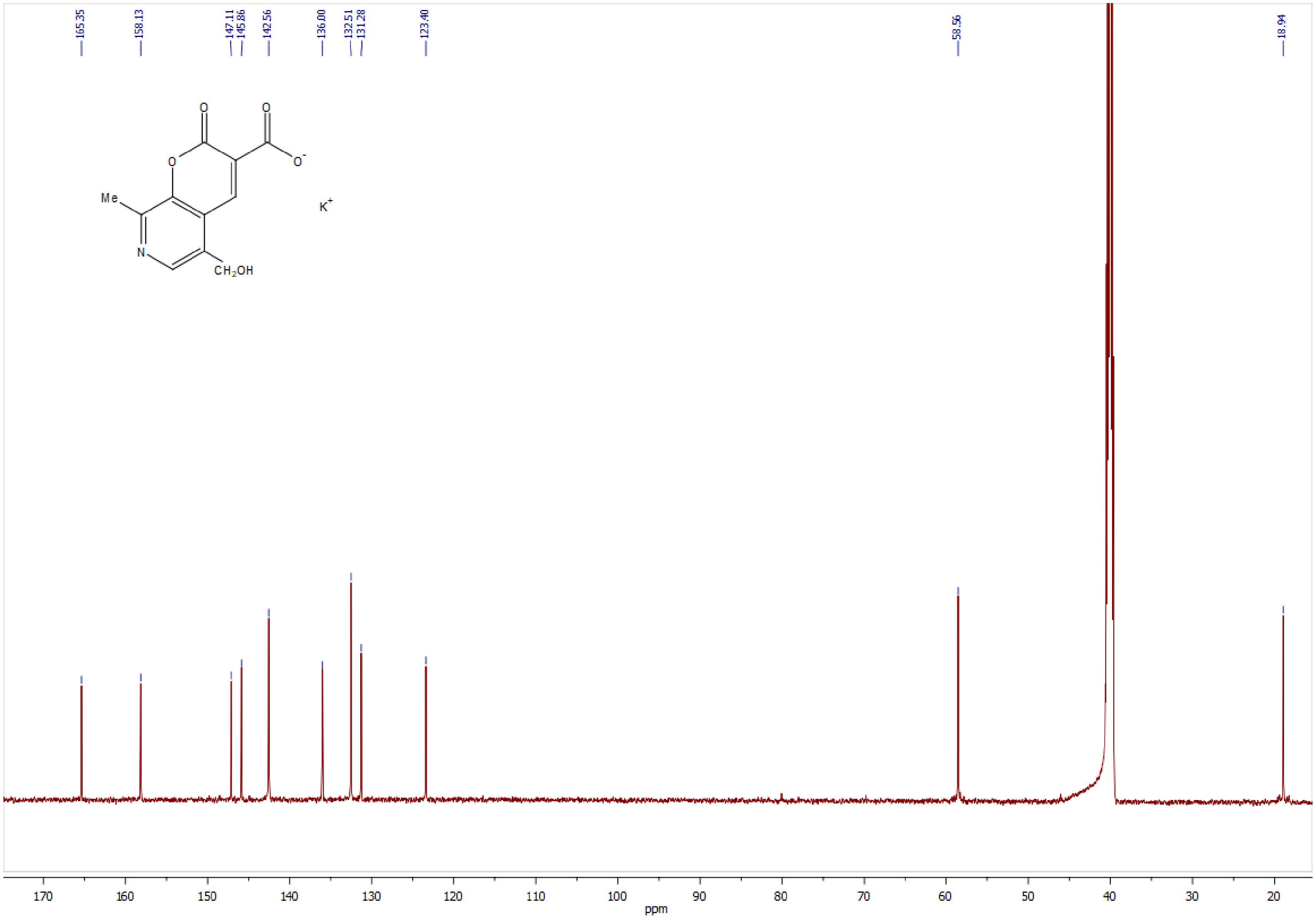

Figure S69. ^{13}C NMR spectra of the compound **S1** (600 MHz, DMSO-d_6)
